# Ribosomal translation of fluorinated non-canonical amino acids for *de novo* biologically active fluorinated macrocyclic peptides†

**DOI:** 10.1039/d4sc04061a

**Published:** 2024-08-02

**Authors:** Junjie Wu, Yuchan Wang, Wenfeng Cai, Danyan Chen, Xiangda Peng, Huilei Dong, Jinjing Li, Hongtan Liu, Shuting Shi, Sen Tang, Zhifeng Li, Haiyan Sui, Yan Wang, Chuanliu Wu, Youming Zhang, Xinmiao Fu, Yizhen Yin

**Affiliations:** a State Key Laboratory of Microbial Technology, Institute of Microbial Technology, Shandong University Qingdao 266237 China zhangyouming@sdu.edu.cn yizhenyin.1987@sdu.edu.cn; b College of Life Sciences, Fujian Normal University Fuzhou 350117 China xmfu@fjnu.edu.cn; c Shanghai Zelixir Biotech Company Ltd Shanghai 200030 China; d College of Chemistry and Chemical Engineering, Xiamen University Xiamen 361005 China; e Shandong Research Institute of Industrial Technology Jinan 250101 China

## Abstract

Fluorination has emerged as a promising strategy in medicinal chemistry to improve the pharmacological profiles of drug candidates. Similarly, incorporating fluorinated non-canonical amino acids into macrocyclic peptides expands chemical diversity and enhances their pharmacological properties, from improved metabolic stability to enhanced cell permeability and target interactions. However, only a limited number of fluorinated non-canonical amino acids, which are canonical amino acid analogs, have been incorporated into macrocyclic peptides by ribosomes for *de novo* construction and target-based screening of fluorinated macrocyclic peptides. In this study, we report the ribosomal translation of a series of distinct fluorinated non-canonical amino acids, including mono-to tri-fluorinated variants, as well as fluorinated l-amino acids, d-amino acids, β-amino acids, *etc.* This enabled the *de novo* discovery of fluorinated macrocyclic peptides with high affinity for EphA2, and particularly the identification of those exhibiting broad-spectrum activity against Gram-negative bacteria by targeting the BAM complex. This study not only expands the scope of ribosomally translatable fluorinated amino acids but also underscores the versatility of fluorinated macrocyclic peptides as potent therapeutic agents.

## Abstract 2

Fluorinated non-canonical amino acids were ribosomally translated, allowing identification of fluorinated macrocyclic peptides with strong binding to EphA2 and broad-spectrum activity against Gram-negative bacteria by targeting the BAM complex.

## Graphical abstract

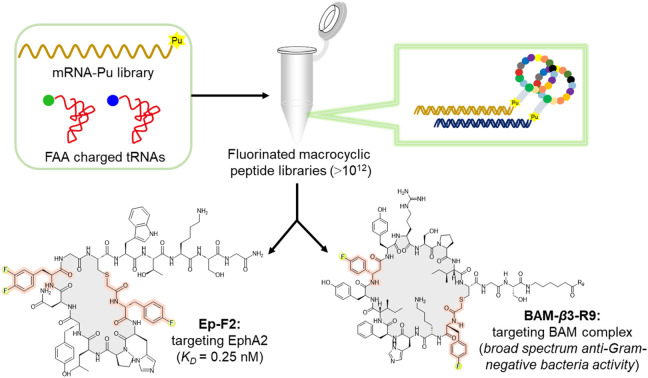

## Introduction

Fluorination has been regarded as a guiding principle in medicinal chemistry for enhancing the pharmacological activity of drug candidates.^1,2^ Currently, more than 20% of drugs on the market contain fluorine atoms, with this proportion expected to reach 30% in the future. This is primarily attributed to several key characteristics of the fluorine atom: its size lies between that of hydrogen and oxygen atoms, allowing for modifications without significantly altering their overall structure or volume; the highly polarized C–F bond increasing the oxidative-reductive stability and metabolic stability; the strong electronegativity capable of affecting molecular properties; and its ability to enhance lipophilicity and improve the permeability of fluorinated compounds.^1,2^

**Fig. 1:**
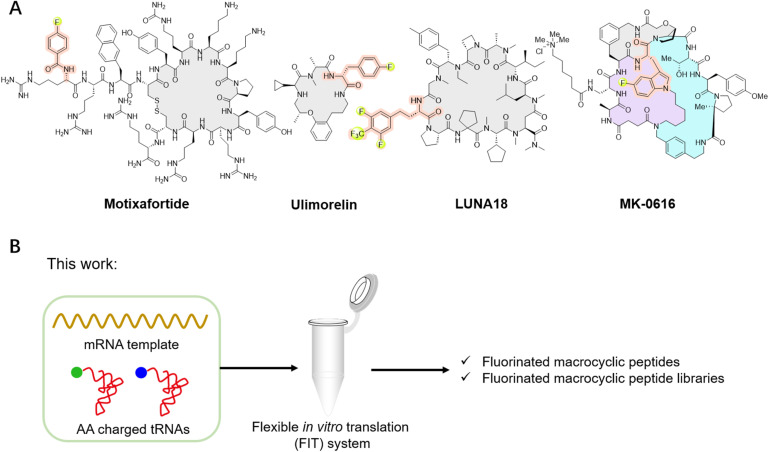
Representative fluorinated macrocyclic peptide drugs and the illustration of this study. (A) Representative fluorinated macrocyclic peptide drugs, such as motixafortide, ulimorelin, MK-0616, and LUNA18. (B) Our work involves the ribosomal translation of fluorinated non-canonical amino acids and the construction of fluorinated macrocyclic peptide libraries for *de novo* discovery of novel fluorinated macrocyclic peptides.

Given the advantages of fluorination, incorporating fluorinated non-canonical amino acids (FAAs) offers various benefits to peptides, from enhancing metabolic stability and refining pharmacokinetic properties to improving lipophilicity and cell permeability.^3–5^ Furthermore, this incorporation may influence peptide conformation and increase interactions with targets, thereby amplifying biological activity.^3–5^ For instance, replacing methyl groups on the side chains of certain dipeptides or tripeptides with trifluoromethyl groups can significantly enhance the transmembrane properties of peptides, with enhancements reaching up to a remarkable 72 times.^6^ In a specific case, replacing alanine (A8) in glucagon-like peptide-1 (GLP-1) with 5,5,5,5′,5′,5′-hexafluoroleucine yields the fluorinated peptide F8, which retains a considerable degree of GLP-1 activity and exhibits remarkable resistance to degradation by dipeptidyl peptidase-4 (DPP-IV) over a 24 hour period.^7^ Substituting a phenylalanine in bicyclic peptide inhibitors of coagulation factor XII with 4-fluoro-l-phenylalanine increases the inhibitory activity by nearly 10-fold, albeit with a slight reduction in stability.^8^ Among the fluorinated peptide drugs available in the market, glecaprevir and voxilaprevir have been approved as pan-genotypic hepatitis C direct-acting antiviral drugs.^5,9^ Meanwhile, motixafortide has been approved for the treatment of pancreatic cancer in both the European Union and the United States (Fig. 1A), as well as for the treatment of acute myeloid leukemia in the United States.^10^ Ulimorelin (Fig. 1A), containing 4-fluoro-d-phenylalanine, acts as a human Ghrelin (GRLN) receptor agonist, currently in phase III clinical trials for the treatment of postoperative intestinal obstruction.^3,11^ Another notable example is MK-0616 (Fig. 1A), a fluorinated tricyclic peptide targeting proprotein convertase subtilisin/kexin type 9 (PCSK9).^12–14^ This compound inhibits the interaction between PCSK9 and low-density lipoprotein (LDL) receptors, undergoing phase III clinical trials as a potential oral inhibitor for adults with hypercholesterolemia. Recently, the development of LUNA18 (Fig. 1A), a highly bioavailable fluorinated macrocyclic peptide KRAS inhibitor, has garnered significant attention, representing a promising advancement in the field.^15,16^ Additionally, fluorinated peptides can serve as valuable tools in positron emission tomography (PET) and ^19^F nuclear magnetic resonance spectroscopy.^17–20^ These studies highlight the benefits of fluorination to peptides. While peptides can be fluorinated during post-modification process by carefully selecting the positions for fluorine incorporation, there remains significant interest in *de novo* fluorinated peptides.

Macrocyclic peptides have garnered considerable attention due to their promising potential to rival the binding affinity and high specificity of biologics, while also offering the synthetic feasibility and potential for cell permeability and bioavailability characteristic of small molecules.^10,21–32^ Advancements have been made for *de novo* macrocyclic peptides that can specifically bind to desired molecular targets through the utilization of mRNA display technology. Furthermore, the flexible *in vitro* translation (FIT) system can be integrated with mRNA display to give the Random non-standard Peptide Integrated Discovery (RaPID) system.^33–38^ The RaPID system makes it feasible to screen natural products like macrocyclic peptides incorporating non-canonical amino acids, greatly expanding the chemical diversity of macrocyclic peptides.^33–38^ Taking the advantages of both fluorine and mRNA display, fluorinated peptides have been *de novo* discovered, by introducing fluorinated non-canonical amino acids in place of their corresponding natural counterparts within the protein synthesis using recombinant elements (PURE) system. This was achieved by the misincorporation of the canonical amino acid analogs, such as mono-fluorinated l-tyrosine, l-phenylalanine or l-tryptophan, relying on the promiscuity of *E. coli* tRNA synthetase.^39,40^ This approach has led to the successful identification of fluorinated macrocyclic peptides, such as the hit compound of MK-0616. However, it also limits the diversity of available fluorinated non-canonical amino acids that can be included into macrocyclic peptides.

**Fig. 2:**
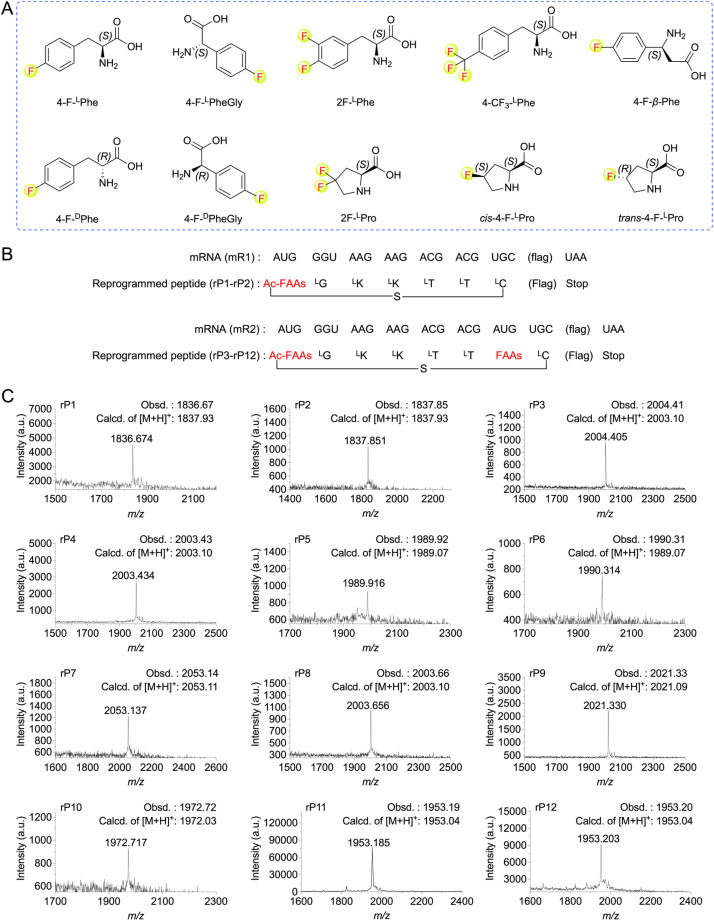
Ribosomal incorporation of FAAs into model peptides. (A) Structures of the FAAs used in this study. (B) mRNAs (mR1–mR2) and the corresponding peptide sequences (rP1–rP12) used for the FAAs incorporation at AUG codons. The amino acid sequence of “flag” is DYKDDDDK. (C) MALDI-TOF mass spectra of model peptides containing FAAs. For synthesis of macrocyclic peptide rP1, ClAc-4-F-^L^Phe were pre-charged on tRNA^fMet^_CAU_. For rP2, ClAc-4-F-^D^Phe were pre-charged on tRNA^fMet^_CAU_. For rP3, ClAc-4-F-^L^Phe and 4-F-^L^Phe pre-charged on tRNA^fMet^_CAU_ and tRNA^Asn-E2^_CAU_, respectively. For rP4, ClAc-4-F-^L^Phe and 4-F-^D^Phe pre-charged on tRNA^fMet^_CAU_ and tRNA^Asn-E2^_CAU_, respectively. For rP5, ClAc-4-F-^L^Phe and 4-F-^L^PheGly pre-charged on tRNA^fMet^_CAU_ and tRNA^Asn-E2^_CAU_, respectively. For rP6, ClAc-4-F-^L^Phe and 4-F-^D^PheGly pre-charged on tRNA^fMet^_CAU_ and tRNA^Asn-E2^_CAU_, respectively. For rP7, ClAc-4-F-^L^Phe and 4-CF_3_-^L^Phe pre-charged on tRNA^fMet^_CAU_ and tRNA^Asn-E2^_CAU_, respectively. For rP8, ClAc-4-F-^L^Phe and 4-F-β-Phe pre-charged on tRNA^fMet^_CAU_ and tRNA^Asn-E2^_CAU_, respectively. For rP9, ClAc-4-F-^L^Phe and 2F-^L^Phe pre-charged on tRNA^fMet^_CAU_ and tRNA^Asn-E2^_CAU_, respectively. For rP10, ClAc-4-F-^L^Phe and 2F-^L^Pro pre-charged on tRNA^fMet^_CAU_ and tRNA^Pro1E2^_CAU_, respectively. For rP11, ClAc-4-F-^L^Phe and *cis*-4-F-^L^Pro pre-charged on tRNA^fMet^_CAU_ and tRNA^Pro1E2^_CAU_, respectively. For rP12, ClAc-4-F-^L^Phe and *trans*-4-F-^L^Pro pre-charged on tRNA^fMet^_CAU_ and tRNA^Pro1E2^_CAU_, respectively.

In this study, we utilized the FIT system and accomplished the ribosomal translation of a series of distinct fluorinated non-canonical amino acids (Fig. 1B and 2A). This enabled us to adopt the RaPID system for the identification of fluorinated macrocyclic peptides capable of strongly binding to human ephrin type-A receptor 2 (EphA2), with *K*_D_ values within the low nanomolar to sub-nanomolar range. Additionally, we identified fluorinated macrocyclic peptides that demonstrate a broad-spectrum of activity against Gram-negative bacteria by targeting the β-barrel assembly machinery (BAM) complex.

## Results and discussion

### Ribosomal translation of fluorinated non-canonical amino acids using the FIT system

To expand the translation scope by ribosome for *de novo* fluorinated macrocyclic peptides, we attempted to achieve ribosomal translation of various FAAs using the FIT system. These amino acids included mono-to tri-fluorinated variants, as well as fluorinated l-amino acids, d-amino acids, β-amino acids, *etc* (Fig. 2A). At first, we contemplated utilizing a type of fluorinated non-canonical amino acid to serve both as an initiator and a functional moiety for macrocyclization. Consequently, we first chemically synthesized *N*-(2-chloroacetyl)-4-fluoro-l(or d)-phenylalanine cyanomethyl ester(ClAc-4-F-^L(or D)^Phe-CME). Subsequently, we estimated the efficiency of their aminoacylation onto a shortened tRNA analog called microhelix RNA (μhRNA), catalyzed by the enhanced flexizyme (eFx), through acid-denaturing polyacrylamide gel electrophoresis (PAGE) (Fig. S1†). They were effectively charged onto μhRNA and then allowed for preparation of ClAc-4-F-^L(or D)^Phe-tRNA^fMet^_CAU_. The initiation position was reprogrammed to ClAc-4-F-^L(or D)^Phe-tRNA^fMet^_CAU_ in a methionine (Met) deficient FIT system that included an mRNA template (mR1) (Fig. 2B). The successful expression of the desired thioether cyclized macrocyclic peptides (rP1 and rP2) was confirmed through matrix-assisted laser desorption/ionization time-of-flight mass spectrometry (MALDI-TOF MS) analysis (Fig. 2C).

Having determined the ribosomal expression of ClAc-4-F-^L(or D)^Phe at the initiation position, our next step was to investigate whether the fluorinated non-canonical amino acids could be incorporated into peptides at the elongation sites. Thus, 4-fluoro-l(or d)-phenylalanine cyanomethyl ester (4-F-^L(or D)^Phe-CME), 4-fluoro-l(or d)-phenylglycine cyanomethyl ester (4-F-^L(or D)^PheGly-CME), 3,4-difluoro-l-phenylalanine cyanomethyl ester (2F-^L^Phe-CME), 4-trifluoromethyl-l-phenylalanine cyanomethyl ester (4-CF_3_-^L^Phe-CME) and 4-fluoro-l-β-phenylalanine cyanomethyl ester (4-F-β-Phe-CME) were chemically synthesized and subsequently applied to determine the efficiency of their aminoacylation onto μhRNA using eFx. It was determined that the aminoacylation efficiency of all the building blocks reached an acceptable level after optimizing the reaction time (over 20%) (Fig. S1 and S2†), enabling them to be charged onto engineered tRNAs (tRNA^Asn-E2^_CAU_). In the case of 4,4-difluoro-l-proline (2F-^L^Pro), *cis*-4-fluoro-l-proline (*cis*-4-F-^L^Pro) and *trans*-4-fluoro-l-proline (*trans*-4-F-^L^Pro), we proceeded to convert them into their corresponding 3,5-dinitrobenzyl esters, after which we evaluated and optimized their aminoacylation efficiency using the dinitroflexizyme (dFx) (Fig. S1 and S2†). Subsequently, they were charged onto tRNA^Pro1E2^_CAU_. This was followed by *in vitro* translation using mR2 as an mRNA template (Fig. 2B), with the initiation position reprogrammed to ClAc-4-F-^L^Phe. All desired peptides were successfully expressed as confirmed by MALDI-TOF MS analysis (Fig. 2C), indicating that the ribosome and elongation factors could tolerate and accommodate these fluorinated non-canonical amino acids.

### *De novo* fluorinated macrocyclic peptides targeting EphA2

**Fig. 3:**
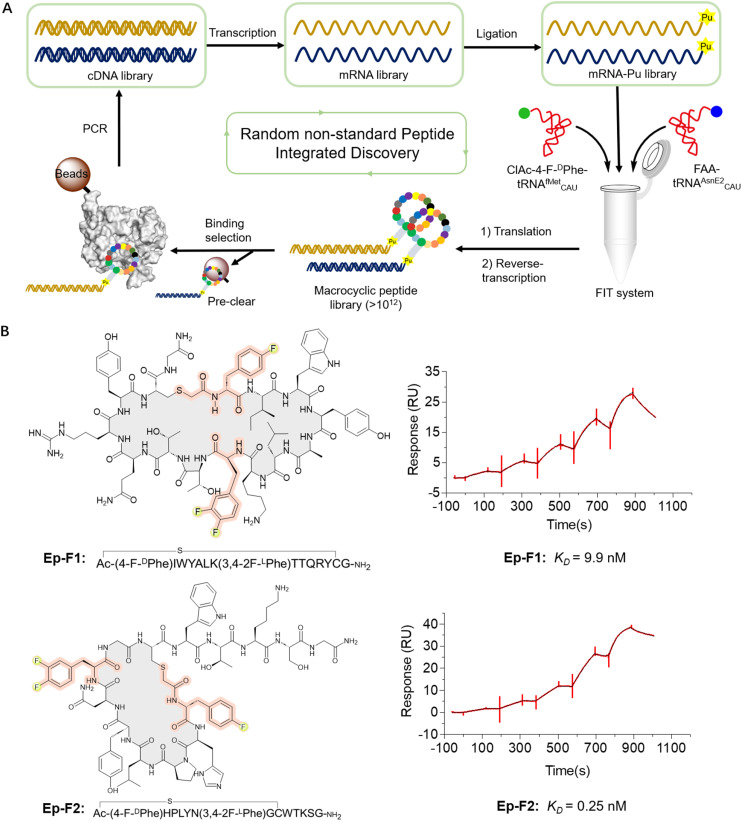
*De novo* discovery of fluorinated macrocyclic peptides targeting EphA2. (A) Selection of fluorinated macrocyclic peptides using the RaPID system. (B) Chemical structures and SPR results of the selected top two fluorinated macrocyclic peptides, Ep-F1 and Ep-F2. Five different concentrations (Ep-F1: 3.1, 6.3, 12.5, 25, 50 nM; Ep-F2: 0.5, 1.0, 2.5, 5, 10 nM) were injected for SPR measurements.

Next, we constructed an mRNA library consisting of AUG–(NNK)_5–7_–AUG–(NNK)_5_–UGC–(GGC–AGC)_3_–UAG, where NNK represents random sequence assigned by N (any of four bases) and (U or G). The initiator AUG codon was reassigned to ClAc-4-F-^D^Phe, while the elongator UGC codon encoded a cysteine (Cys) for the formation of the thioether macrocyclic peptide. The elongator AUG codon was reprogrammed to incorporate a fluorinated non-canonical amino acid. Moreover, the AUG codon may appear randomly in the NNK region as well, leading to the potential for incorporation of multiple fluorinated non-canonical amino acids in peptides. This mRNA library was subsequently ligated to a puromycin-CC-PEG-linker-DNA fragment followed by addition into the Met and RF1 (release factor 1) omitted FIT system. The (GGC–AGC)_3_ segment encoded a glycine–serine (GS) triple-repeat peptide linker to endow flexibility, while the UAG codon could induce ribosome stalling and facilitate efficient puromycin-peptide fusion. It was expected that over 10^12^ unique sequences would be expressed in the library (Fig. 3A). We first expressed an mRNA displayed fluorinated macrocyclic peptide library by reassigning the initiation position to ClAc-4-F-^D^Phe and elongator AUG codon to 2F-^L^Phe. Subsequently, we subjected the pool to panning against EphA2, which is a potential new therapeutic target in cancer because of its high expression levels in a lot of tumors. The remarkable enrichment was observed at the fifth round of the selection (Fig. S3†), and enriched cDNA library was analyzed by DNA deep sequencing. According to the hit frequency and sequence consensus, we selected the top two macrocyclic sequences, chemically synthesized them using solid-phase peptide synthesis, and assessed their binding affinities to EphA2 by surface plasmon resonance (SPR). The peptides exhibited their strong binding affinities to EphA2 with the *K*_D_ values at 9.9 nM for Ep-F1 and 0.25 nM for Ep-F2 (Fig. 3B). Although fluorination was shown not to play a critical role in the binding of Ep-F2 to EphA2 (Fig. S4†), it is still expected to enhance the pharmacokinetic properties of the peptides. Fluorinated macrocyclic peptides that bind specifically to EphA2 could serve as targeted therapies, delivering cytotoxic agents directly to cancer cells. Additionally, these peptides could be valuable for diagnostic imaging and other biomedical applications. This also encouraged us to further explore the other fluorinated macrocyclic peptide libraries using different fluorinated non-canonical amino acids.

### *De novo* fluorinated macrocyclic peptides against Gram-negative bacteria

With infections caused by Gram-negative bacteria becoming increasingly prevalent and difficult to treat, there is an urgent need for novel therapeutics capable of effectively combating these pathogens, particularly against multidrug-resistant strains.^22,41–45^ The BAM complex is a vital molecular machinery in Gram-negative bacteria, consisting of multiple protein subunits including BamA, BamB, BamC, BamD, and BamE. This complex is responsible for the correct folding and insertion of β-barrel proteins into the outer membrane, contributing to membrane integrity, cell viability, and bacterial pathogenesis. Importantly, the BAM complex is highly conserved in Gram-negative bacteria, making it a potential broad-spectrum antibacterial target.^22,41–45^ Thus, we conducted *de novo* selections of fluorinated macrocyclic peptides targeting the BAM complex using two libraries, where the initiation codon reassigned to ClAc-4-F-^D^Phe and the elongator AUG codon reprogrammed to either 4-F-^D^Phe or 4-F-β-Phe (Fig. S5 and S6†). The enriched cDNA pools underwent deep sequencing followed by chemical synthesis of four fluorinated macrocyclic peptides (two peptides from each library), which were then utilized for the subsequent evaluation steps. Initially, we directly assessed the antibacterial activity of these peptides against *E. coli*. Unfortunately, no activity was observed (Fig. S7A†). However, two peptides demonstrated activity in the presence of 1 mM ethylenediaminetetraacetic acid (EDTA) (Fig. S7B†).^46–48^ We speculated that the peptides might exert their activity by targeting the intracellular domains of the BAM complex instead of the extracellular domains, with the assistance of EDTA for facilitating the penetration of peptides through the membrane. Moreover, these two fluorinated macrocyclic peptides, BAM-f2 and BAM-β3, were also shown to display low to negligible hemolytic toxicity towards mouse red blood cells (Fig. S8A†). Using SPR measurements, we determined that BAM-f2 and BAM-β3 indeed bind to the BAM complex, with *K*_D_ values of 754.9 nM and 453.4 nM, respectively (Fig. S9 and Table S1†).

**Fig. 4:**
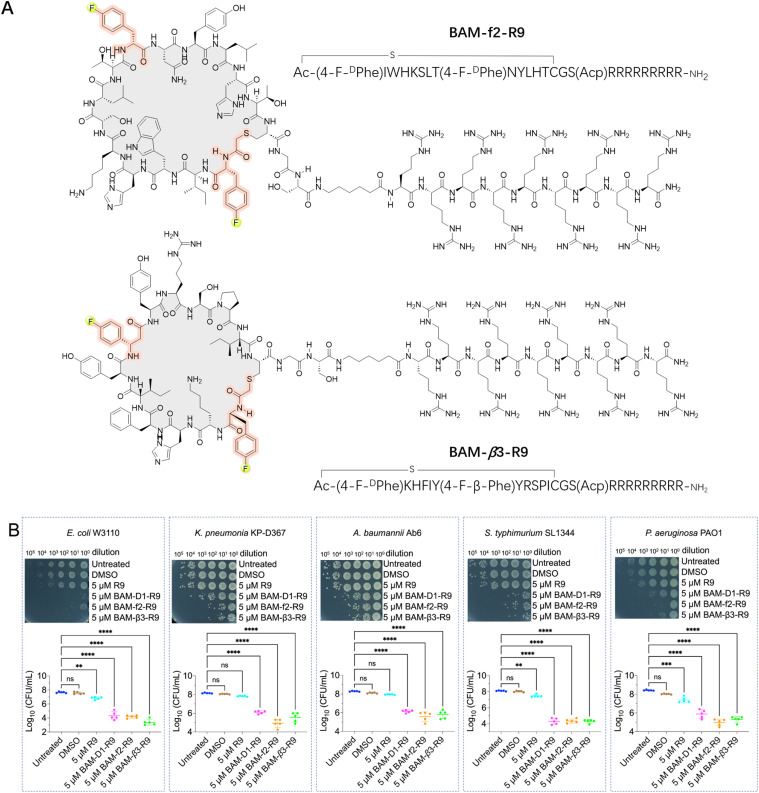
Fluorinated macrocyclic peptides conjugated with a cell penetration peptide (R9) for the treatment of Gram-negative pathogens. (A) Chemical structures of the selected fluorinated macrocyclic peptides conjugated with R9, BAM-f2-R9 and BAM-β3-R9, which exhibited their inhibitory activity against Gram-negative pathogens. (B) Survival of *E. coli* W3110 cells, *K. pneumonia* KP-D367 cells, *A. baumannii* Ab6 cells, *S. typhimurium* SL1344 cells, and *P. aeruginosa* PAO1 cells in logarithmic-phase following a 15 min treatment with DMSO, 5 μM R9, 5 μM BAM-D1-R9, 5 μM BAM-f2-R9 and BAM-β3-R9. ns: no significance. **, *** and **** indicates a *P*-value of <0.01, <0.001 and <0.0001, respectively, representing the significance levels between antibiotic treatment and untreated groups.

To improve the membrane penetration and enable the macrocyclic peptides to independently demonstrate antibacterial activity, we conjugated a cell-penetrating peptide, nona-arginine (R9), with the macrocyclic peptides. The R9 peptide has been shown to exhibit low anti-Gram-negative bacterial activity, however, its highly positive charge allows it to bind strongly to membranes and cross membranes *via* direct translocation. Furthermore, conjugating the R9 peptide to antimicrobial peptides enhances antibacterial activity, particularly increasing effectiveness against Gram-negative bacteria.^49–51^ In our study, the conjugation led to the synthesis of BAM-f2-R9 and BAM-β3-R9 (Fig. 4A). Interestingly, we observed that treatment with 5 μM of BAM-f2-R9 and BAM-β3-R9 resulted in the eradication of over 99.9% of the *E. coli* bacteria (Fig. 4B). Encouraged by this result, we next further evaluated the efficacy of the R9-conjugated macrocyclic peptides against multidrug-resistant Gram-negative bacterias, including *Pseudomonas aeruginosa* PAO1, *Klebsiella pneumoniae* KP-D367, *Acinetobacter baumannii* Ab6, and *Salmonella* SL1344. The results demonstrated broad-spectrum antimicrobial activity, achieving over 99% antibacterial effectiveness (Fig. 4B). Besides, these R9-conjugated macrocyclic peptides were also determined to exhibit low hemolytic toxicity towards mouse red blood cells (Fig. S8B†). It is worth mentioning that common macrocyclic peptides with the initiation codon reassigned to ClAc-^D^Tyr also yield a potent macrocyclic peptide, BAM-D1-R9 (Fig. 4B, S9 and Table S1†). However, we anticipate that fluorinated macrocyclic peptides might exhibit more drug-like characteristics, although the benefits of fluorine need to be further confirmed. Together, we have identified three R9-conjugated macrocyclic peptides as potent anti-Gram-negative bacterial agents using the RaPID system. This represents a novel approach for discovering anti-Gram-negative agents, distinct from traditional natural product sources.

**Fig. 5:**
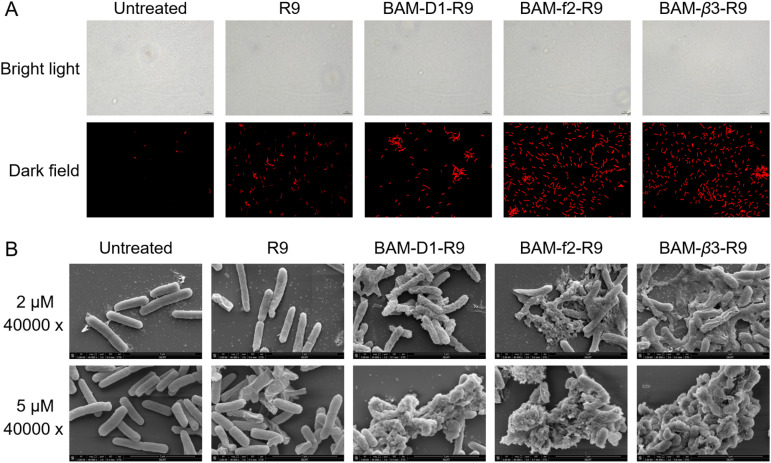
Fluorinated macrocyclic peptides disrupt the outer membrane and causes lysis of *E. coli*. (A) PI staining of *E. coli* W3110 in logarithmic-phase following a 15 min treatment with 5 μM fluorinated macrocyclic peptides. (B) Scanning electron microscopy analysis of *E. coli* W3110 in logarithmic-phase following a 4 h treatment with 2 μM and 5 μM fluorinated macrocyclic peptides.

**Fig. 6:**
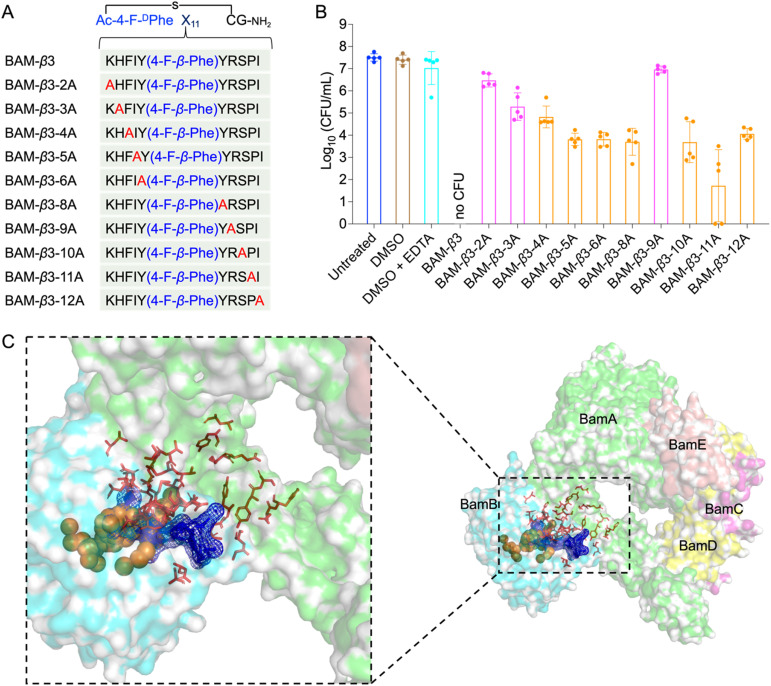
Alanine scanning mutagenesis of BAM-β3 against *E. coli* W3110 and prediction of the binding pocket of fluorinated macrocyclic peptides. (A) The sequences of alanine scanning mutagenesis of BAM-β3 (B) survival of *E. coli* W3110 cells in logarithmic-phase following a 15 min treatment with 50 μM BAM-β3 mutated macrocyclic peptides plus 1 mM EDTA. Each point represents the CFU value for one replicate, and a short line indicates the average CFU for each group. No CFU: no detectable CFU on the LB dish after 4 μL cell suspension was plated. (C) The predicted binding pocket of fluorinated macrocyclic peptides with BAM complex (PDB ID: 5AYW). BamA is illustrated as a green surface model, while BamB is depicted as a cyan surface model. Predictions made by the PointSite algorithm are represented by orange spheres, those by the PepNN algorithm are shown as red sticks, and predictions from the Fpocket algorithm are visualized as a blue mesh.

To elucidate the mechanisms of these peptides-mediated bacterial cell death, we first evaluated the cell membrane permeability of *E. coli* W3110 cells by using propidium iodide (PI), a widely used fluorescent probe for DNA staining in membrane-compromised cells (particularly dead cells).^52^ Our results showed weak red fluorescence in some R9-treated cells, whereas the BAM-D1-R9, BAM-f2-R9, and BAM-β3-R9 treated cells displayed notable red fluorescence intensity, suggesting that these peptides induced certain cell membrane damage (Fig. 5A). Subsequently, we conducted protein leakage assay. Post-treatment with BAM-D1-R9, BAM-f2-R9, and BAM-β3-R9, followed by centrifugation, revealed no significant bacterial pellet, and the protein bands were less intense compared to the R9-treated sample (Fig. S10†), indicating the cytoplasmic membrane of *E. coli* W3110 cells might be damaged by these peptides, leading to partial cellular protein leakage. Furthermore, we utilized scanning electron microscopy to observe the morphological changes in *E. coli* W3110 after 4 h treatment with peptides at two concentrations (2 μM and 5 μM). The morphology of untreated cells appeared normal, and the R9-treated cells showed slight shrinkage and partial rupture. In contrast, the BAM-D1-R9, BAM-f2-R9, and BAM-β3-R9-treated cells exhibited severe damage in the cell shape and even lysis, resulting in cell–cell aggregates (Fig. 5B and S11†). Together, these findings suggest that the macrocyclic peptides could severely disrupt the cell membranes of *E. coli*, conceivably by interfering the function of BAM complex in the folding and insertion of outer membrane proteins, which finally lead to severe cell membrane damage as we previously reported.^53^ Additionally, we conducted alanine scanning mutagenesis of BAM-β3 against *E. coli* W3110, confirming the significance of residues 2K, 3H, and 9R for its antibacterial activity (Fig. 6A and B).

Given the potent antimicrobial activity of macrocyclic peptides, we employed Fpocket, PointSite, and PepNN models to predict the potential binding sites of BAM-D1, BAM-f2, and BAM-β3 on the BAM complex.^54–58^ Firstly, the Fpocket algorithm was employed, which is a rapid method for pocket prediction based on the three-dimensional structure of proteins. Next, we used PointSite, which also utilizes the structure of protein to predict the binding sites, but incorporates machine learning for improved accuracy. Finally, the PepNN algorithm, designed for peptide binding site identification, was applied. Given limitation of PepNN with non-canonical amino acids or residues, macrocyclic peptides were segmented at non-canonical sites into multiple fragments, each containing solely canonical amino acids. The average scores of these fragments were computed to determine final predictive outcomes. According to the prediction results from the above three models, the binding pocket is likely situated near the junction between BamA and BamB, a crucial site for the correct insertion of β-barrel proteins (Fig. 6C and S12†). However, further experiments are necessary to confirm this finding definitively.

## Conclusions

In summary, we have achieved the ribosomal translation of a range of fluorinated non-canonical amino acids utilizing the FIT system, including mono-to tri-fluorinated variants, as well as fluorinated l-amino acids, d-amino acids, β-amino acids, *etc.* This enabled us to conduct *de novo* construction of fluorinated macrocyclic peptide libraries and screen for novel fluorinated macrocyclic peptides. We identified several promising fluorinated macrocyclic peptides with strong binding affinity to EphA2, as well as potential peptides demonstrating broad-spectrum activity against Gram-negative bacteria. This study not only expands the scope of ribosomally translatable fluorinated amino acids but also underscores the versatility of fluorinated macrocyclic peptides as potent therapeutic agents. While the benefits of fluorination were not confirmed, further exploration of these fluorinated macrocyclic peptides as therapeutic agents and investigation into the advantages of fluorination may offer additional insights.

## Author contributions

J. Wu carried out most parts of the experiments. Y. Wang and W. Cai performed some of the experiments. D. Chen, X. Peng, H. Dong, J. Li, H. Liu, S. Shi, S. Tang, Z. Li, H. Sui, Y. Wang and C. Wu contributed to the supporting. Y. Yin, X. Fu and Z. Zhang supervised the study. Y. Yin, J. Wu and X. Fu wrote the manuscript. All authors participated in valuable discussion.

## Conflicts of interest

There are no conflicts to declare.

## Supplementary Material

SC-015-D4SC04061A-s001

## Data Availability

The data supporting this article are included in the ESI.†
