# Supplementary material for: Ribosomal translation of fluorinated non-canonical amino acids for de novo biologically active fluorinated macrocyclic peptides
Source: Chem Sci. 2024 Aug 2;15(34):13889–98. doi: 10.1039/d4sc04061a (PMC11310889; doi:10.1039/d4sc04061a)
Supplement: SC-015-D4SC04061A-s001 [file SC-015-D4SC04061A-s001.pdf]

Supplementary Information (SI) for Chemical Science.
This journal is © The Royal Society of Chemistry 2024

Supporting information

Ribosomal Translation of Fluorinated Non-Canonical Amino Acids for *De Novo* Biologically Active Fluorinated Macrocyclic Peptides

Junjie Wu, Yuchan Wang, Wenfeng Cai, Danyan Chen, Xiangda Peng, Huilei Dong, Jinjing Li, Hongtan Liu, Shuting Shi, Sen Tang, Zhifeng Li, Haiyan Sui, Yan Wang, Chuanliu Wu, Youming Zhang*, Xinmiao Fu*, and Yizhen Yin*

*Corresponding authors: zhangyouming@sdu.edu.cn, xmfu@fjnu.edu.cn and yizhenyin.1987@sdu.edu.cn

Table of contents

1. Materials and methods

1.1 Abbreviations……………………………………………………………………….S3

1.2 Materials…………………………….……………...……………………………S3

1.3 Procedure for synthesis of activated fluorinated amino acids…..…………….…………S4

1.4 Aminoacylation of tRNA and microhelix RNA…………………………..…………S10

1.5 Ribosomal translation of fluorinated amino acids using FIT system.…………......…S11

1.6 Expression and purification of the BAM complex………………………....……..…S11

1.7 RaPID selection of macrocyclic peptides against the BAM complex……………..…......S11

1.8 General procedures for RaPID selection of fluorinated macrocyclic peptides against BAM complex and EphA2………………..…………………………………………….…….S12

1.9 General procedures for solid-phase synthesis of selected peptides…………..…...….S13

1.10 Binding kinetics analysis by SPR…………………………..………………..…..….S13

1.11 Preliminary evaluation of the antibacterial activity of macrocyclic peptides against E. coli W3110…………………………………………………………………………………S14

1.12 Hemolysis assay……………………..………………………...……......……..S14

1.13 Evaluation of R9-conjugated macrocyclic peptides against Gram-negative pathogens..S15

1.14 Propidium Iodide (PI) assay……………………………..……………..…..….S15

1.15 Protein leakage assay…………………………...…………..……………..…..….S16

1.16 Scanning electron microscopy (SEM)……………………..……………..…..….S16

1.17 Prediction of binding sites between peptides and the BAM complex……….…...….S17

2. Supplementary Data

2.1 Acid denaturing PAGE analysis of aminoacyl-microhelix RNA (Figure S1 and S2)……………………………………………………………………...…….……….S18

2.2 RaPID selections against EphA2 using fluorinated macrocyclic peptides library (Figure S3)……………………………………………………………………………...… S19

2.3 SPR results of the Ep-F2-M2 (Figure S4)……………………..……….………...… S19

2.4 RaPID selections against the BAM complex using fluorinated macrocyclic peptides library (Figure S5 and S6)………………………………………………………………...… S20

2.5 Results of macrocyclic peptides and fluorinated macrocyclic peptide against *E. coli* W3110 (Figure S7)………………..……………...…...…...…………………………………S21

2.6 Hemolysis activity of macrocyclic peptides and CPP-conjugated peptides toward RBCs

(Figure S8)…..………………………………….…………………...………………..….S21

2.7 SPR sensorgrams of macrocyclic peptides targeting BAM complex (Figure S9)…........ S22

2.8 Binding properties of macrocyclic peptides to BAM complex. (Table S1)…..……..….S22

2.9 The result of protein leakage assay (Figure S10)……………………………….....…. S23

2.10 The result of dot board corresponding to SEM (Figure S11)…………..…..…………. S23

2.11 The predicted binding pocket of fluorinated macrocyclic peptides with BAM complex(Figure S12)………………………………………………………………..S24

2.12 The sequence of peptide fragments used to predict the interaction sites of BAM-D1, BAM-f2 and BAM-β3 with BAM complex (Table S2)…..………….………………..….S24

2.13 RaPID selections against the BAM complex using macrocyclic peptides library (Figure S13).…………..……………………………………………………..……….…. S25

2.14 Analysis of sequencing results (Figure S14—17)..……………..……………..………. S26

2.15 NMR spectra of activated fluorinated amino acids.……..……………..….……..…...S30

2.16 The ESI-MS and HPLC figures of cyclic peptides by SPSS……………....……....….S48

Materials and methods

Abbreviations

The abbreviations used here are as follows: *A. baumannii* Ab6, *Acinetobacter baumannii* Ab6; AcOEt, ethyl acetate; BAM, *β*-barrel–assembly machinery; Boc, t-Butyloxy carbonyl; CME, cyanomethyl ester; CPP, cell penetrating peptide; DBE，3,5-dinitrobenzyl ester; DCM, dichloromethane; DDM, n-dodecyl-*β*-D-maltopyranoside; DIC, *N, N*-diisopropylcarbodiimide; DMF, N, N-dimethylformamide; DMSO, dimethyl sulfoxide; *E. coli* W3110, *Escherichia coli* W3110; EDTA, ethylene diamine tetraacetic acid; EphA2, human ephrin type-A receptor 2; ESI-MS, electrospray ionization mass spectrometry; EtOH, ethanol; FIT, flexible in vitro translation system; Fmoc, 9-fluorenylmethyloxycarbonyl; h, hour; HCl, hydrogen chloride; HOBT, 1-hydroxybenzotriazole; HPLC, high performance liquid chromatography; IPTG, isopropyl *β*-D-thiogalactoside; *K. pneumonia* KP-D367, *Klebsiella pneumoniae* KP-D367; LB, Luria-Bertani; MALDI-TOF MS, matrix-assisted laser desorption ionization time-of-flight mass spectrometry; Met, methionine; min, minute; NaCl, sodium chloride; $NaHCO_3$, sodium hydrogen carbonate; Na_2SO4, sodium sulfate; NMR, nuclear magnetic resonance; *P. aeruginosa* PAO1, *Pseudomonas aeruginosa* PAO1; PAGE, polyacrylamide gel electrophoresis; PBS, phosphate buffered saline; PBST, phosphate buffered saline with Tween-20; RaPID, random nonstandard peptide integrated discovery; RBC, red blood cell; rpm, revolutions per minute; RT, room temperature; SPPS, solid phase peptide synthesis; SPR, surface plasmon resonance; *S. typhimurium* SL1344, *Salmonella typhimurium* SL1344; TFA, trifluoroacetic acid; Tris-HCl, tris（hydroxymethyl）aminomethane hydrochloride; Tyr, tyrosine.

Materials

4-F-^{L}PheGly, 4-F-^{D}PheGly, Boc-2F-^{L}Phe, Boc-4-F-*β*-Phe, Boc-2F-^{L}Pro, Fmoc-protected amino acids and 3,5-dinitrobenzene were purchased from Bide Pharmatech Co., Ltd. 4-F-^{L}Phe, 4-F-^{D}Phe, Boc-4-CF_3-^{L}Phe, *cis*-4-F-^{L}Pro, trifluoroacetic acid (TFA), chloroacetonitrile, di-tert-butyl decarbonate, triisopropylhydrosilane, 1,4-dioxane, dimethyl sulfoxide (DMSO) and n-dodecyl-*β*-D-maltopyranoside (DDM) were purchased from Shanghai Aladdin Biochemical Technology Co., Ltd. *trans*-4-F-^{L}Pro were purchased from Shanghai Haohong Scientific Co., Ltd. Primers were synthesized by Genscript Biotech Corporation (Nanjing, China). T4 RNA ligase, phusion® high-fidelity DNA polymerase and PURExpress kit were purchased from New England Biolabs (NEB). Dynabeads™ M-280 Streptavidin was purchased from Thermo Fisher Scientific. M-MLV reverse transcriptase RNase H Minus was purchased from Promega (Beijing) Biotech Co.,Ltd. Taq DNA polymerase and RNase inhibitor were purchased from ABclonal Biotech Co., Ltd. T7 RNA

polymerase was purchased from NovoBiotechnology Co., Ltd. Biotinylated human EphA2 protein was purchased from Acrobiosystems. Ampicillin, sodium salt and isopropyl *β*-D-thiogalactoside (IPTG) was purchased from Beijing Solarbio Science & Technology Co., Ltd. Amino acids used for ribosomal synthesis and Ni-NTA Sefinose™ Resin 6FF (Settled Resin) were purchased from Sangon Biotech (Shanghai)Co., Ltd. Triethylamine was purchased from Shanghai Macklin Biochemical Co., Ltd. *Escherichia coli* W3110, *Klebsiella pneumoniae* KP-D367, *Acinetobacter baumannii* Ab6, *Salmonella typhimurium* SL1344 and *Pseudomonas aeruginosa* PAO1 were obtained from Xinmiao Fu of Fujian Normal University.

Procedure for synthesis of activated fluorinated amino acids.

(1) Synthesis of ClAc-4-F-^{L}Phe-CME

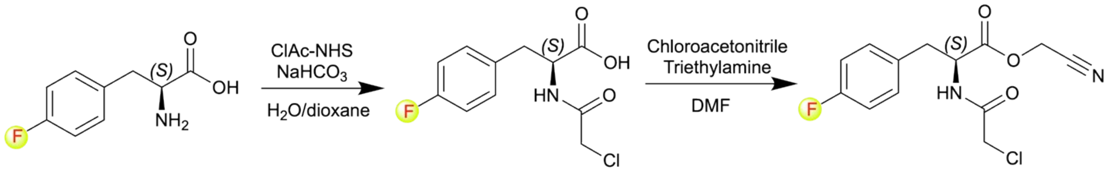

4-F-^{L}Phe ClAc-4-F-^{L}Phe ClAc-4-F-^{L}Phe-CME

A mixture of 4-F-^{L}Phe (100 mg, 0.55 mmol), N-(Chloroacetoxy)succinimide (118 mg, 0.61 mmol) and $NaHCO_3$ (138 mg, 1.64 mmol) in 2 mL of 50% 1,4-dioxane was stirred at RT for 3 h. The mixture was acidified at pH 2.0~3.0 by 1M HCl followed by extraction with AcOEt, and the combined organic layer was washed with brine thrice. The organic layer was dried over Na_2SO_4， concentrated under reduced pressure and vacuum drying for 1 hour. Then, the product (N-ClAc-4-F-^{L}Phe-OH) in 2 mL of DMF was mixed with triethylamine (84 μL, 0.61 mmol) and chloroacetonitrile (0.2 mL), and the reaction mixture was allowed to stir at RT for overnight. After the reaction is complete, AcOEt is added to the reaction solution, and the organic phase is washed three times with 1M HCl solution, three times with saturated $NaHCO_3$ and three times with brine. The recovered organic phase is dried with Na_2SO_4, followed by reduced pressure, and finally the desired product (ClAc-4-F-^{L}Phe-CME) is obtained. Finally, the resultant crude was purified by reverse-phase HPLC using aqueous solution/ acetonitrile as mobile phase to give the pure desired compound.

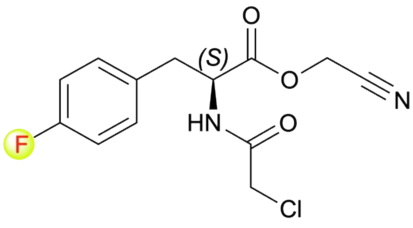

ClAc-4-F-^{L}Phe-CME

^{1}H NMR (600 MHz, DMSO-d_6) δ 8.81 (d, *J* = 7.5 Hz, 1H), 7.36 – 7.21 (m, 2H), 7.18 – 6.99 (m, 2H), 5.00 (s, 2H), 4.58 (ddd, *J* = 9.2, 7.5, 5.7 Hz, 1H), 4.06 (s, 2H), 3.09 (dd, *J* = 13.9, 5.7 Hz, 1H), 2.98 (dd, *J* = 13.9, 9.2 Hz, 1H). ^{13}C NMR (151 MHz, DMSO-d_6) δ 170.00, 166.09, 161.09 (d, *J* =242.6 Hz), 132.54 (d, *J* =2.8 Hz), 130.98 (d, *J* =8.1 Hz), 115.47, 115.02, 114.88, 53.43, 49.43, 41.94, 35.09. ^{19}F NMR (565 MHz, DMSO-d_6) δ -116.21 – -116.26 (m). ESI-MS (m/z) calculated for $C_{13}H_{12}ClFN_2O_3$ $[M+H]^+$: 299.06, 301.06, found: 298.68,

300.69.

(2) Synthesis of ClAc-4-F-^{L}Phe-CME

ClAc-4-F-^{D}Phe-CME was synthesized by the same procedures as ClAc-4-F-^{L}Phe-CME.

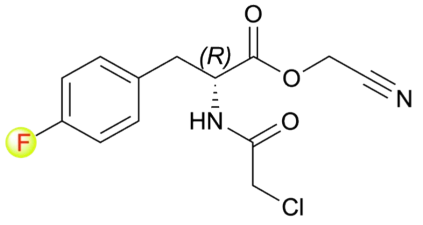

ClAc-4-F-^{D}Phe-CME

^{1}H NMR (600 MHz, DMSO-d$_6$) δ 8.81 (d, J = 7.5 Hz, 1H), 7.30 – 7.24 (m, 2H), 7.14 – 7.08 (m, 2H), 5.00 (s, 2H), 4.58 (ddd, J = 9.2, 7.5, 5.7 Hz, 1H), 4.06 (s, 2H), 3.09 (dd, J = 13.9, 5.7 Hz, 1H), 2.98 (dd, J = 13.9, 9.2 Hz, 1H). ^{13}C NMR (151 MHz, DMSO-d$_6$) δ 170.00, 166.10, 161.09 (d, J=242.6 Hz), 132.55 (d, J=3.3 Hz), 130.98 (d, J=8.1 Hz), 115.48, 115.02, 114.88, 53.44, 49.43, 41.94, 35.10. ^{19}F NMR (565 MHz, DMSO-d$_6$) δ -116.21 – -116.26 (m). ESI-MS (m/z) calculated for $C_{13}H_{12}ClFN_2O_3$ $[M+H]^+$: 299.06, 301.06, found: 298.60, 300.57.

(3) Synthesis of 4-F-^{L}Phe-CME

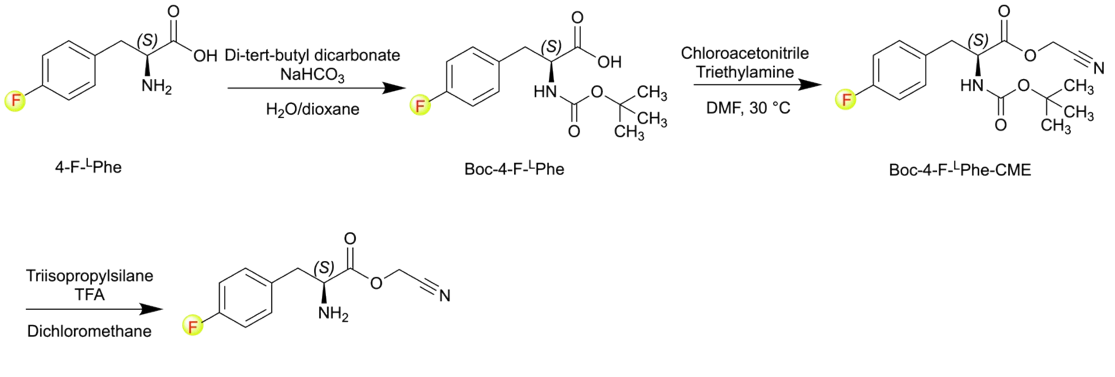

A mixture of 4-F-^{L}Phe (200 mg, 1.09 mmol), Di-tert-butyl dicarbonate (477 mg, 2.18 mmol) and $NaHCO_3$ (275 mg, 2.28 mmol) in 4 mL of 50% 1,4-dioxane was stirred at RT for 3 h. The mixture was acidified at pH 2.0~3.0 by 1M HCl followed by extraction with AcOEt, and the combined organic layer was washed with brine thrice. The organic layer was dried over Na_2SO_4， concentrated under reduced pressure and vacuum drying for 1 hour. Then, the product (Boc-4-F-^{L}Phe) in 4 mL of DMF was mixed with triethylamine (168 μL, 1.20 mmol) and chloroacetonitrile (0.4 mL), and the reaction mixture was allowed to stir at 30 °C for overnight. After the reaction is complete, AcOEt is added to the reaction solution, and the organic phase is washed three times with 1M HCl solution, three times with saturated $NaHCO_3$ and three times with brine. The recovered organic phase is dried with Na_2SO_4, followed by reduced pressure, and finally the desired product (Boc-4-F-^{L}Phe-CME) is obtained. The product (Boc-4-F-^{L}Phe-CME) in 3 mL of DCM was mixed with TFA (2 mL) and Triisopropylhydrosilane (671 μL, 3.28 mmol), and the reaction mixture was allowed to stir at RT

for 3 h. After the reaction, it is directly concentrated under reduced pressure, and dissolved with AcOEt to remove TFA. Finally, the resultant crude was purified by reverse-phase HPLC using 0.1% TFA aqueous solution/0.1% TFA containing acetonitrile as mobile phase to give the pure desired compound (4-F-^{L}Phe-CME).

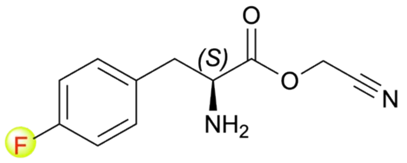

4-F-^{L}Phe-CME

^{1}H NMR (600 MHz, DMSO-d_6) δ 8.67 (s, 2H), 7.33 – 7.28 (m, 2H), 7.20 – 7.14 (m, 2H), 5.10 (d, *J* = 1.8 Hz, 2H), 4.45 (dd, *J* = 7.4, 6.3 Hz, 1H), 3.19 – 3.09 (m, 2H). ^{13}C NMR (151 MHz, DMSO-d_6) δ 168.09, 161.52 (d, *J* =243.3 Hz), 131.39 (d, *J* =8.2 Hz), 130.32 (d, *J* =3.1 Hz), 115.42, 115.28, 115.06, 52.87, 50.06, 34.81. ^{19}F NMR (565 MHz, DMSO-d_6) δ -115.32 – -115.37 (m). ESI-MS (m/z) calculated for $C_{11}H_{11}FN_2O_2$ $[M+H]^+$: 223.09, found: 222.30.

(4) Synthesis of 4-F-^{D}Phe-CME

4-F-^{D}Phe-CME was synthesized by the same procedures as 4-F-^{L}Phe-CME.

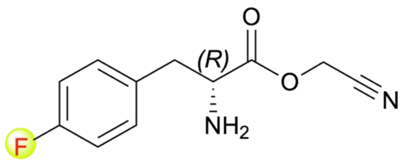

4-F-^{D}Phe-CME

^{1}H NMR (600 MHz, DMSO-d_6) δ 8.61 (s, 2H), 7.33 – 7.28 (m, 2H), 7.20 – 7.14 (m, 2H), 5.10 (d, *J* = 2.1 Hz, 2H), 4.48 – 4.43 (m, 1H), 3.13 (qd, *J* = 14.2, 6.8 Hz, 2H). ^{13}C NMR (151 MHz, DMSO-d_6) δ 168.10, 161.52 (d, *J* =243.5 Hz), 131.39 (d, *J* =8.2 Hz), 130.29 (d, *J* =3.1 Hz), 115.42, 115.28, 115.06, 52.85, 50.08, 34.80. ^{19}F NMR (565 MHz, DMSO-d_6) δ -115.30 – -115.35 (m). ESI-MS (m/z) calculated for $C_{11}H_{11}FN_2O_2$ $[M+H]^+$: 223.09, found: 222.53.

(5) Synthesis of 4-F-^{L}PheGly-CME

4-F-^{L}PheGly-CME was synthesized by the same procedures as 4-F-^{L}Phe-CME.

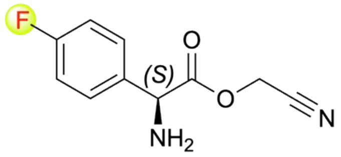

4-F-^{L}PheGly-CME

^{1}H NMR (600 MHz, DMSO-d_6) δ 8.75 (s, 2H), 7.61 – 7.48 (m, 2H), 7.41 – 7.26 (m, 2H), 5.48 (s, 1H), 5.19 – 5.11 (m, 2H). ^{13}C NMR (151 MHz, DMSO-d_6) δ 168.98, 163.65 (d, *J* =247.0 Hz), 131.68 (d, *J* =8.7 Hz), 129.66 (d, *J* =3.1 Hz), 117.12, 116.97, 116.17, 55.49, 51.49. ^{19}F NMR (565 MHz, DMSO-d_6) δ -111.46 – -111.51 (m). ESI-MS (m/z) calculated for $C_{10}H_9FN_2O_2$ $[M+H]^+$: 209.07, found: 208.11.

(6) Synthesis of 4-F-^{D}PheGly-CME

4-F-^{D}PheGly-CME was synthesized by the same procedures as 4-F-^{L}Phe-CME.

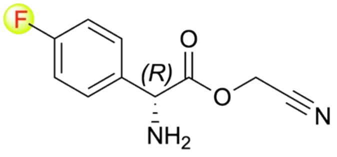

4-F-^{D}PheGly-CME

^{1}H NMR (600 MHz, DMSO-d_6) δ 9.20 (s, 2H), 7.62 – 7.55 (m,

2H), 7.39 – 7.29 (m, 2H), 5.53 (s, 1H), 5.20 – 5.12 (m, 2H). ^{13}C NMR (151 MHz, DMSO-d$_6$) δ 168.01, 163.23 (d, *J* =247.1 Hz), 131.43 (d, *J* =8.7 Hz), 128.59 (d, *J* =3.0 Hz), 116.63, 116.49, 115.66, 54.71, 51.07. ^{19}F NMR (565 MHz, DMSO-d$_6$) δ -111.33 – -111.38 (m). ESI-MS (m/z) calculated for $C_{10}H_9FN_2O_2$ [M+H]$^+$: 209.07, found: 208.16.

(7) Synthesis of 4-F-*β*-Phe-CME

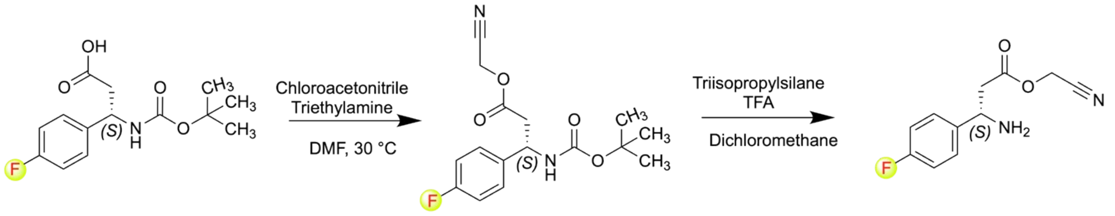

Boc-4-F-*β*-Phe Boc-4-F-*β*-Phe-CME 4-F-*β*-Phe-CME

A mixture of Boc-4-F-*β*-Phe (200 mg, 0.706 mmol), triethylamine (98 µL, 0.78 mmol) and chloroacetonitrile (0.4 mL) in 4 mL of DMF was stirred at 30 °C for overnight. After the reaction is complete, AcOEt is added to the reaction solution, and the organic phase is washed three times with 1M HCl solution, three times with saturated $NaHCO_3$ and three times with brine. The recovered organic phase is dried with Na_2SO_4, followed by reduced pressure, and finally the desired product (Boc-4-F-*β*-Phe-CME) is obtained. The product (Boc-4-F-*β*-Phe-CME) in 3 mL of DCM was mixed with TFA (2 mL) and Triisopropylhydrosilane (434 µL, 2.12 mmol), and the reaction mixture was allowed to stir at RT for 3 h. After the reaction, it is directly concentrated under reduced pressure, and dissolved with AcOEt to remove TFA. Finally, the resultant crude was purified by reverse-phase HPLC using 0.1% TFA aqueous solution/0.1% TFA containing acetonitrile as mobile phase to give the pure desired compound (4-F-*β*-Phe-CME).

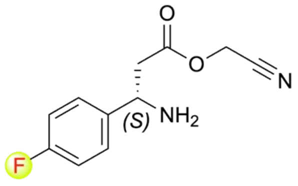

4-F-*β*-Phe-CME

^{1}H NMR (600 MHz, DMSO-d$_6$) δ 8.47 (s, 2H), 7.62 – 7.50 (m, 2H), 7.33 – 7.24 (m, 2H), 4.94 (d, *J* = 1.4 Hz, 2H), 4.70 (dd, *J* = 8.1, 6.4 Hz, 1H), 3.23 – 3.08 (m, 2H). ^{13}C NMR (151 MHz, DMSO-d$_6$) δ 169.18, 163.25 (d, *J* =245.7 Hz), 133.57 (d, *J* =3.2 Hz), 130.87 (d, *J* =8.6 Hz), 116.69, 116.55, 116.54, 50.80, 50.34, 38.82. ^{19}F NMR (565 MHz, DMSO-d$_6$) δ -112.69 – -112.74 (m). ESI-MS (m/z) calculated for $C_{11}H_{11}FN_2O_2$ [M+H]$^+$: 223.09, found: 222.33.

(8) Synthesis of 2F-^{L}Phe-CME

2F-^{L}Phe-CME was synthesized by the same procedures as 4-F-*β*-Phe-CME.

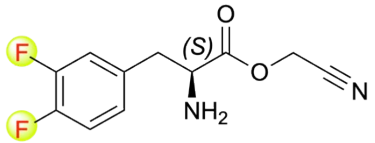

2F-^{L}Phe-CME

^{1}H NMR (600 MHz, DMSO-d$_6$) δ 8.73 (s, 2H), 7.43 – 7.38 (m, 2H), 7.15 – 7.12 (m, 1H), 5.12 (s, 2H), 4.49 (t, *J* = 6.8 Hz,

1H), 3.20 – 3.15 (m, 2H). ^{13}C NMR (151 MHz, DMSO-d$_6$) δ 168.49, 150.38 (dd, *J* =12.5 Hz), 148.76 (dd, *J* =12.9 Hz), 132.53 (dd, *J* =3.5 Hz), 127.05 (dd, *J* =3.3 Hz), 119.22 (d, *J* =17.3 Hz), 118.07 (d, *J* =16.9 Hz), 115.67, 53.11, 50.71, 35.05. ^{19}F NMR (565 MHz, DMSO-d$_6$) δ -138.50 – -138.57 (m), -140.80 – -140.88 (m). ESI-MS (m/z) calculated for $C_{11}H_{10}F_2N_2O_2$ $[M+H]^+$: 241.08, found: 240.62.

(9) Synthesis of 4-CF_3-^{L}Phe-CME

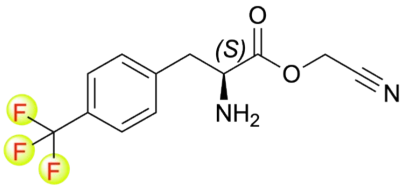

4-CF_3-^{L}Phe-CME

4-CF_3-^{L}Phe-CME was synthesized by the same procedures as 4-F-*β*-Phe-CME. ^{1}H NMR (600 MHz, DMSO-d$_6$) δ 8.89 – 8.66 (m, 2H), 7.71 (dd, *J* = 8.1, 6.0 Hz, 2H), 7.53 (d, *J* = 7.9 Hz, 2H), 5.11 (d, *J* = 1.1 Hz, 2H), 4.64 – 4.47 (m, 1H), 3.27 (qd, *J* = 14.3, 6.9 Hz, 2H). ^{13}C NMR (151 MHz, DMSO-d$_6$) δ 168.50, 139.80, 130.93, 130.90, 128.55 (q, *J* =31.7 Hz), 125.93 (q, *J* =3.5 Hz), 124.75 (q, *J* =272.6 Hz), 115.63, 53.29, 50.73, 35.77. ^{19}F NMR (565 MHz, DMSO-d$_6$) δ -60.95. ESI-MS (m/z) calculated for $C_{12}H_{11}F_3N_2O_2$ $[M+H]^+$: 273.09, found: 273.15.

(10) Synthesis of 2F-^{L}Pro-DBE

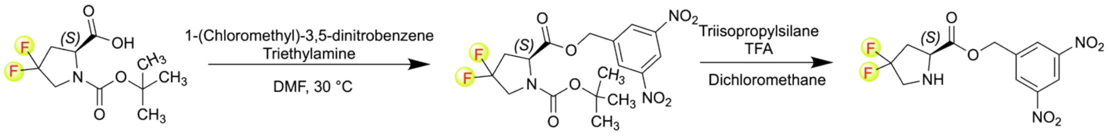

Boc-2F-^{L}Pro Boc-2F-^{L}Pro-DBE 2F-^{L}Pro-DBE

2F-^{L}Pro, *cis*-4-F-^{L}Pro and *trans*-4-F-^{L}Pro were preactivated as the 3,5-dinitrobenzyl ester forms. A mixture of Boc-2F-^{L}Pro (200 mg, 0.8 mmol), triethylamine (111 μL, 0.88 mmol) and 1-(Chloromethyl)-3,5-dinitrobenzene (345 mg, 1.6 mmol) in 4 mL of DMF was stirred at 30 °C for overnight. After the reaction is complete, AcOEt is added to the reaction solution, and the organic phase is washed three times with 1M HCl solution, three times with saturated $NaHCO_3$ and three times with brine. The recovered organic phase is dried with Na_2SO_4, followed by reduced pressure, and finally the desired product (Boc-2F-^{L}Pro-DBE) is obtained. The product (Boc-2F-^{L}Pro-DBE) in 3 mL of DCM was mixed with TFA (2 mL) and Triisopropylhydrosilane (489 μL, 2.39 mmol), and the reaction mixture was allowed to stir at RT for 3 h. After the reaction, it is directly concentrated under reduced pressure, and dissolved with AcOEt to remove TFA. Finally, the resultant crude was purified by reverse-phase HPLC using 0.1% TFA aqueous solution/0.1% TFA containing acetonitrile as mobile phase to give the pure desired compound (2F-^{L}Pro-DBE).

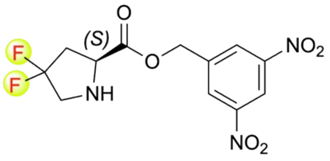

2F-^{L}Pro-DBE

^{1}H NMR (600 MHz, DMSO-d$_6$) δ 8.83 (t, *J* = 2.1 Hz, 1H), 8.77 (d, *J* = 2.0 Hz, 2H), 5.52 (s, 2H), 4.90 (t, *J* = 8.6 Hz, 1H), 3.82 –

3.68 (m, 2H), 2.97 – 2.76 (m, 2H). ^{13}C NMR (151 MHz, DMSO-d$_6$) δ 166.95, 147.98, 139.27, 128.52, 127.23 (t, J =248.6 Hz), 118.38, 65.32, 56.67 (t, J =3.8 Hz), 50.73 (t, J =33.4 Hz), 36.03 (t, J =25.6 Hz). ^{19}F NMR (565 MHz, DMSO-d$_6$) δ -95.48 – -95.57 (m). ESI-MS (m/z) calculated for $C_{12}H_{11}F_2N_3O_6$ [M+H]$^+$: 332.07, found: 332.03.

(11) Synthesis of *cis*-4-F-^{L}Pro-DBE

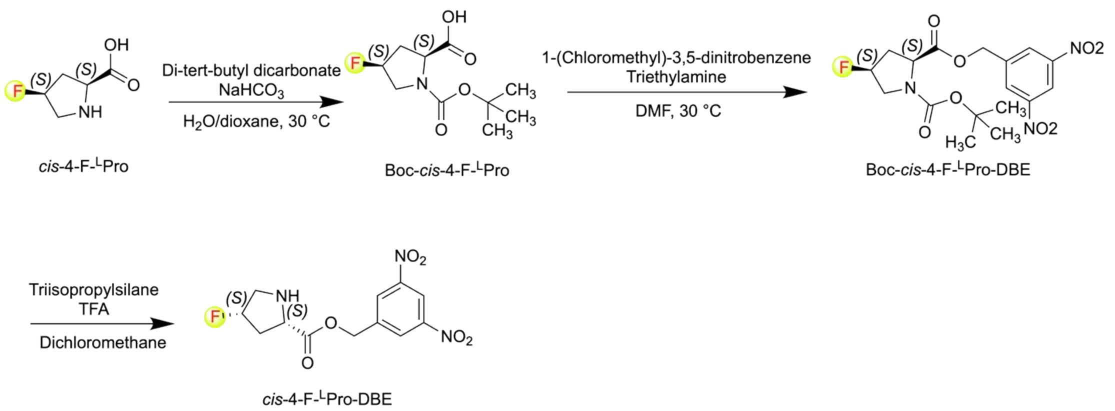

A mixture of *cis*-4-F-^{L}Pro (150 mg, 1.13 mmol), Di-tert-butyl dicarbonate (492 mg, 2.25 mmol) and $NaHCO_3$ (284 mg, 3.38 mmol) in 3 mL of 50% 1,4-dioxane was stirred at 30°C for 6 h. The mixture was acidified at pH 2.0~3.0 by 1M HCl followed by extraction with AcOEt, and the combined organic layer was washed with brine thrice. The organic layer was dried over Na_2SO_4， concentrated under reduced pressure and vacuum drying for 1 hour. Then, the product (Boc-*cis*-4-F-^{L}Pro) and 1-(Chloromethyl)-3,5-dinitrobenzene (488 mg, 2.25 mmol) in 3 mL of DMF was mixed with triethylamine (172 μL, 1.24 mmol), and the reaction mixture was allowed to stir at 30 °C for overnight. After the reaction is complete, AcOEt is added to the reaction solution, and the organic phase is washed three times with 1M HCl solution, three times with saturated $NaHCO_3$ and three times with brine. The recovered organic phase is dried with Na_2SO_4, followed by reduced pressure, and finally the desired product (Boc-*cis*-4-F-^{L}Pro) is obtained. The product (Boc-*cis*-4-F-^{L}Pro) in 3 mL of DCM was mixed with TFA (2 mL) and Triisopropylhydrosilane (671 μL, 3.28 mmol), and the reaction mixture was allowed to stir at RT for 3 h. After the reaction, it is directly concentrated under reduced pressure, and dissolved with AcOEt to remove TFA. Finally, the resultant crude was purified by reverse-phase HPLC using 0.1% TFA aqueous solution/0.1% TFA containing acetonitrile as mobile phase to give the pure desired compound (*cis*-4-F-^{L}Pro-DBE).

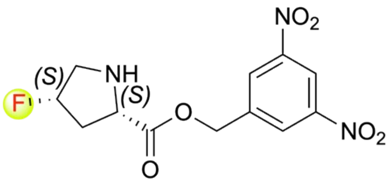

cis-4-F-^{L}Pro-DBE

^{1}H NMR (600 MHz, DMSO-d$_6$) δ 8.83 (t, J = 2.1 Hz, 1H), 8.75 (d, J = 2.1 Hz, 2H), 5.54 – 5.42 (m, 3H), 4.82 (dd, J = 10.1, 3.8 Hz, 1H), 3.67 – 3.58 (m, 1H), 3.56 – 3.45 (m, 1H), 2.70 – 2.52 (m, 2H). ^{13}C NMR (151 MHz, DMSO-d$_6$) δ 168.27, 147.98, 139.37, 128.50, 118.37, 91.88 (d, J =174.6

Hz), 65.35, 57.58, 51.70 (d, *J* =23.5 Hz), 35.28 (d, *J* =21.6 Hz). ^{19}F NMR (565 MHz, DMSO-d_6) δ -173.03 – -173.33 (m). ESI-MS (m/z) calculated for $C_{12}H_{12}FN_3O_6$ $[M+H]^+$: 314.08, found: 314.18.

(12) Synthesis of *trans*-4-F-^{L}Pro-DBE

trans-4-F-^{L}Pro-DBE was synthesized by the same procedures as *cis*-4-F-^{L}Pro.

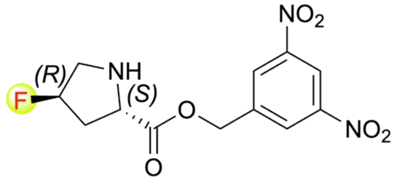

trans-4-F-^{L}Pro-DBE

^{1}H NMR (600 MHz, DMSO-d_6) δ 8.83 (t, *J* = 2.1 Hz, 1H), 8.78 (d, *J* = 2.1 Hz, 2H), 5.57 – 5.44 (m, 3H), 4.77 (dd, *J* = 11.0, 7.5 Hz, 1H), 3.65 – 3.52 (m, 2H), 2.68 – 2.57 (m, 1H), 2.49 – 2.37 (m, 1H). ^{13}C NMR (151 MHz, DMSO-d_6) δ 167.55, 148.01, 139.38, 128.55, 118.36, 92.04 (d, *J* =175.4 Hz), 65.23, 57.35, 51.55 (d, *J* =23.6 Hz), 34.78 (d, *J* =21.3 Hz). ^{19}F NMR (565 MHz, DMSO-d_6) δ -174.52 – -174.82 (m). ESI-MS (m/z) calculated for $C_{12}H_{12}FN_3O_6$ $[M+H]^+$: 314.08, found: 314.17.

Aminoacylation of tRNA and microhelix RNA

tRNA$^{fMet}_{CAU}$, tRNA$^{Asn-E2}_{CAU}$，tRNA$^{Pro1E2}_{CAU}$, microhelix RNA and flexizyme (eFx and dFx) used in this study were synthesized using the same procedure as previously described.[1-4] For the reaction of aminoacylation, the mixtures was allowed to incubate on ice for the optimized time, which containing 50 mM HEPES–KOH (pH 7.5), 600 mM $MgCl_2$, 20% DMSO, 25 μM dFx or eFx, 25 μM tRNA, and 5 mM activated amino acid. eFx was used for ClAc-4-F-^{L}Phe-CME, ClAc-4-F-^{D}Phe-CME, 4-F-^{L}Phe-CME, 4-F-^{D}Phe-CME, 4-F-^{L}PheGly-CME, 4-F-^{D}PheGly-CME, 2F-^{L}Phe-CME, 4-CF_3-^{L}Phe-CME and 4-F-*β*-Phe-CME. dFx was used for 2F-^{L}Pro-DBE, *cis*-4-F-^{L}Pro-DBE and *trans*-4-F-^{L}Pro-DBE. The reaction time was 12 h for 4-F-^{L}PheGly-CME, 4-F-^{D}PheGly-CME and 2F-^{L}Pro-DBE, and 2 h for the other fluorinated activated amino acids. After the reaction, the aminoacyl-tRNA or aminoacyl-microhelix RNA was recovered by EtOH precipitation. The aminoacyl-tRNA or aminoacyl-microhelix RNA pellet was rinsed twice with 70% EtOH containing 0.1 M NaOAc (pH 5.2), and once with 70% EtOH. Acid-PAGE analysis of aminoacyl-microhelix RNA with activated fluorinated amino acids. ClAc-^{L}Tyr-CME was used as a positive control, DMSO was used as a negative control. Aminoacylation yields were calculated in light of the band intensity of Microhelix-AA and unreacted microhelix RNA, presented as Microhelix-AA/(Microhelix-AA + Microhelix).

Ribosomal translation of fluorinated amino acids using FIT system

Ribosomal translation was carried out in Met omitting FIT system as previously described.[5] The rP1-rP2 reaction mixtures was performed at 5 μL scale with 1 μM mRNA, 50 μM ClAc-4-F-^{L}Phe-tRNA$^{fMet}_{CAU}$ or ClAc-4-F-^{D}Phe-tRNA$^{fMet}_{CAU}$ and incubated at 37 °C for 120 min. The rP3-rP12 reaction mixtures was performed at 5 μL scale with 1 μM mRNA, 50 μM ClAc-4-F-^{L}Phe-

tRNA$^{fMet}_{CAU}$, 50 μM FAA-tRNA$^{Asn-E2}_{CAU}$ or FAA-tRNA$^{Pro1E2}_{CAU}$ and incubated at 37 °C for 120 min. The translation mixture was analyzed by MALDI-TOF MS.

Expression and purification of the BAM complex

The *E. coli* BL21(DE3) cells containing all five Bam proteins (BamA, B, C, D, and E) was obtained from Xinmiao Fu of Fujian Normal University and used for expression and purification. The BL21(DE3) cells plated onto LB-ampicillin agar plates and incubated overnight at 37 °C. A 20 mL culture incubated overnight at 37°C was prepared from a single colony in LB medium supplemented with 100 mg/L of ampicillin. The strain was inoculated into fresh LB liquid medium according to the inoculation ratio of 1:100 and incubated at 37 ℃ for about 4 hours until the OD_{600} reached about 1.0. The temperature of the incubator was set to 20 ℃ and then cultured for 1 hour, and 200 uM IPTG was added to induce overnight. First of all, the induced cells were collected by freezing centrifuge 3000 rpm for 30 min, and the collected cells were resuspended with Buffer 1 (20 mM Tris-HCl, pH 8.0, 150 mM NaCl), and then crushed low temperature and pressure with a high pressure crusher. The broken cells were centrifuged 1 hour at 4 °C for18000 rpm by high speed centrifuge. After centrifugation at 4 °C 18000 rpm for 1 hour, and the supernatant was discarded and the cell membrane was collected. The collected cell membranes were resuscitated with Buffer 2 (20 mM Tris-HCl, pH 8.0, 300 mM NaCl, 1% DDM) and then dissolved at 4 °C for 2 hours. After dissolving the membrane, the supernatant was collected by high speed centrifuge at 18,000 rpm. The 20 mM imidazole was added to the supernatant and incubated with a 2 mL Ni-NTA sefinose™ resin column for 1 hour. After incubation, 10 column volumes were washed with Buffer 3 (20 mM Tris-HCl, pH 8.0, 300 mM NaCl, 0.025%DDM) containing 30 mM and 50 mM imidazole respectively. Then, the protein was eluted with Buffer 3 containing 200 mM imidazole, and the eluted protein was collected. Finally, using the 20-fold molar excess of EZ-Link™ Sulfo-NHS-LC-LC-Biotin for BAM complex, and incubate reaction on ice for 2 hours. After the protein labeling is completed, dialysis is used to purify the labeled protein to remove the remaining unreacted and hydrolyzed biotin reagents.

RaPID selection of macrocyclic peptides against BAM complex

Before the first round of screening, the mRNA template library (AUG-$(NNK)_{8\text{-}12}$-UGC-$(GGC\text{-}AGC)_3$-UAG) was ligated with puromycin linker (DNA-PEG-CC-Pu, 5'-pCTCCCGCCCCCCGTCC-PEG linker-CC-puromycin-3') with T4 ligase to obtain the mRNA-Pu library. Then, the 1.2 μM mRNA-Pu library was incubated for 120 minutes at 37 ℃ in RF1 and Met omitted FIT system containing 50 μM ClAc-^{D}Tyr-tRNA$^{fMet}_{CAU}$. After the reaction, the mixture were incubated at 25 ℃ for 15 minutes, then 5 μL of 100 mM EDTA (pH 8.0) was added to continue incubating at 37 ℃ for 30 minutes. The mixture was then mixed with a reverse transcription solution include M-MLV reverse transcriptase RNase H negative group，RNase inhibitor, 25 mM Tris-HCl

(pH 8.3), 15 mM $Mg(OAc)_2$, 10 mM KOH, 0.25 mM dNTPs, 2 μM CGS3an13.R39 (5'-TTTCC GCCCC CCGTC CTAGC TGCCG CTGCC GCTGC CGCA-3') and incubated at 42 °C for 60 minutes. The obtained mixture was then mixed with the blocking solution (20 mM phosphate pH 7.4, 275 mM NaCl, 5.4 mM KCl, 0.1% Tween 20 and 0.2% acetyl BSA) at 1:1 and incubated at 4 °C with BAM complex Dynabeads™ M-280 Streptavidin for 60 minutes (called positive selection). Then, discard the supernatant, wash the beads with PBST (10 mM Phosphate pH 7.4, 137 mM NaCl, 2.7 mM KCl, 0.05% Tween 20) for three times, then add PCR mix containing 0.25 μM T7g10M.F46 (5'-TAATA CGACT CACTA TAGGG TTAAC TTTAA GAAGG AGATA TACAT A-3'), 0.25 μM CGS3an13.R22, heat it at 95°C for 10 minutes, and elute to get cDNAs. The cDNAs was detected by QuantStudio™ real-time PCR system (Applied Biosystems), then PCR was recovered by Taq DNA polymerase and transcribed *in vitro* to construct a mRNA library rich in BAM complex binding sequences to prepare for the next round of screening. Starting with the second round of screening, six more negative selections were added before positive selections to remove unwanted beads binders. Finally, the cDNAs recovered in the sixth round was deep sequencing using NovaSeq 6000 system (Ilumina).

General procedures for RaPID selection of fluorinated macrocyclic peptides against BAM complex and EphA2

Before the first round of screening, the mRNA template library (AUG-$(NNK)_{5\text{-}7}$-AUG-$(NNK)_5$-UGC-$(GGC\text{-}AGC)_3$-UAG) was ligated with puromycin linker (DNA-PEG-CC-Pu, 5'-pCTCCCGCCCCCCGTCC-PEG linker-CC-puromycin-3') with T4 ligase to obtain the mRNA-Pu library. Then, the 1.2 μM mRNA-Pu library was incubated for 120 minutes at 37 °C in RF1 and Met omitted FIT system containing 50 μM ClAc-4-F-^{D}Phe-tRNA$^{fMet}_{CAU}$ and 4-F-^{D}Phe-tRNA$^{Asn\text{-}E2}_{CAU}$ (or 2F-^{L}Phe-tRNA$^{Asn\text{-}E2}_{CAU}$, 4-F-*β*-Phe-tRNA$^{Asn\text{-}E2}_{CAU}$). After the reaction, the mixture were incubated at 25 °C for 15 minutes, then 5 μL of 100 mM EDTA (pH 8.0) was added to continue incubating at 37 °C for 30 minutes. The mixture was then mixed with a reverse transcription solution include M-MLV reverse transcriptase RNase H negative group，RNase inhibitor, 25 mM Tris-HCl (pH 8.3), 15 mM $Mg(OAc)_2$, 10 mM KOH, 0.25 mM dNTPs, 2 μM CGS3an13.R39 (5'-TTTCC GCCCC CCGTC CTAGC TGCCG CTGCC GCTGC CGCA-3') and incubated at 42 °C for 60 minutes. The obtained mixture was then mixed with the blocking solution (20 mM phosphate pH 7.4, 275 mM NaCl, 5.4 mM KCl, 0.1% Tween 20 and 0.2% acetyl BSA) at 1:1 and incubated at 4 °C with BAM complex(or EphA2) Dynabeads™ M-280 Streptavidin for 60 minutes (called positive selection). Then, discard the supernatant, wash the beads with PBST (10 mM Phosphate pH 7.4, 137 mM NaCl, 2.7 mM KCl, 0.05% Tween 20) for three times, then add PCR mix containing 0.25 μM T7g10M.F46 (5'-TAATA CGACT CACTA TAGGG TTAAC TTTAA GAAGG AGATA TACAT A-3'), 0.25 μM CGS3an13.R22, heat it at 95 °C for 10 minutes, and elute to get cDNAs.

The cDNAs was detected by QuantStudio™ real-time PCR system (Applied Biosystems), then PCR was recovered by phusion® high-fidelity DNA polymerase and transcribed *in vitro* to construct a mRNA library rich in BAM complex(or EphA2) binding sequences to prepare for the next round of screening. Starting with the second round of screening, six more negative selections were added before positive selections to remove unwanted beads binders. Finally, the cDNAs recovered in the sixth(or fifth) round was deep sequencing using NovaSeq 6000 system (Ilumina).

General procedures for solid-phase synthesis of selected peptides

In this study, macrocyclic peptides and fluorinated macrocyclic peptides were synthesized by standard Fmoc solid phase peptide synthesis (SPPS). Firstly, a mixture (0.3 mmol Fmoc-Gly-OH and 0.3 mmol HOBT dissolved in 8 mL DMF and 0.5 mL DIC) was prepared, and 0.5 g Rink Amide MBHA resin was added to the mixture, and then reacted under nitrogen protection for 1.5 h. The resin was filtered and washed at least three times with DMF and DCM, and a deprotective agent (DMF solution containing 20% piperidine) was added to remove the Fmoc protective group. Then a new mixture (0.9 mmol Fmoc-AA-OH and 0.9 mmol HOBT dissolved in 10 mL DMF and 1 mL DIC) was prepared and condensation reaction was carried out under nitrogen protection for 1 hour. By repeating the steps of deprotection and condensation reaction, the desired peptide was finally obtained, and the bromoacetyl group was connected to the *N*-terminal free α-amino group of the resin peptide. Then the peptides were broken down from the resin, precipitated with ether, then dissolved again with DMSO, and then incubated for 1 hour after adjusting the pH value of the solution to 8.0, the macrocyclic peptides and fluorinated macrocyclic peptides containing thioether bonds were obtained. Finally, the pH value was adjusted to 3～4 with TFA and purified by reversed-phase HPLC. The mobile phase was 0.1% TFA aqueous solution / acetonitrile containing 0.1% TFA. The purified macrocyclic peptides and fluorinated macrocyclic peptides were confirmed by electrospray ionization mass spectrometry (ESI-MS).

Binding kinetics analysis by SPR

The binding kinetics between the selected peptides and EphA2 were tested by SPR using a Biacore T200 instrument (GE Healthcare) at 25 °C. In this study, the running buffer was HBS-EP+ buffer (10 mM HEPES-KOH (pH 7.4), 150 mM NaCl, 50 μM EDTA, 0.05% Tween 20) containing 0.1% DMSO. Biotin capture kit (GE Healthcare) was used to fix human EphA2 on the surface of Biacore CAP chip with a density of 1050 RU. Five different concentrations of peptides were injected at the flow rate of 30 μL/min, and their kinetic constants were determined. The binding kinetic was quantified by single cycle kinetic method. Finally, the binding sensorgrams was analyzed by Biacore evaluation software, and the standard 1:1 model was used for fitting.

The binding kinetics between the selected peptides and BAM complex were tested by SPR using

a Biacore T200 instrument (GE Healthcare) at 25 °C. In this study, the running buffer was HBS-EP+ buffer (10 mM HEPES-KOH (pH 7.4), 150 mM NaCl, 50 μM EDTA, 0.05% Tween 20) containing 0.1% DMSO. The Series S Sensor Chip CM5 was used to fix BAM complex on the surface of Biacore CM5 chip with a density of 9500 RU. The binding mode was multi-cycle kinetics (one sample concentration one cycle). Finally, the binding sensorgrams was analyzed by Biacore evaluation software, and the standard 1:1 model was used for fitting.

Preliminary evaluation of the antibacterial activity of macrocyclic peptides against *E. coli* W3110

Remove the previously preserved *E. coli* W3110 cells from the -80 °C refrigerator, scrape a little, inoculate it into 1 mL LB liquid medium, and culture overnight in 37°C shaker. The strain was inoculated into fresh LB liquid medium according to the inoculation ratio of 1:100 and incubated at 37°C until the OD_{600} reached about 0.47～0.53. First, 100 μL *E. coli* W3110 cells was taken, then centrifuged for 2 minutes at 13000 rpm, then the supernatant was absorbed and discarded, and then the cells were resuspended with 100 μL PBS solution, followed by 13000 rpm centrifugation for two minutes. Various macrocyclic peptides were dissolved in DMSO and then diluted to the required concentration. The untreated group was resuspended with 100 μL PBS, and the peptide treatment group was resuspended with 98 μL ddH_2O, then 1 μL 100 mM EDTA and 1 μL 5 mM different peptides were added, mixed evenly, and reacted for 5 minutes. After the reaction, the sample volume 4 μL cells was spotted on plates, and the first ten-fold dilution was carried out, and the second to five ten-fold dilution was carried out after mixing. Finally, after all the plates were finished, the plates were put into a 37 °C biochemical incubator for inverted culture overnight. Finally, the plate was scanned and counted.

Without EDTA，the peptide treatment group was resuspended with 99 μL ddH_2O, then 1 μL 5 mM different peptides was added, mixed evenly, and reacted for 5 minutes.

Hemolysis assay

Various macrocyclic peptides were dissolved in DMSO and then diluted to the required concentration. The hemolysis assay was carried out with mice blood and diluted ten-fold with 0.9% NaCl to obtain 10% (v/v) red blood cell suspension. First, the red blood cell suspension was centrifuged at 1000 rpm for 15 min, the supernatant was carefully sucked off, and the operation was repeated twice, then resuscitated with 0.9% NaCl, and 200 μL of red blood cell suspension was taken as the untreated group. 2 μL DMSO was added to the negative control group, and 2 μL macrocyclic peptides was added to the experimental treatment group. In the positive control group, the red blood cell suspension was centrifuged at 1000 rpm for 10 min, and the supernatant was absorbed for 200 μL ddH_2O. The samples were incubated at 37 °C for 80 min. After the reaction,

the samples were centrifuged at 1000 rpm for 10 min , and take pictures of the pellet after centrifugation. Then, the supernatant from each sample was absorbed into the 96-well plate to ensure that there could be no air bubbles in each hole. The absorbance of the supernatant at the wavelength of 545 nm was measured by microplate reader, and the percentage of hemolysis of each group was calculated.

$$\text{Percentage of hemolysis} = \frac{OD_{\text{treatment}} - OD_{\text{Untrated}}}{OD_{\text{postive}} - OD_{\text{Untreated}}} \times 100\%$$

Evaluation of R9-conjugated macrocyclic peptides against Gram-negative pathogens

Remove the previously preserved *E. coli* W3110 cells from the -80 °C refrigerator, scrape a little, inoculate it into 1 mL LB liquid medium, and culture overnight in 37 °C shaker. The strain was inoculated into fresh LB liquid medium according to the inoculation ratio of 1:100 and incubated at 37 °C until the OD_{600} reached about 0.47～0.53. First, 100 µL *E. coli* W3110 cells was taken, then centrifuged for 2 minutes at 13000 rpm, then the supernatant was absorbed and discarded, and then the cells were resuspended with 100 µL PBS solution, followed by 13000 rpm centrifugation for two minutes. Various fluorinated macrocyclic peptides conjugated with R9 were dissolved in DMSO and then diluted to the required concentration. The untreated group was resuspended with 100 µL PBS, and the peptide treatment group was resuspended with 99 µL ddH_2O, then 1 µL 0.5 mM different fluorinated macrocyclic peptides conjugated with R9 were added, mixed evenly, and reacted for 15 minutes. After the reaction, the sample volume 4 µL cells was spotted on plates, and the first ten-fold dilution was carried out, and the second to five ten-fold dilution was carried out after mixing. Finally, after all the plates were finished, the plates were put into a 37 °C biochemical incubator for inverted culture overnight. Finally, the plate was scanned and counted. Fluorinated macrocyclic peptides conjugated with R9 against *K. pneumonia* KP-D367 cells, *A. baumannii* Ab6 cells, *S. typhimurium* SL1344 cells, and *P. aeruginosa* PAO1 cells by the same procedures as *E. coli* W3110 , the slight difference is that the centrifugation time of *S. typhimurium* SL1344 cells and *P. aeruginosa* PAO1 cells is 3 minutes.

Propidium Iodide (PI) assay

Thaw the previously preserved *E. coli* W3110 cells from the -80 °C freezer. Scrape a small amount and inoculate it into 1 mL of LB liquid medium. Culture overnight in a 37°C shaker. Next day, inoculate the strain into fresh LB liquid medium at a 1:100 ratio and incubate at 37 °C until the OD_{600} reaches approximately 0.47 to 0.53. Take 100 µL of the *E. coli* W3110 cells and centrifuge for 2 min at 13,000 rpm. Discard the supernatant. For the untreated group, resuspend the cells in 100 µL of PBS. For the peptide treatment group, resuspend the cells in 99 µL of ddH_2O and add 1

μL of 0.5 mM of the different peptides. Mix thoroughly and let react for 15 min. After the treatment, take 80 μL of each sample and incubate with PI at a final concentration of 25 μg/mL for 15 min. After incubation, wash the samples with the same amount of PBS, centrifuge, and resuspend at a 10-fold concentration. Observe the images using a Nikon Eclipse Ci-L Plus microscope.

Protein leakage assay

Thaw the previously preserved *E. coli* W3110 cells from the -80 °C freezer. Scrape a small amount and inoculate it into 1 mL of LB liquid medium. Incubate overnight in a 37°C shaker. Next day, transfer the culture to fresh LB liquid medium at a 1:100 inoculation ratio and incubate at 37 °C until the OD_{600} reaches approximately 0.47 to 0.53. Collect 300 μL of the *E. coli* W3110 cells and centrifuge for 2 min at 13,000 rpm, discarding the supernatant afterward. Resuspend the untreated group in 300 μL of PBS, and the peptide treatment group in 297 μL of ddH_2O. Add 3 μL of 0.5 mM different peptides to the treatment group, mix well, and allow to react for 15 min. After the treatment, resuspend all samples in 15 μL of 6×SDS loading buffer and 15 μL of 10% SDS. All samples were heated at 99.9°C for 15 min, followed by 10% SDS-PAGE analysis and staining with Coomassie brilliant blue R250.

Scanning electron microscopy

Thaw the previously preserved *E. coli* W3110 cells from the -80°C freezer. Scrape a small amount and inoculate it into 1 mL of LB liquid medium. Incubate overnight in a 37°C shaker. The next day, inoculate the strain into fresh LB liquid medium at a 1:100 ratio and incubate at 37°C until the OD_{600} reaches approximately 0.47 to 0.53. Take 500 μL of *E. coli* W3110 cells, centrifuge for 10 min at 4000 rpm, and discard the supernatant. Resuspend the cells in 500 μL of PBS, then centrifuge again at 4000 rpm for 10 min and discard the supernatant. Dissolve various macrocyclic peptides in DMSO and dilute to the required concentration. Resuspend the untreated group in 500 μL of PBS. For the peptide treatment group, resuspend in 495 μL of ddH_2O, add 5 μL of 0.2 mM or 0.5 mM peptides, mix well, and let react for 4 h.

After the treament, spot 4 μL of the sample on plates. Perform the first ten-fold dilution and mix, then continue with the second to fifth ten-fold dilutions. Incubate the plates inverted overnight in a 37°C incubator. Scan and count the colonies. For the remaining 492 μL, centrifuge at 6100g for 10 min, discard the supernatant, add 500 μL of ddH_2O, and let it stand for 15 min. Centrifuge again at 6100g for 10 min, discard the supernatant, and add 500 μL of ddH_2O containing 2.5% glutaraldehyde. Resuspend and fix the cells overnight at 4°C. Centrifuge at 6100g for 10 min, remove the supernatant, and wash twice with ddH_2O, letting it stand for 15 min each time, followed by centrifugation at 6100g for 10 min. Discard the supernatant. Dehydrate the sample with ethanol at concentration gradients of 30%, 60%, and 90%, without resuspension, centrifuging at 6100g for

10 min and discarding the supernatant after each step. Finally, use 100% ethanol, rest for 15 min, centrifuge at 6100g for 10 min, discard the supernatant, and repeat with 100% ethanol, leaving about 10 μL. Then the critical point was dried and sprayed with gold, and the images were observed by Quanta 250 FEG.

Prediction of binding sites between peptides and the BAM complex

Firstly, the fpocket algorithm was used, a rapid method for pocket prediction based on the three-dimensional structure of BAM complex (PDB ID: 5AYW). This method establishes a grid surrounding the protein, positioning alpha spheres on grid points devoid of spatial conflicts with protein atoms, subsequently utilizing a clustering algorithm to ascertain whether these regions constitute potential binding pockets. Alpha spheres were designated with radii ranging from 3.4Å to 6.2Å, with a prerequisite of at least 15 alpha spheres for a region to be considered a pocket. The method employs pairwise single linkage clustering with a Euclidean distance metric.

Secondly, PointSite was utilized for the prediction of potential binding sites within the BAM structure. Similar to fpocket, PointSite relies exclusively on the structural integrity of protein, independent of ligand data. PointSite was executed using the default parameter (voting number 25) to ascertain each amino acid's propensity to constitute a pocket. Utilizing these assessments, pockets identified by P2Rank—a separate program designed for the prediction of ligand-binding pockets—were re-ranked, selecting the top three based on their average scores.

Lastly, the application of the PepNN algorithm, a deep attention model formulated for the identification of peptide binding sites, facilitated the incorporation of macrocyclic peptide data in evaluating potential binding sites on BAM complex. Macrocyclic peptides were segmented at non-standard sites into multiple fragments, each containing solely standard amino acids (Table S1). PepNN evaluated each amino acid for its potentiality as a peptide binding site. An average of the scores for all fragments associated with the same cyclic peptide was computed to derive the final predictive outcomes.

Supplementary Data

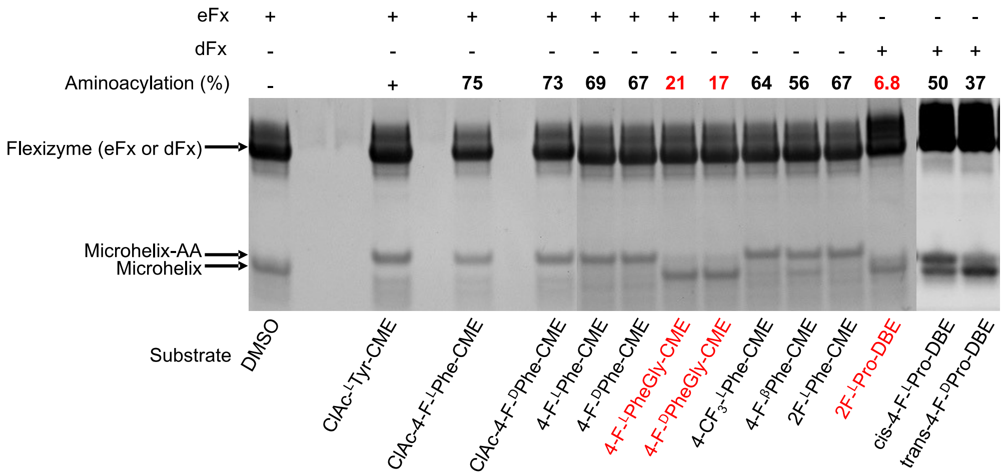

Figure S1 | Acid-PAGE analysis of aminoacylation of microhelix RNA with CME-activated fluorinated amino acids and DBE-activated fluorinated amino acids under the same reaction conditions that 50 mM HEPES-KOH (pH 7.5), 2 h. Arrows indicate the positions of flexizyme (eFx or dFx), aminoacyl-microhelix RNA (Microhelix-AA), and unreacted microhelix RNA (Microhelix). ClAc-^{L}Tyr-CME was used as a positive control, DMSO was used as a negative control. The result shows that the efficiency of charging of 4-F-^{L}PheGly-CME, 4-F-^{D}PheGly-CME and 2F-^{L}Pro-DBE onto RNA was low.

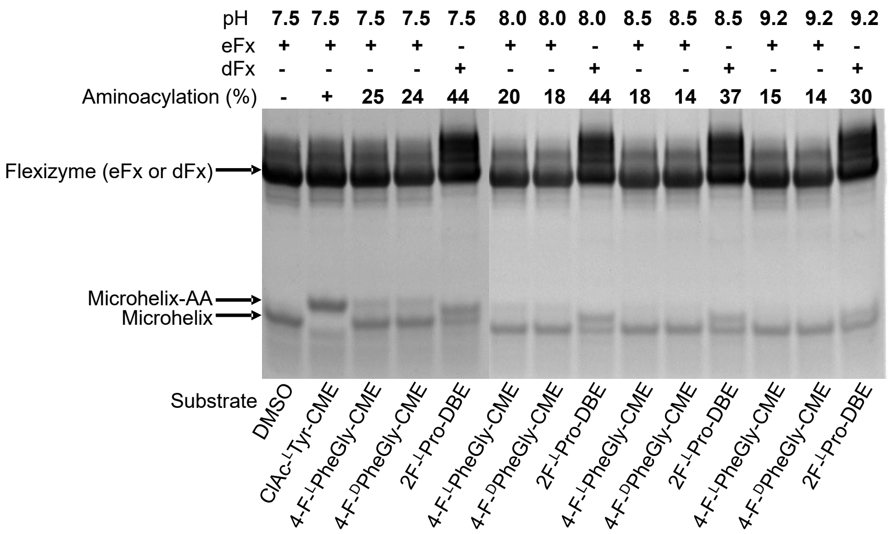

Figure S2 | Acid-PAGE analysis of aminoacylation of microhelix RNA with CME-activated fluorinated amino acids and DBE-activated fluorinated amino acids under various reaction pH. Arrows indicate the positions of flexizyme (eFx or dFx), aminoacyl-microhelix RNA (Microhelix-AA), and unreacted microhelix RNA (Microhelix). ClAc-^{L}Tyr-CME was used as a positive control, DMSO was used as a negative control. The experiments demonstrate that the optimal reaction conditions of 4-F-^{L}PheGly-CME, 4-F-^{D}PheGly-CME and 2F-^{L}Pro-DBE are 50 mM HEPES-KOH (pH 7.5), 12 h.

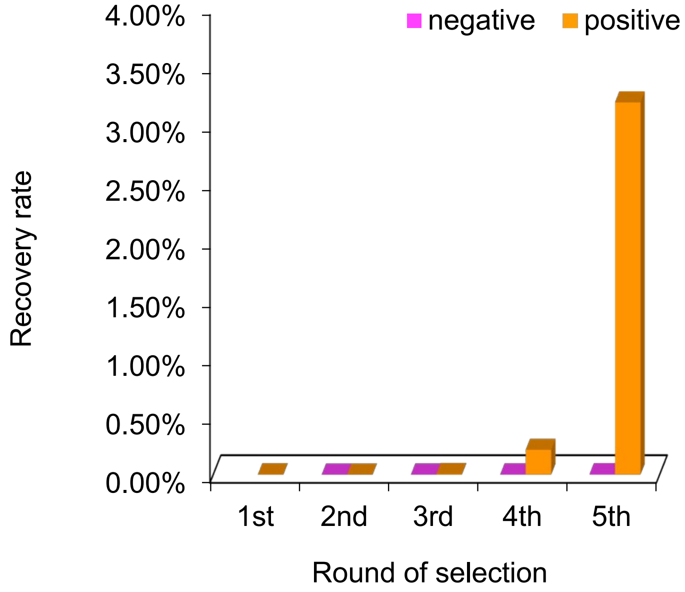

Figure S3 | RaPID selections against EphA2 using fluorinated macrocyclic peptides library. The ClAc-4-F-^{D}Phe was assigned to the initiator AUG codon, and 2F-^{L}Phe was assigned to the elongator AUG codon. The recovery of cDNAs were quantitatively determined by real-time PCR and were shown on the figure (orange bars: cDNAs eluted from EphA2 immobilized beads; magenta bars: cDNAs eluted from beads). The recovery ratio was defined as the ratio of recovered cDNAs amount from EphA2 immobilized beads (orange) or magnetic beads-only (magenta) to the amount of the input mRNA/cDNA-peptide fusion. Negative selection was not performed at the 1st round. The sequence of cDNA library was analyzed at the end of the 5th round of selection

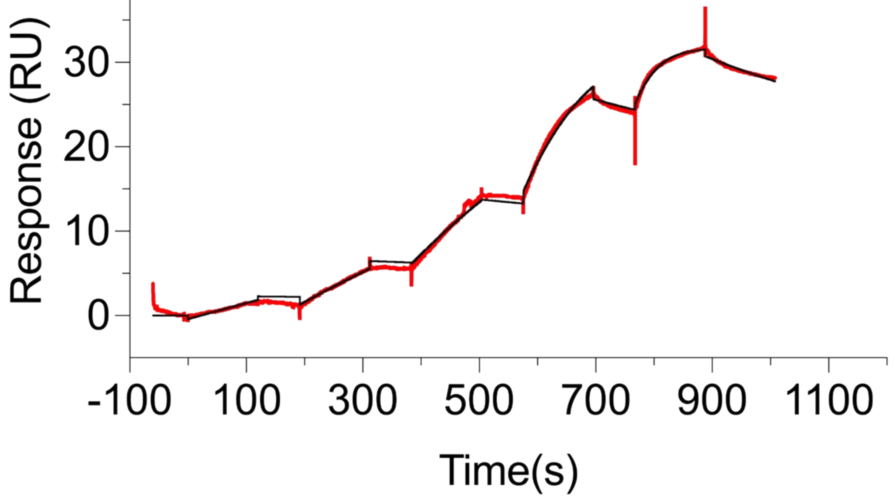

Figure S4 | SPR results of the Ep-F2-M2. Five different concentrations (Ep-F2-M2: 0.5, 1.0, 2.5, 5, 10 nM) were injected for SPR measurements. The K_D value of Ep-F2-M2 was determined to be 0.29 nM.

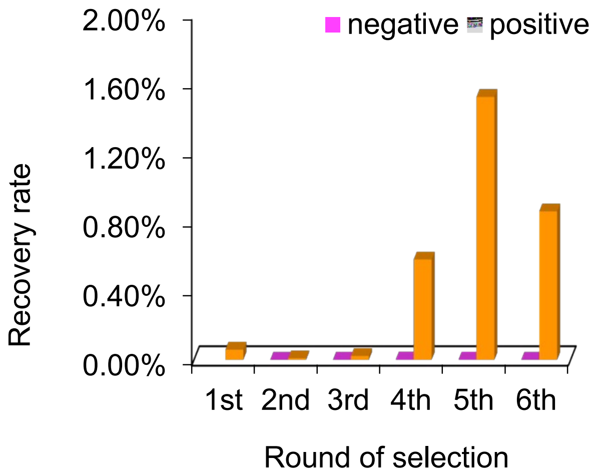

Figure S5 | RaPID selections against the BAM complex using fluorinated macrocyclic peptides library. The ClAc-4-F-^{D}Phe was assigned to the initiator AUG codon, and 4-F-^{D}Phe was assigned to the elongator AUG codon. The recovery of cDNAs were quantitatively determined by real-time PCR and were shown on the figure (orange bars: cDNAs eluted from BAM complex immobilized beads; magenta bars: cDNAs eluted from beads). The recovery ratio was defined as the ratio of recovered cDNAs amount from BAM complex immobilized beads (orange) or magnetic beads-only (magenta) to the amount of the input mRNA/cDNA-peptide fusion. Negative selection was not performed at the 1st round. The sequence of cDNA library was analysed at the end of the 6th round of selection.

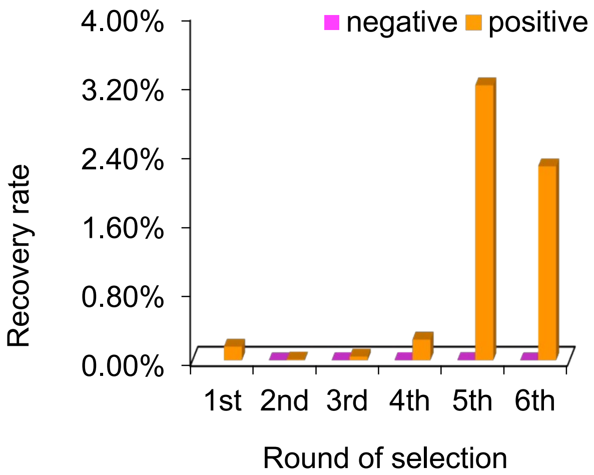

Figure S6 | RaPID selections against the BAM complex using fluorinated macrocyclic peptides library. The ClAc-4-F-^{D}Phe was assigned to the initiator AUG codon, and 4-F-*β*-Phe was assigned to the elongator AUG codon. The recovery of cDNAs were quantitatively determined by real-time PCR and were shown on the figure (orange bars: cDNAs eluted from BAM complex immobilized beads; magenta bars: cDNAs eluted from beads). The recovery ratio was defined as the ratio of recovered cDNAs amount from BAM complex immobilized beads (orange) or magnetic beads-only (magenta) to the amount of the input mRNA/cDNA-peptide fusion. Negative selection was not performed at the 1st round. The sequence of cDNA library was analyzed at the end of the 6th round of selection.

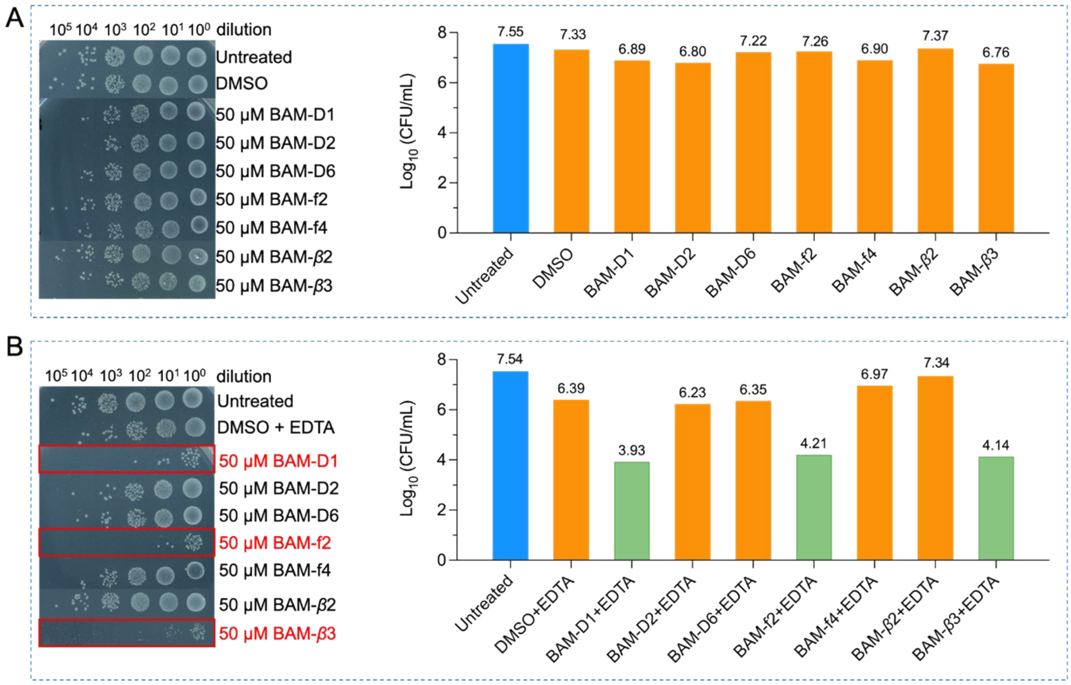

Figure S7 | Results of macrocyclic peptides and fluorinated macrocyclic peptide against *E. coli* W3110. (A) Survival of *E. coli* W3110 cells in logarithmic-phase following a 5 min treatment with 50 µM peptides. (B) Survival of *E. coli* W3110 cells in logarithmic-phase following a 5 min treatment with 50 µM peptides plus 1 mM EDTA.

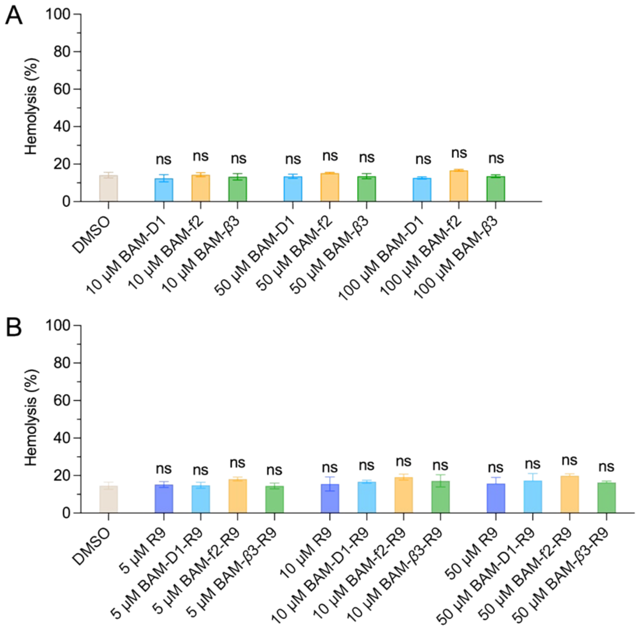

Figure S8 | Hemolysis activity of macrocyclic peptides and CPP-conjugated peptides toward RBCs (mean ± SD, n=3). (A) Hemolysis activity of BAM-D1, BAM-f2 and BAM-β3 toward RBCs. (B) Hemolysis activity of R9, BAM-D1-R9, BAM-f2-R9 and BAM-*β*3-R9 toward RBCs. No significance (ns) indicates a P-value between macrocyclic peptide treatment and DMSO treatment.

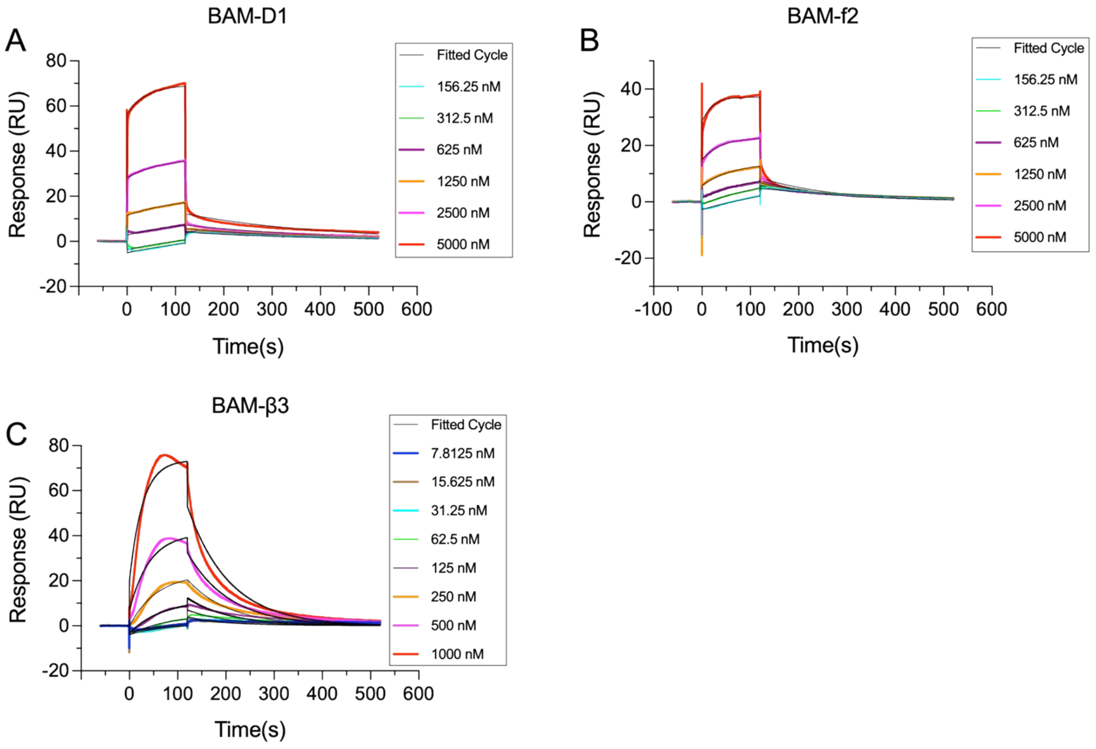

Figure S9 | SPR sensorgrams of macrocyclic peptides targeting BAM complex. SPR sensorgram of (A) BAM-D1, (B) BAM-f2 and (C) BAM-β3. The peptides sequences and the kinetics constants are shown in Table S1.

Table S1 | Binding properties of macrocyclic peptides to BAM complex.

Peptide	Sequence	k_a ($\times 10^4$ $M^{-1}s^{-1}$)	k_d ($\times 10^{-3}$ s^{-1})	K_D (nM)
BAM-D1	Ac-^{D}YLIWYSRSHPFRVCG-NH_2	0.43	3.30	763.0
BAM-f2	Ac-(4-F-^{D}Phe)IWHKSLT(4-F-^{D}Phe)NYLHTCG-NH_2	0.90	6.80	754.9
BAM-β3	Ac-(4-F-^{D}Phe)KHFIY(4-F-β-Phe)YRSPICG-NH_2	2.54	11.52	453.4

The values of k_a, k_d, and K_D were determined by SPR.

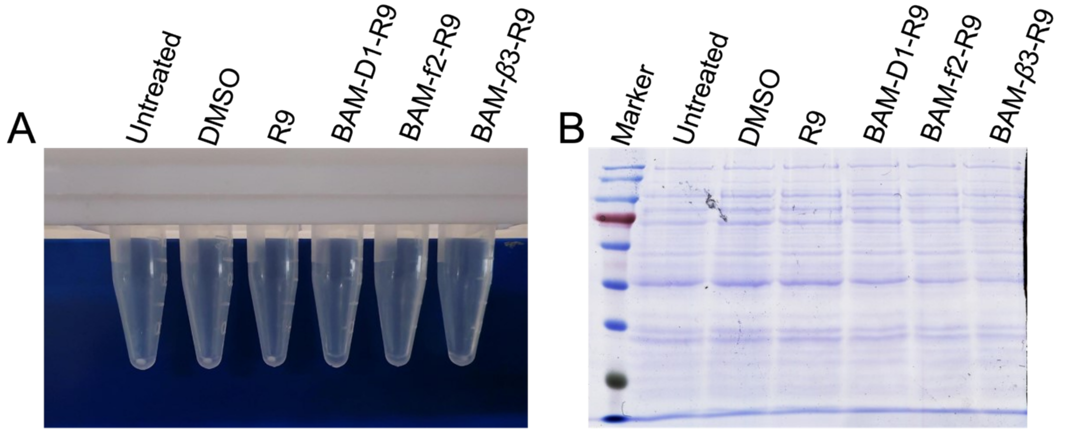

Figure S10 | The result of protein leakage assay. (A) Bacterial precipitation after treatment and centrifugation. (B) The SDS-PAGE of bacterial precipitation.

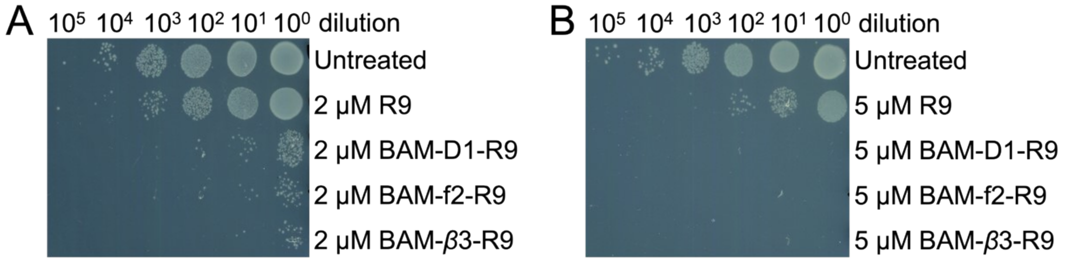

Figure S11 | The result of dot board corresponding to SEM. (A) Treatment group with final concentration of 2 μM. (B) Treatment group with final concentration of 5 μM.

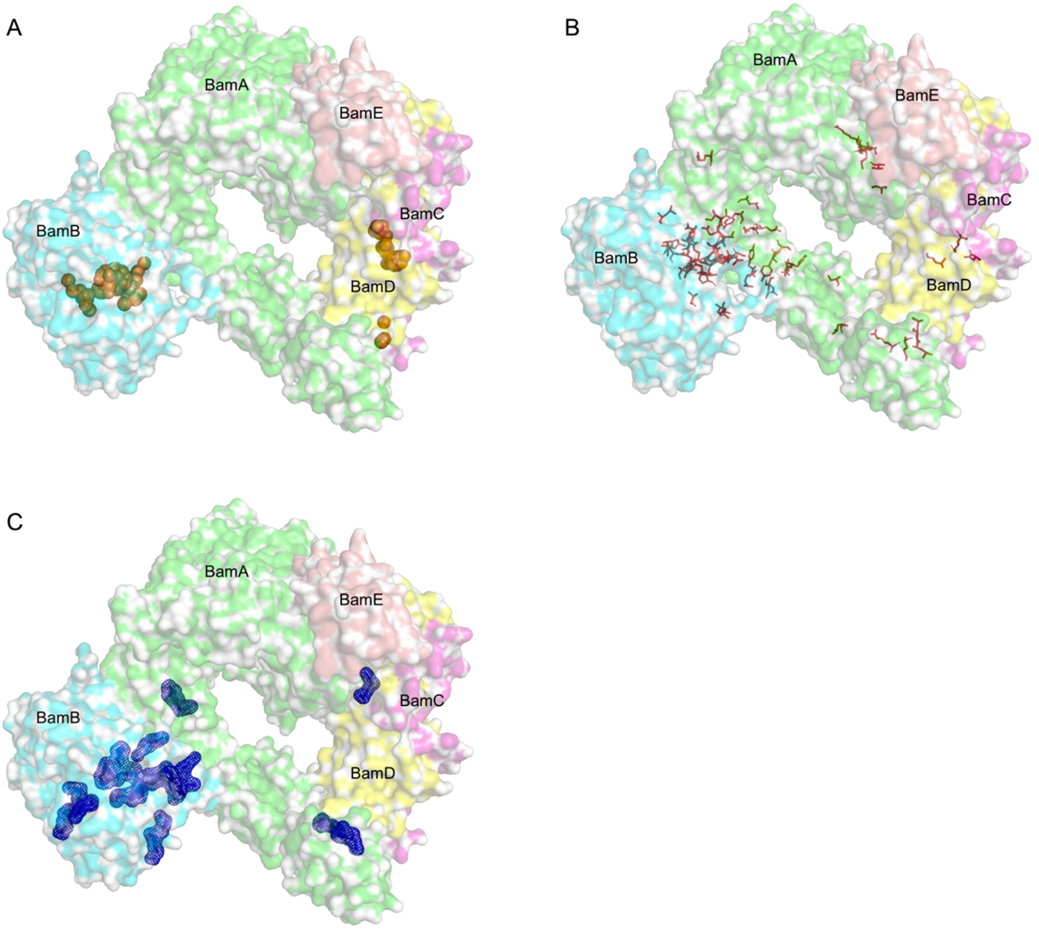

Figure S12 | The predicted binding pockets of fluorinated macrocyclic peptides with BAM complex (PDB ID: 5AYW). BamA is illustrated as a green surface model, while BamB is depicted as a cyan surface model. (A) Predictions made by the PointSite algorithm are represented by orange spheres. (B) Predictions by the PepNN algorithm are shown as red sticks. (C) Predictions from the Fpocket algorithm are visualized as a blue mesh.

Table S2 | The sequence of peptide fragments used to predict the interaction sites of BAM-D1, BAM-f2 and BAM-*β*3 with BAM complex.

peptide fragments	sequence
BAM-D1-r1-7	LIWYSRS
BAM-D1-r2-8	IWYSRSH
BAM-D1-r3-9	WYSRSHP
BAM-D1-r4-10	YSRSHPF
BAM-D1-r5-11	SRSHPFR
BAM-D1-r6-12	RSHPFRV
BAM-D1-r7-13	SHPFRVC
BAM-f2-r1-7	IWHKSLT
BAM-f2-r9-15	NYLHTCG
BAM-*β*3-r1-5	KHFIY
BAM-*β*3-r7-13	YRSPICG

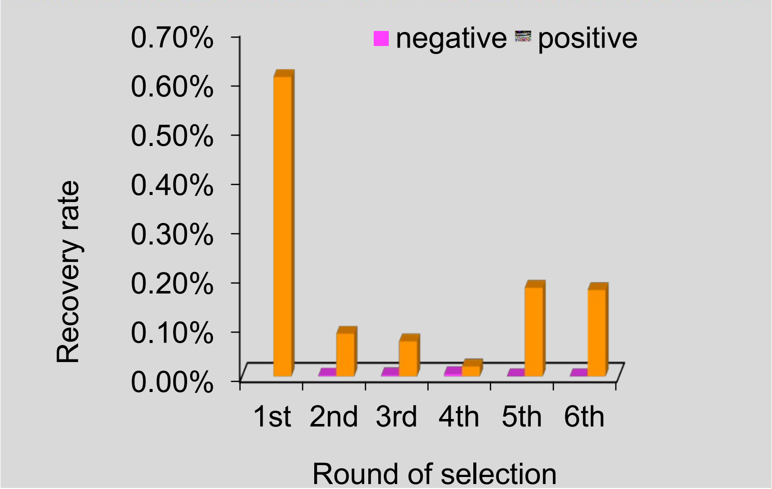

Figure S13 | RaPID selections against the BAM complex using macrocyclic peptides library. The ClAc-^{D}Tyr was assigned to the initiator AUG codon. The recovery of cDNAs were quantitatively determined by real-time PCR and were shown on the figure (orange bars: cDNAs eluted from BAM complex immobilized beads; magenta bars: cDNAs eluted from beads). The recovery ratio was defined as the ratio of recovered cDNAs amount from BAM complex immobilized beads (orange) or magnetic beads-only (magenta) to the amount of the input mRNA/cDNA-peptide fusion. Negative selection was not performed at the 1st round. The sequence of cDNA library was analyzed at the end of the 6th round of selection.

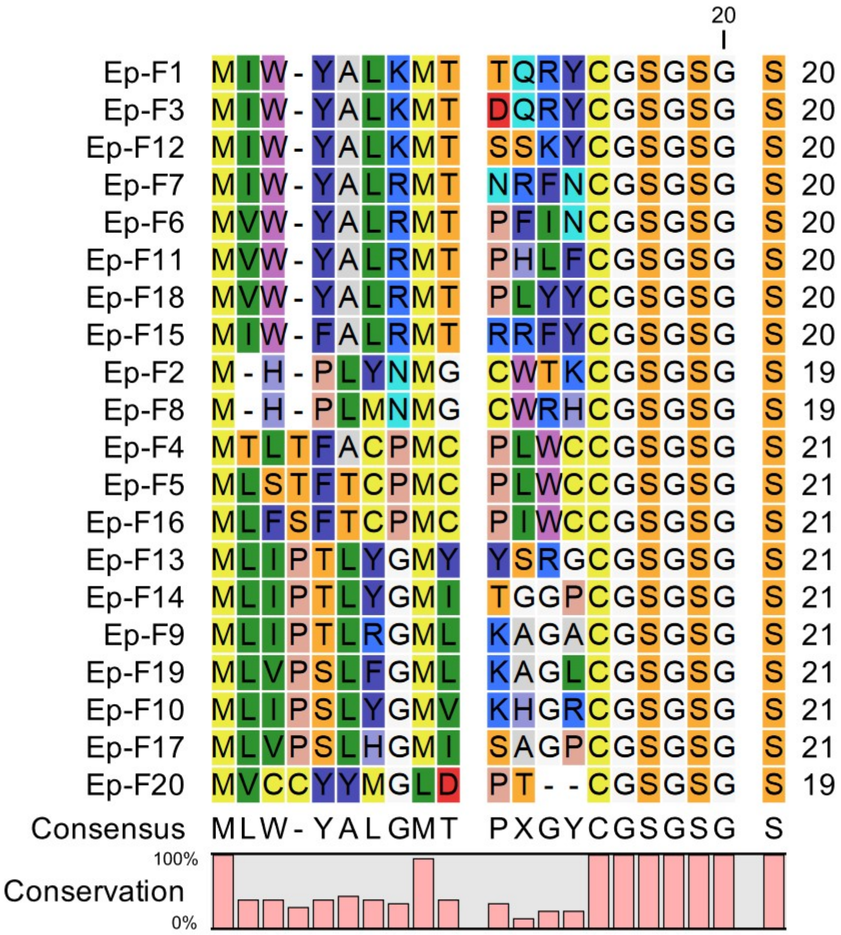

Figure S14 | Selected top 20 fluorinated macrocyclic peptide sequences in consensus with the designed library by targeting EphA2 using the RaPID system. M at the initiation position indicates ClAc-4-F-^{D}Phe while M at the elongation position indicates 2F-^{L}Phe.

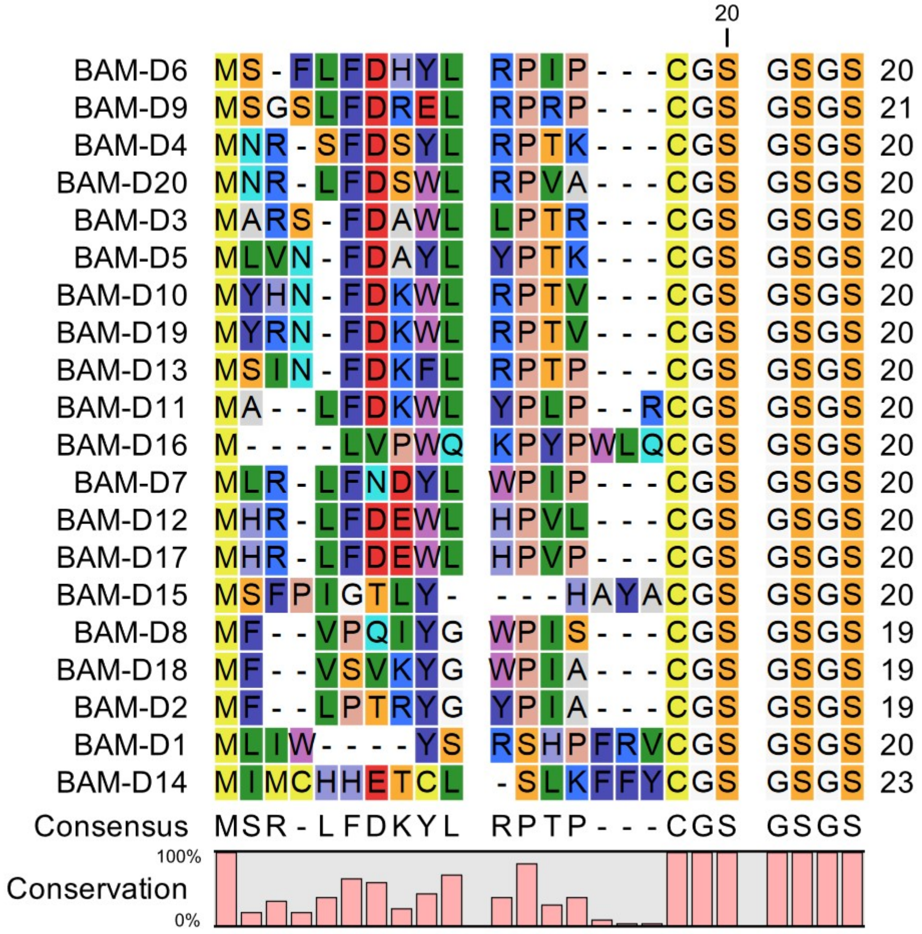

Figure S15 | Selected top 20 macrocyclic peptide sequences in consensus with the designed library by targeting the BAM complex using the RaPID system. M at the initiation position indicates ClAc-^{D}Tyr.

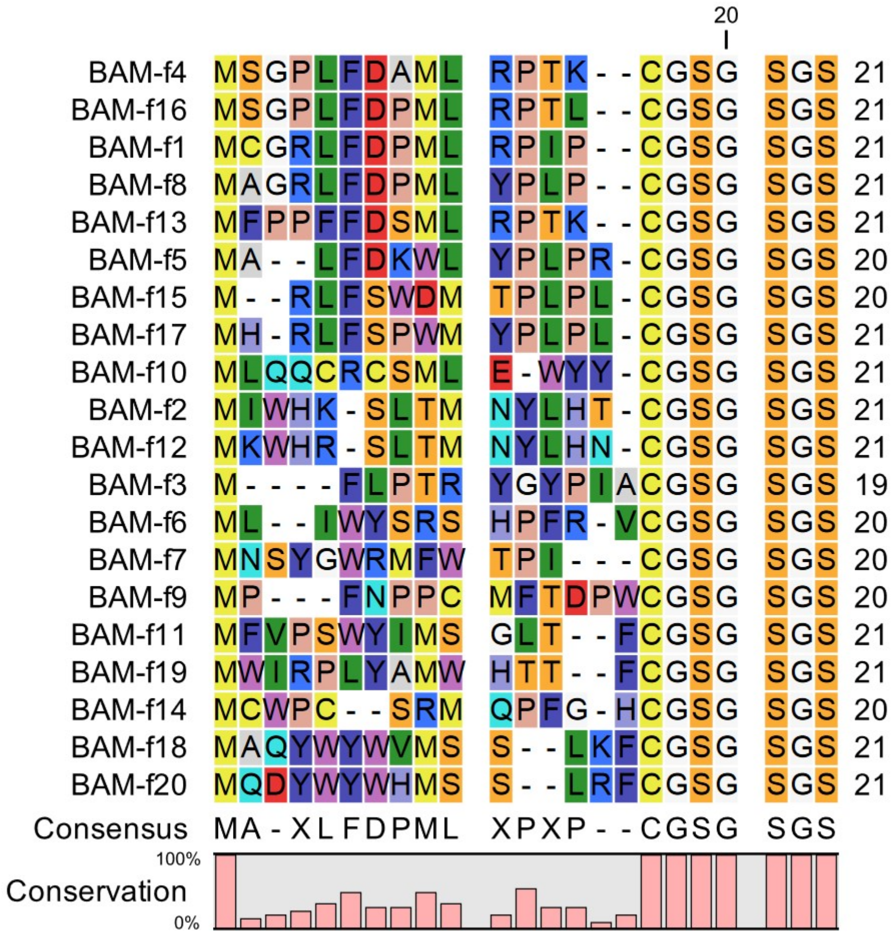

Figure S16 | Selected top 20 fluorinated macrocyclic peptide sequences in consensus with the designed library by targeting the BAM complex using the RaPID system. M at the initiation position indicates ClAc-4-F-^{D}Phe while M at the elongation position indicates 4-F-^{D}Phe.

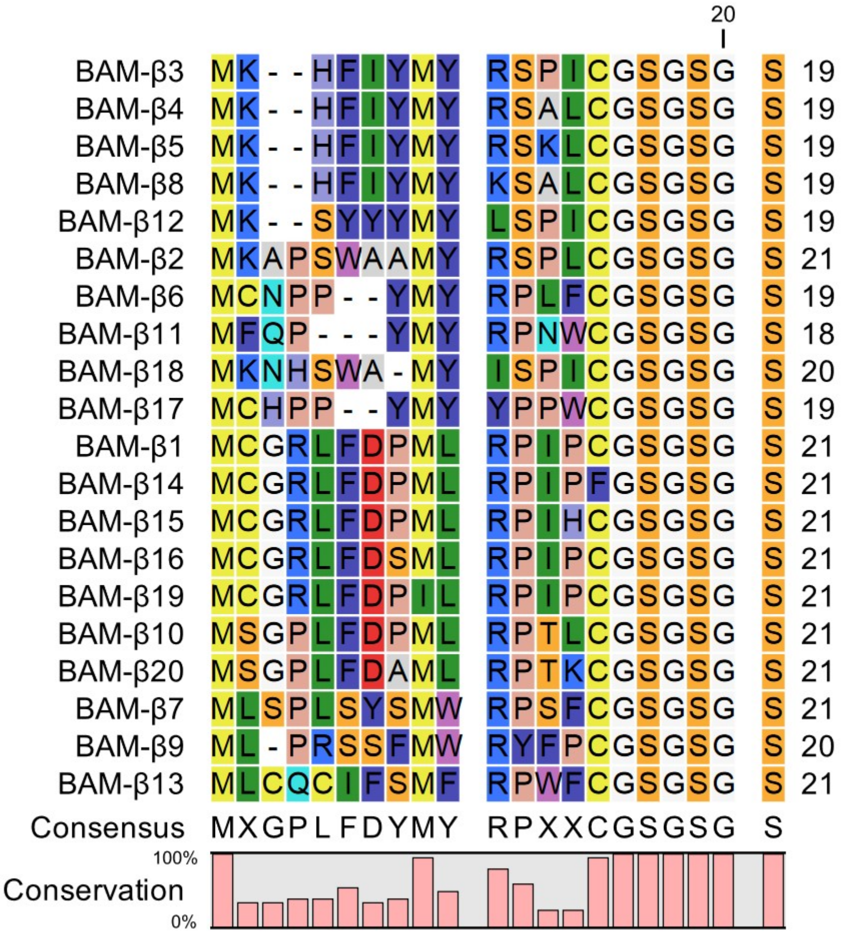

Figure S17 | Selected top 20 fluorinated macrocyclic peptide sequences in consensus with the designed library by targeting the BAM complex using the RaPID system. M at the initiation position indicates ClAc-4-F-^{D}Phe while M at the elongation position indicates 4-F-*β*-Phe.

NMR spectra of activated fluorinated amino acids

^{1}H NMR of ClAc-4-F-[L]Phe-CME

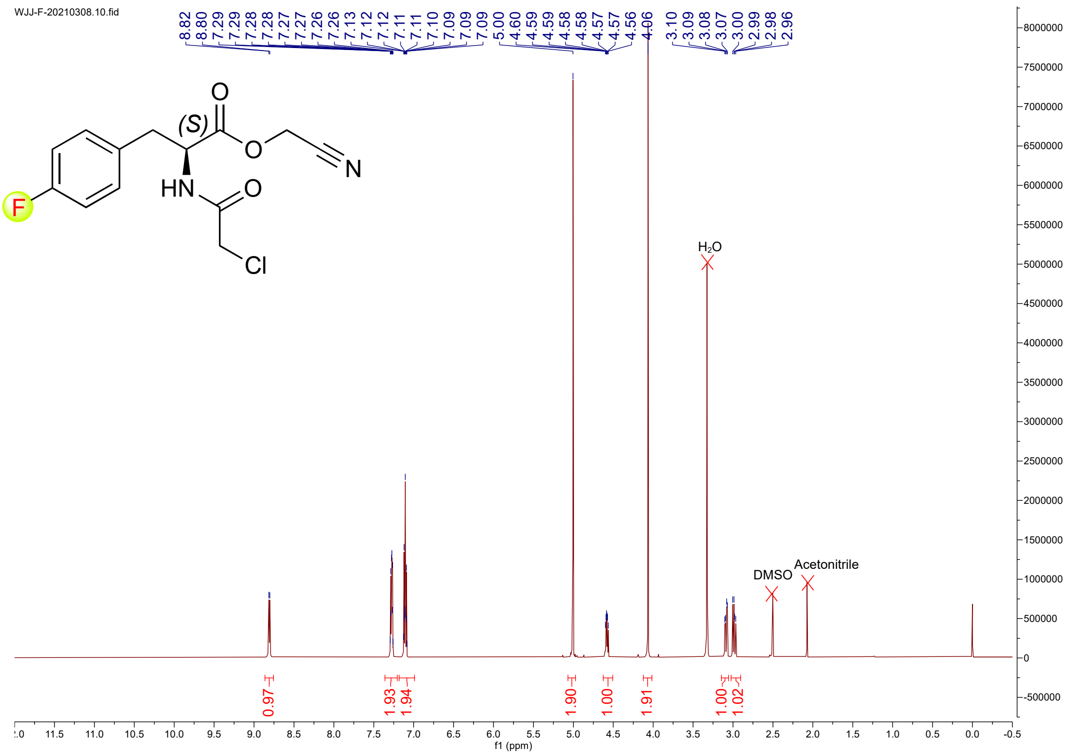

^{13}C NMR of ClAc-4-F-[L]Phe-CME

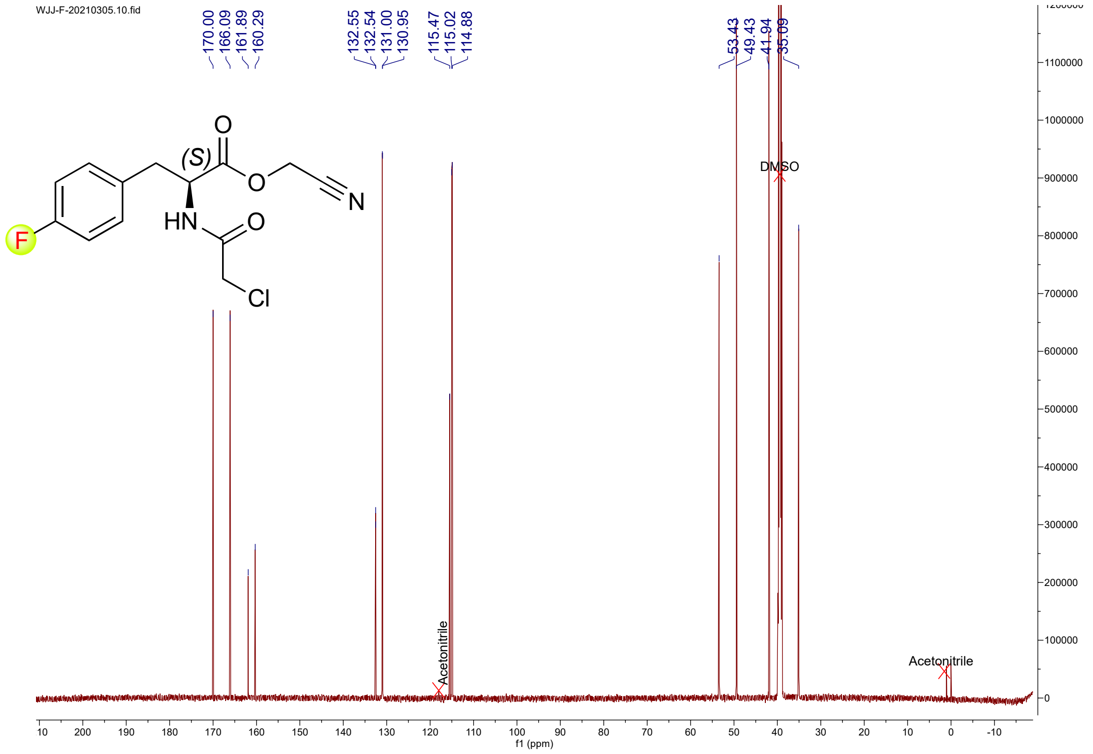

^{19}F NMR of ClAc-4-F-^{L}Phe-CME

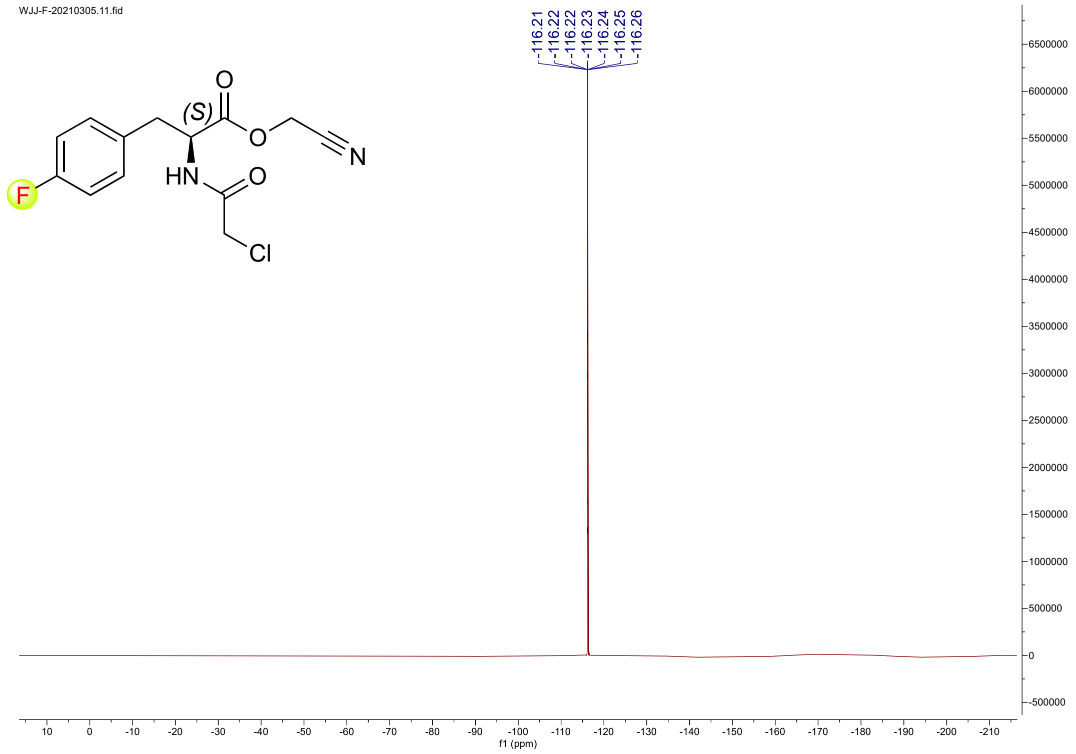

^{1}H NMR of ClAc-4-F-^{D}Phe-CME

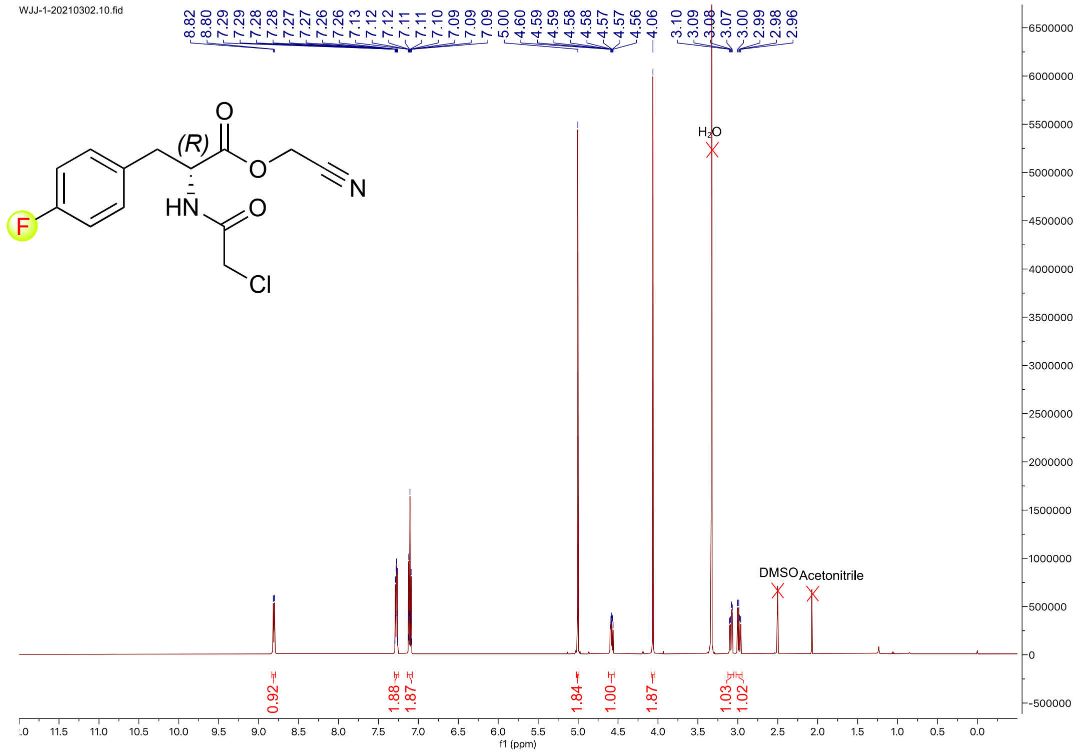

^{13}C NMR of ClAc-4-F-^{D}Phe-CME

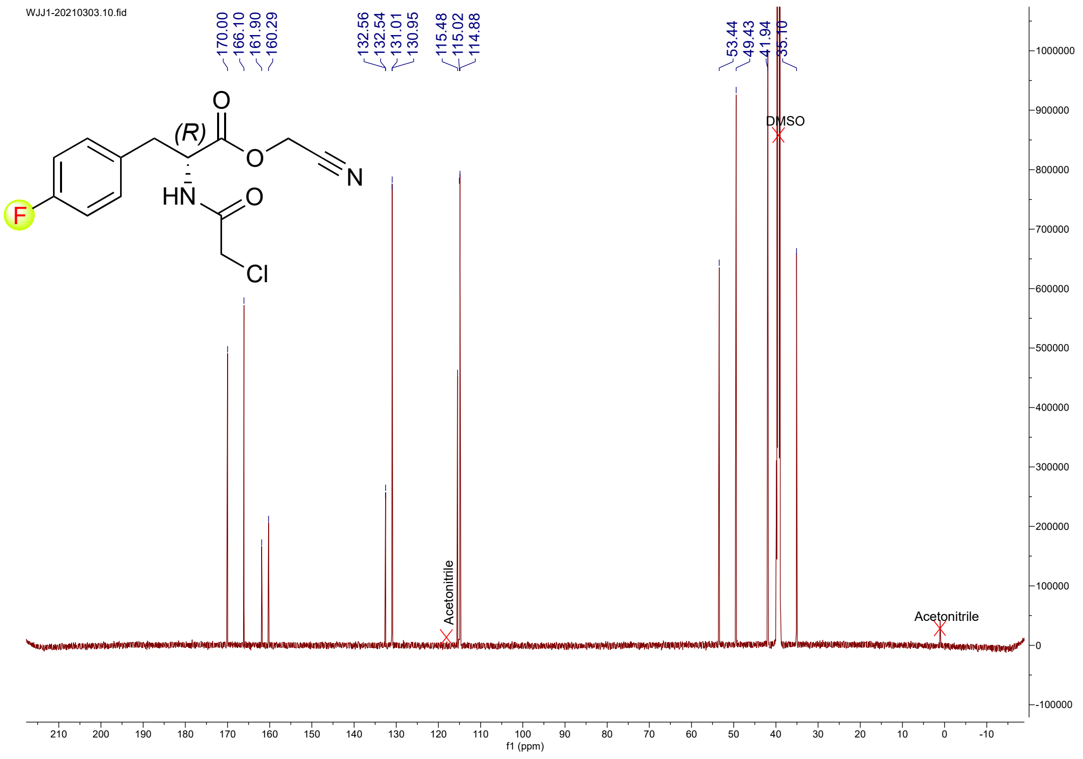

^{19}F NMR of ClAc-4-F-^{D}Phe-CME

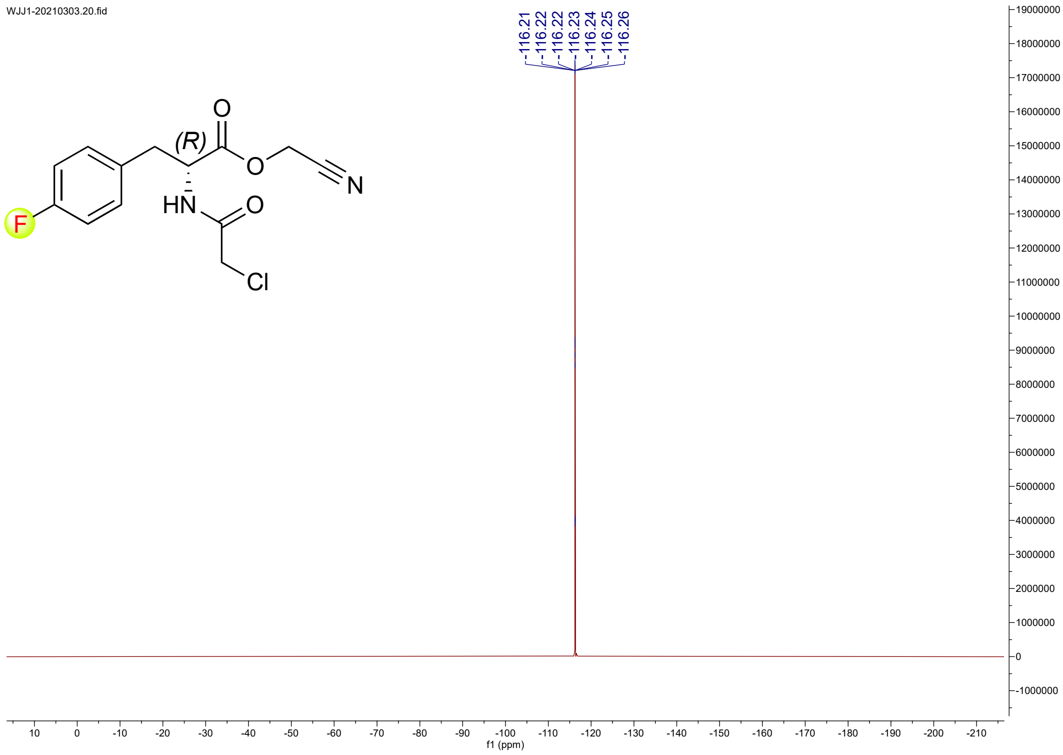

^{1}H NMR of 4-F-^{L}Phe-CME

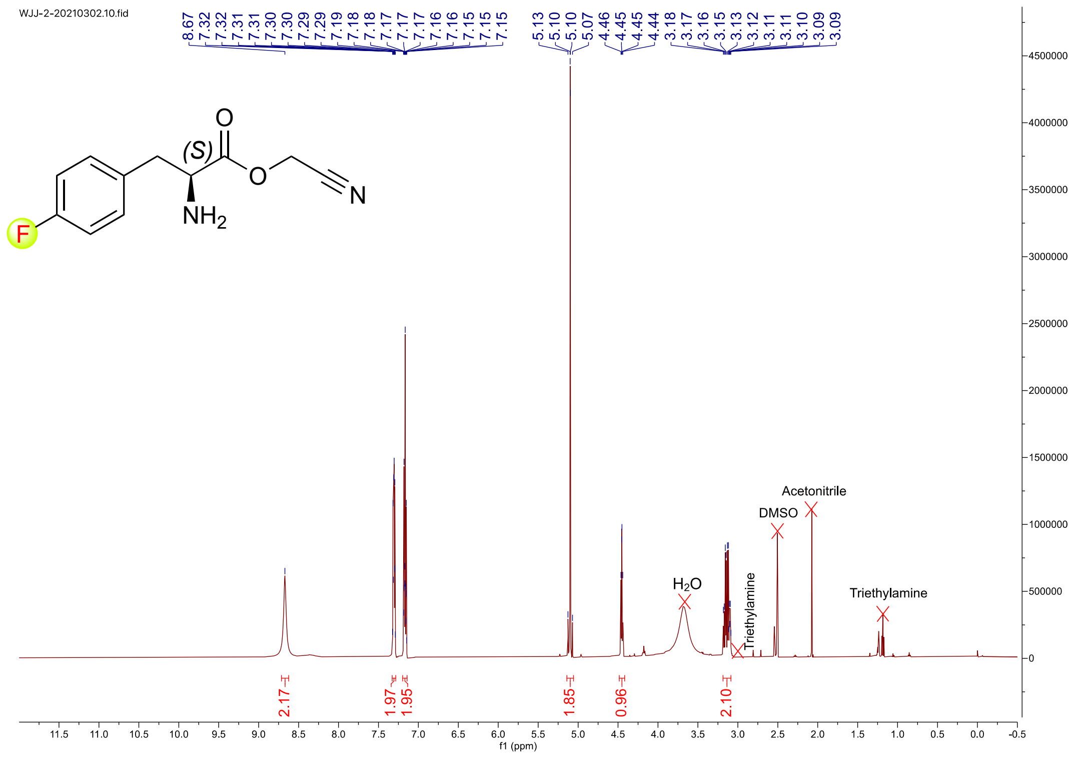

^{13}C NMR of 4-F-^{L}Phe-CME

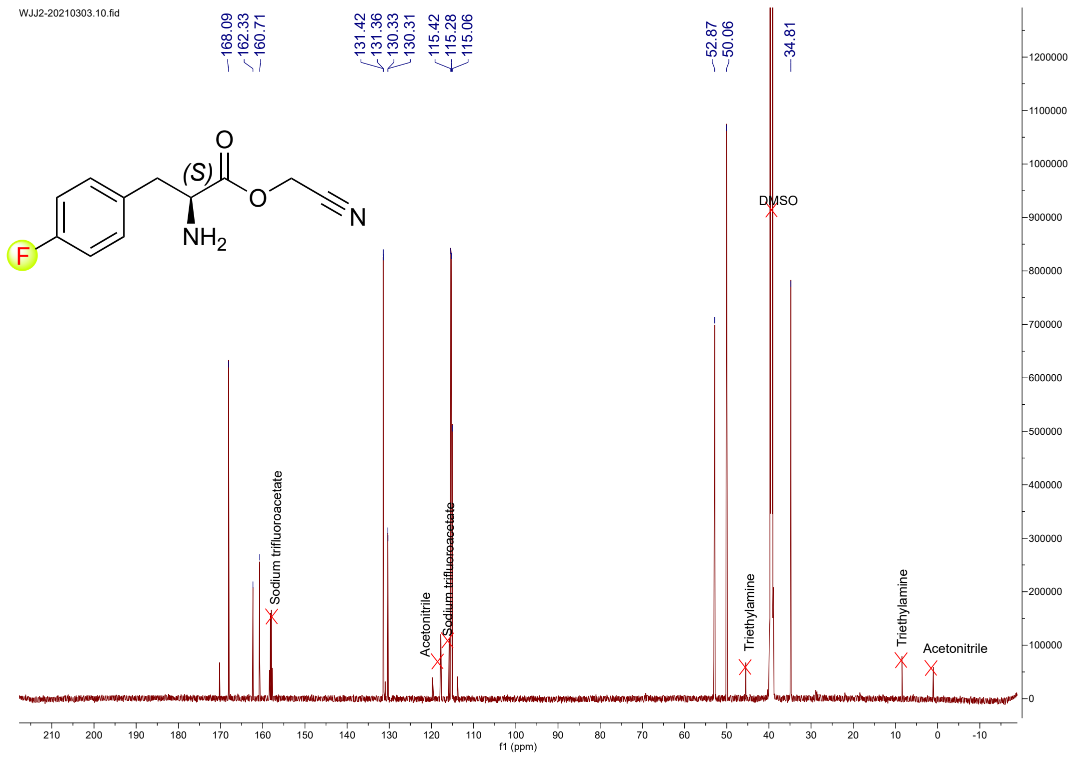

^{19}F NMR of 4-F-^{L}Phe-CME

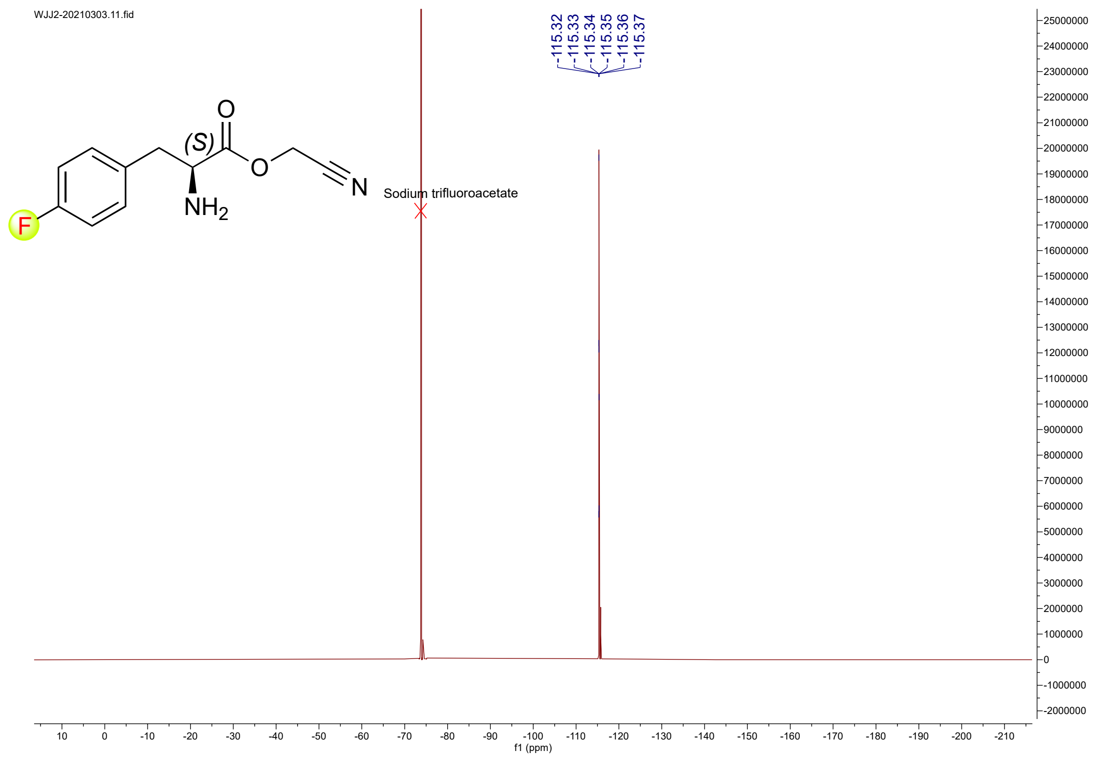

^{1}H NMR of 4-F-^{D}Phe-CME

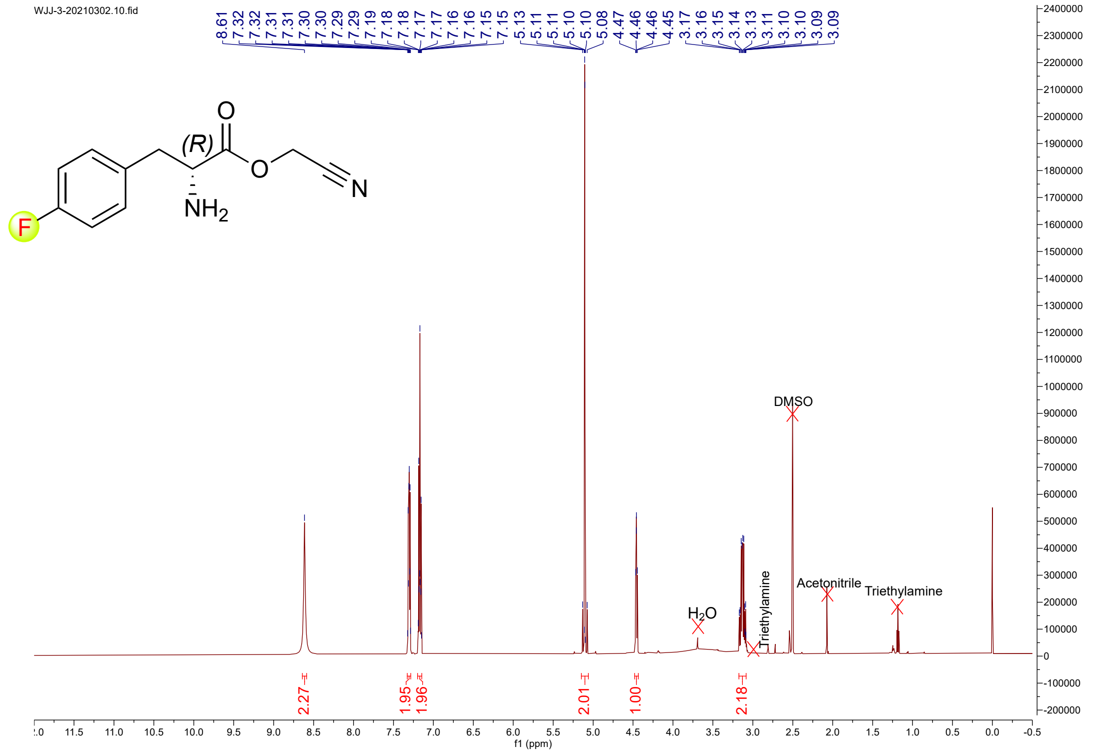

^{13}C NMR of 4-F-^{D}Phe-CME

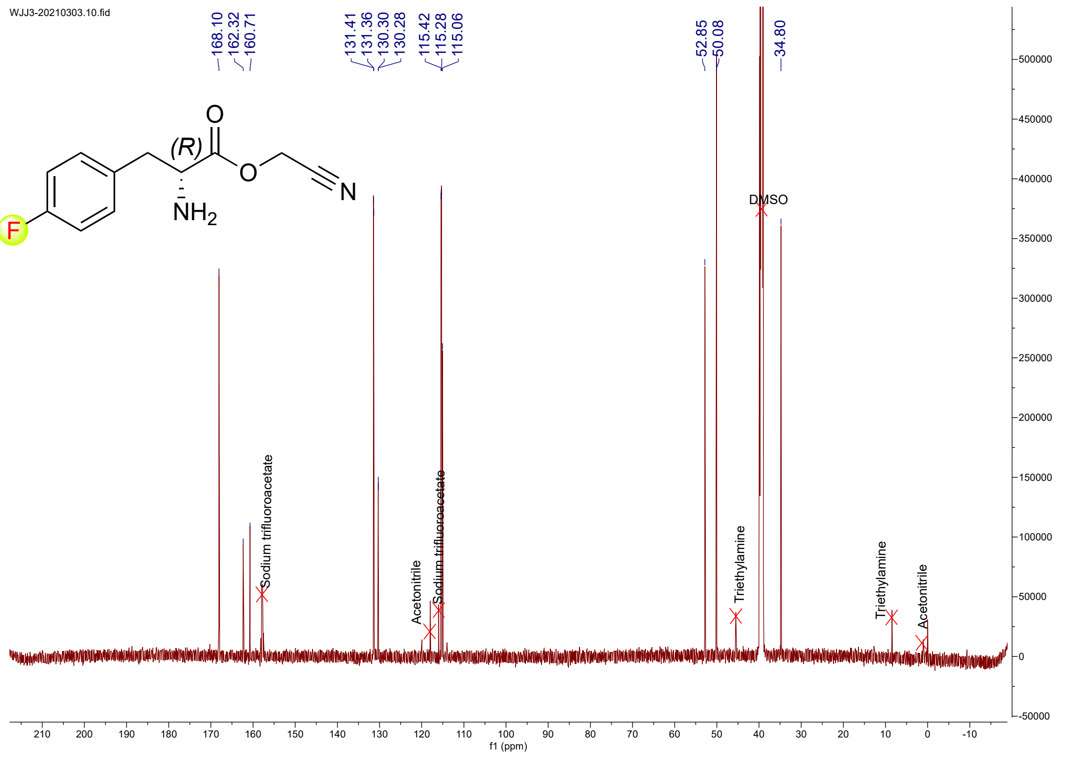

^{19}F NMR of 4-F-^{D}Phe-CME

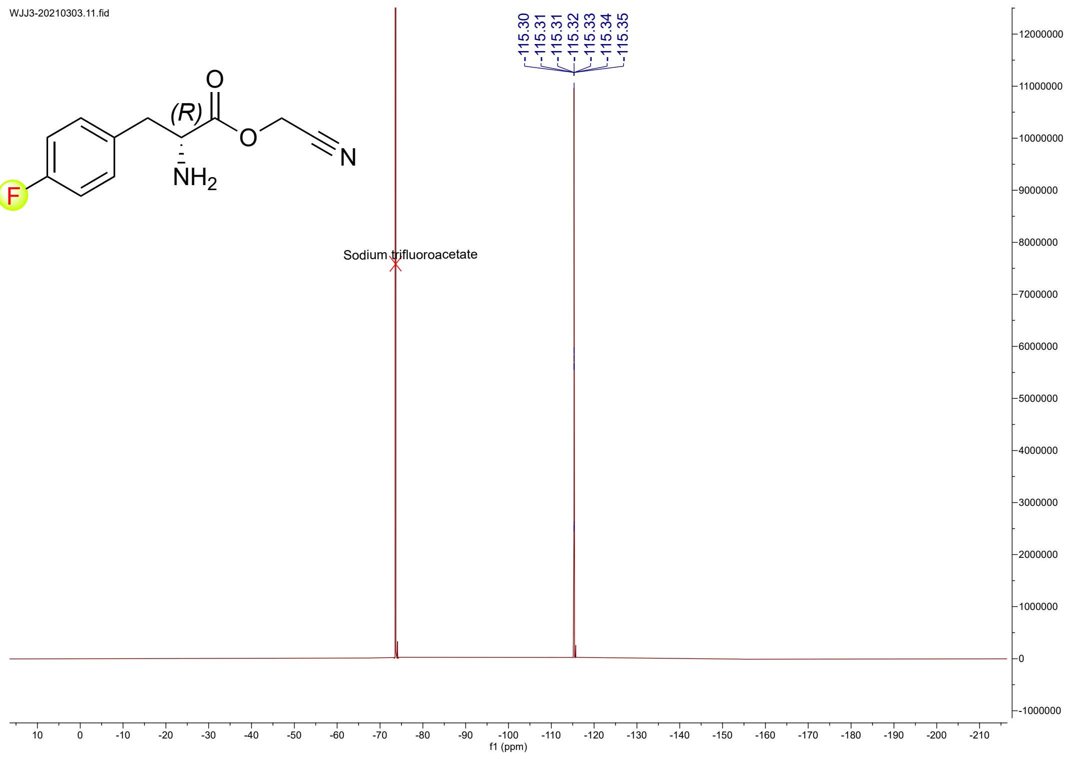

^{1}H NMR of 4-F-^{L}PheGly-CME

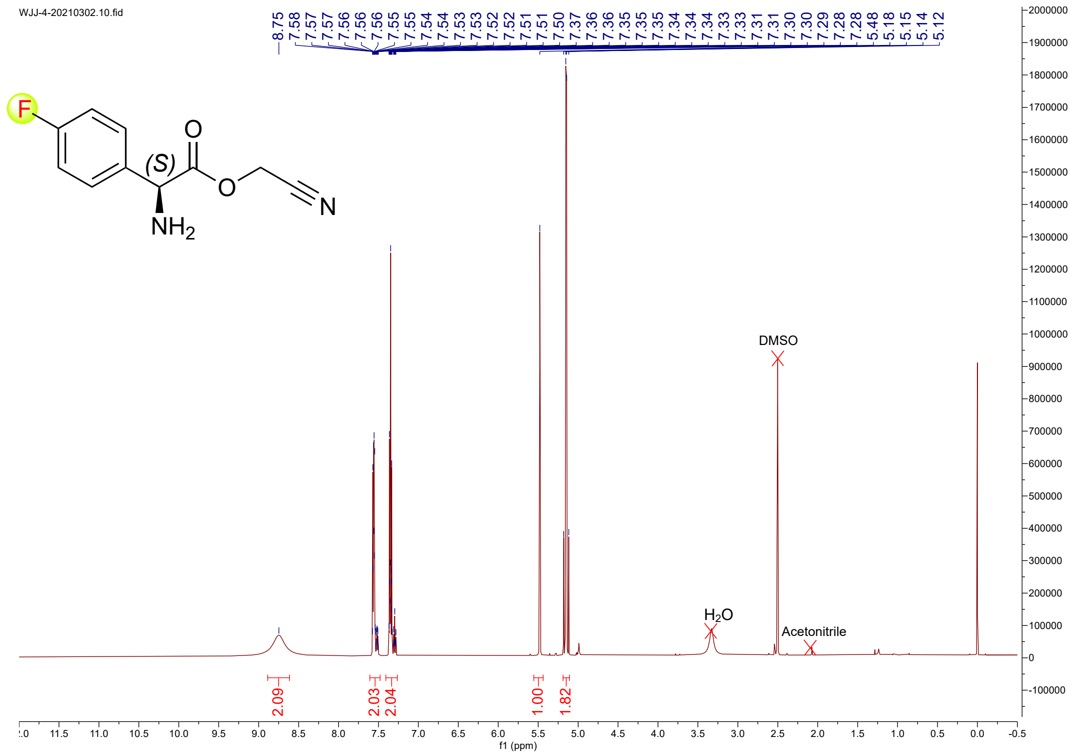

^{13}C NMR of 4-F-^{L}PheGly-CME

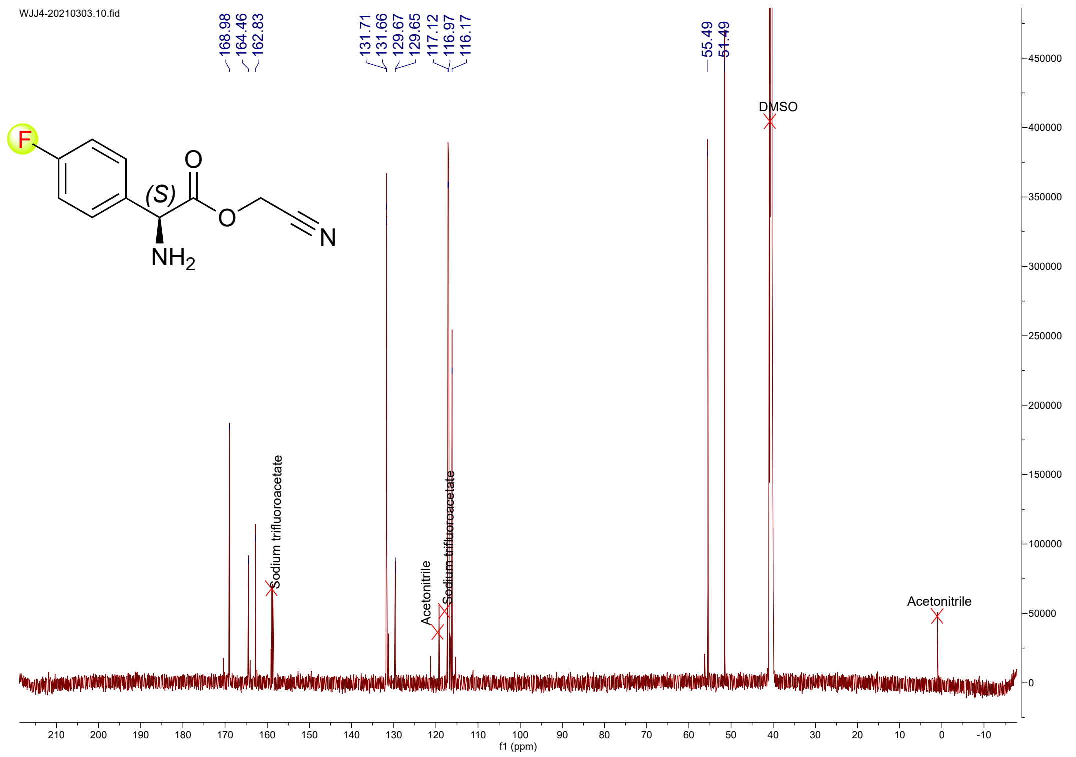

^{19}F NMR of 4-F-^{L}PheGly-CME

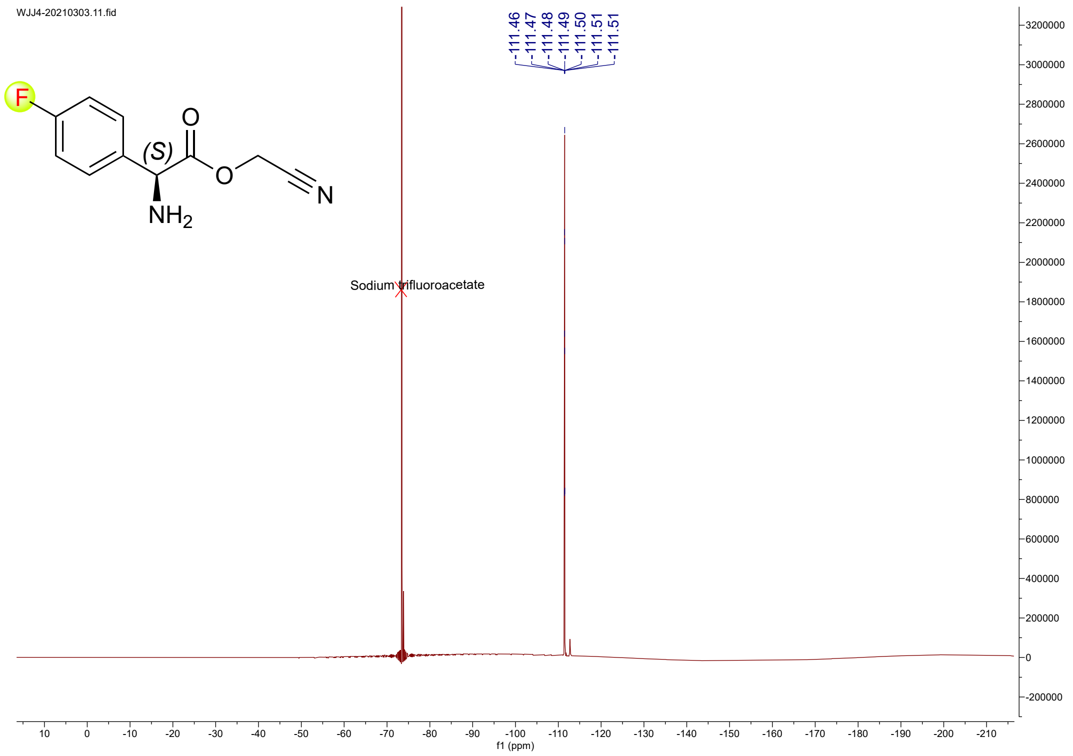

^{1}H NMR of 4-F-^{D}PheGly-CME

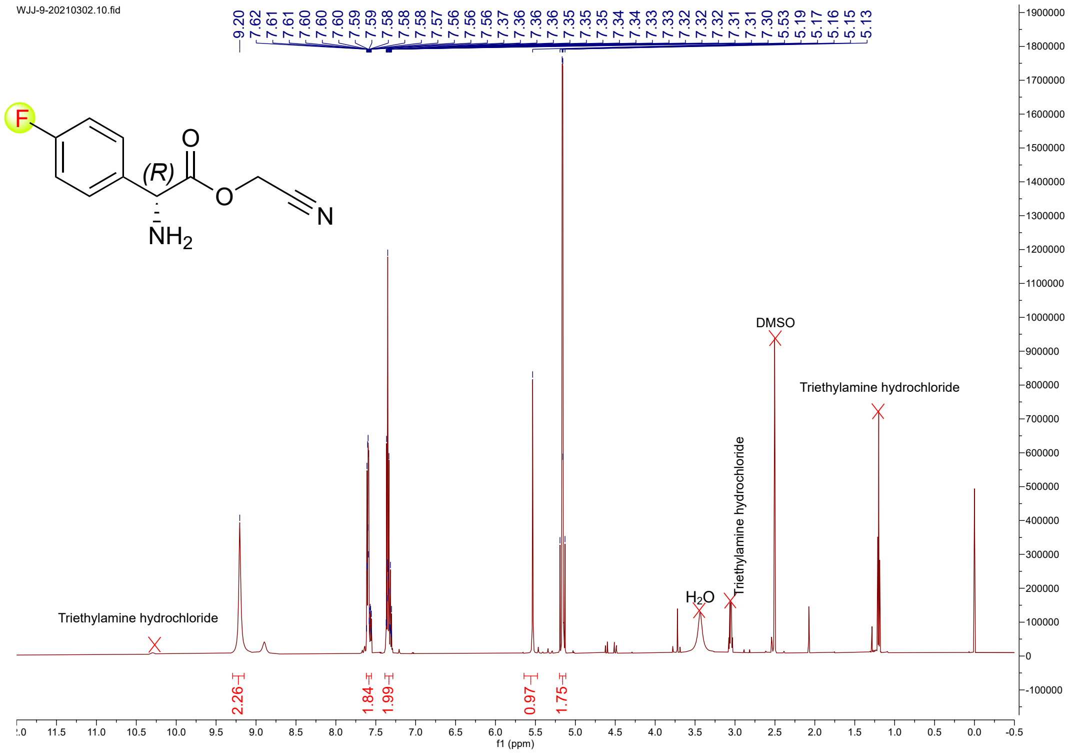

^{13}C NMR of 4-F-^{D}PheGly-CME

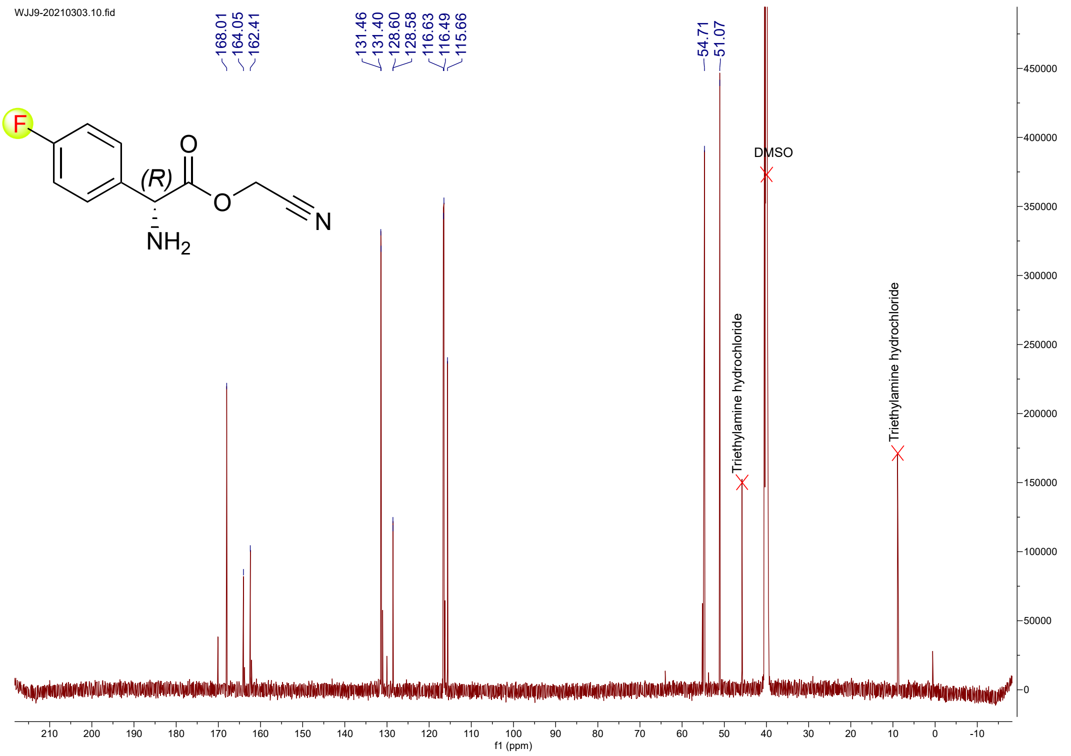

^{19}F NMR of 4-F-^{D}PheGly-CME

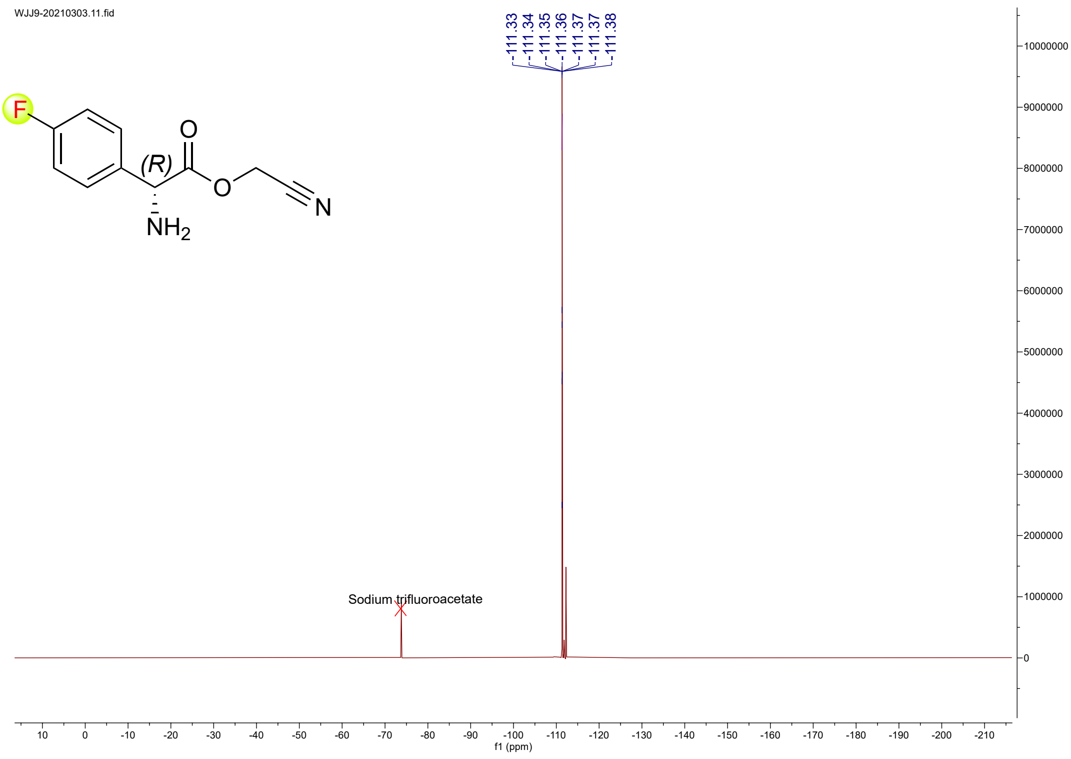

^{1}H NMR of 2F-^{L}Phe-CME

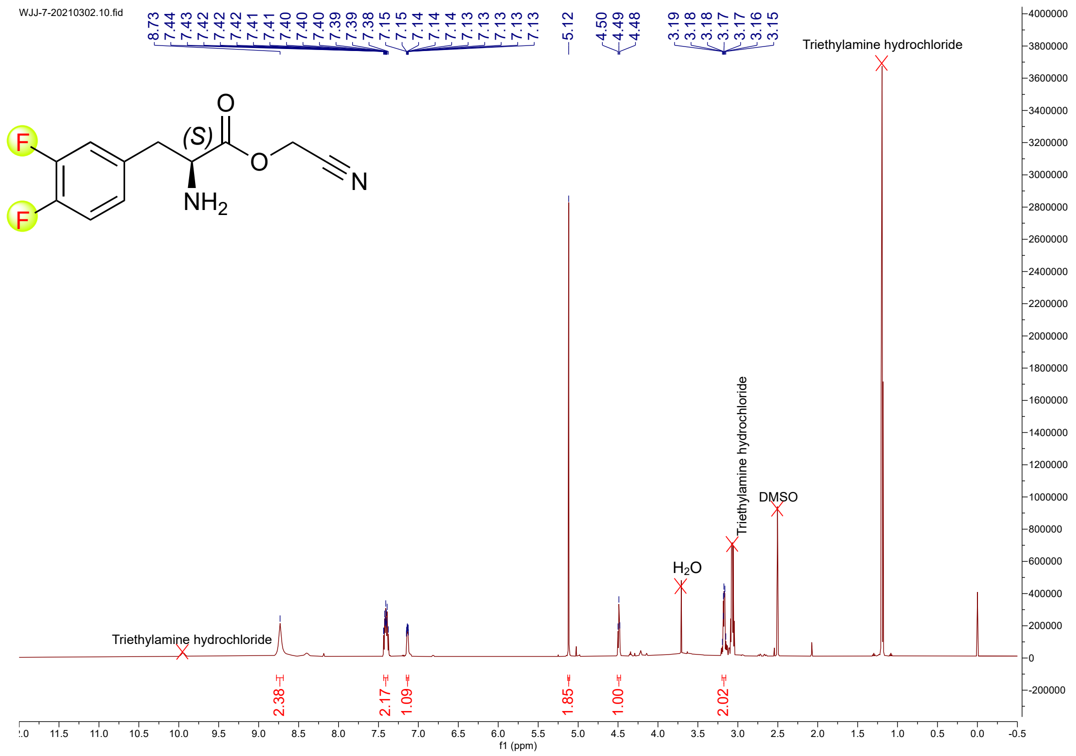

^{13}C NMR of 2F-^{L}Phe-CME

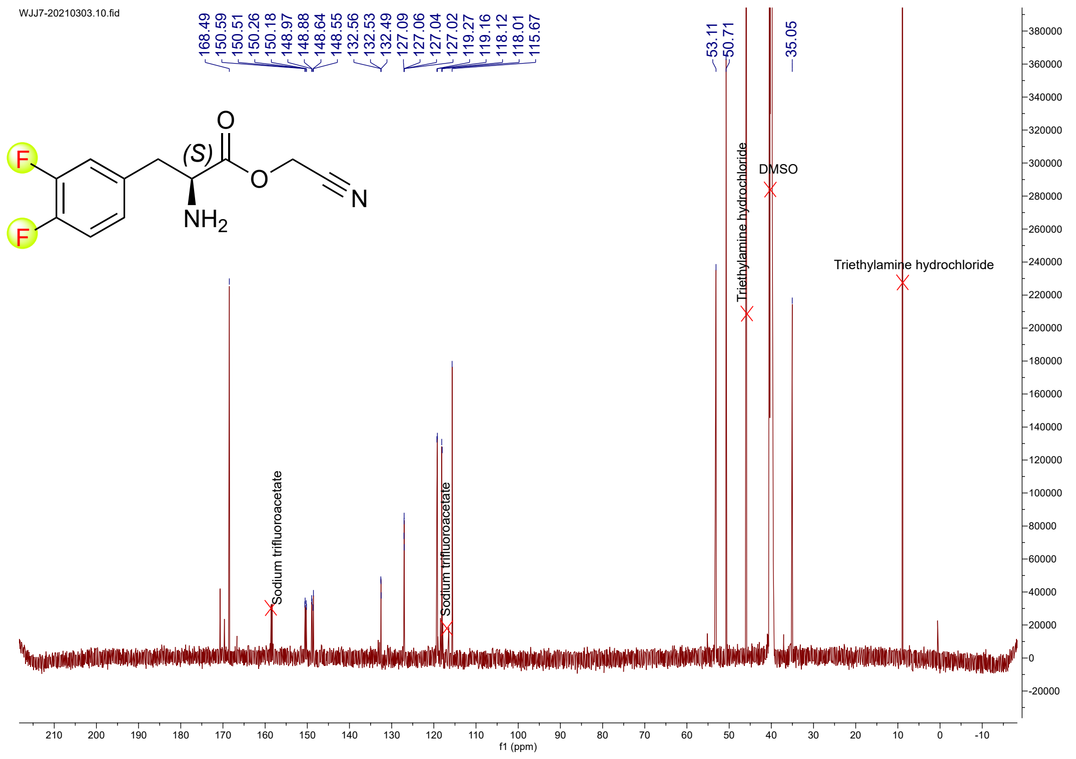

^{19}F NMR of 2F-^{L}Phe-CME

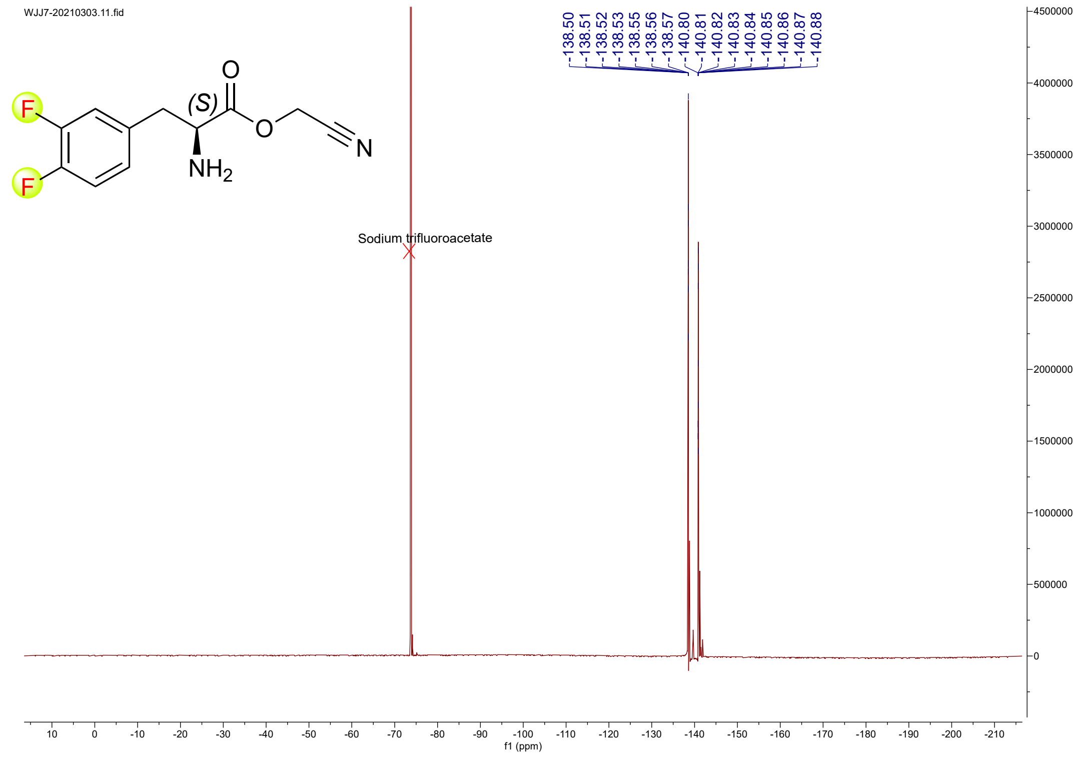

^{1}H NMR of 4-CF_3-^{L}Phe-CME

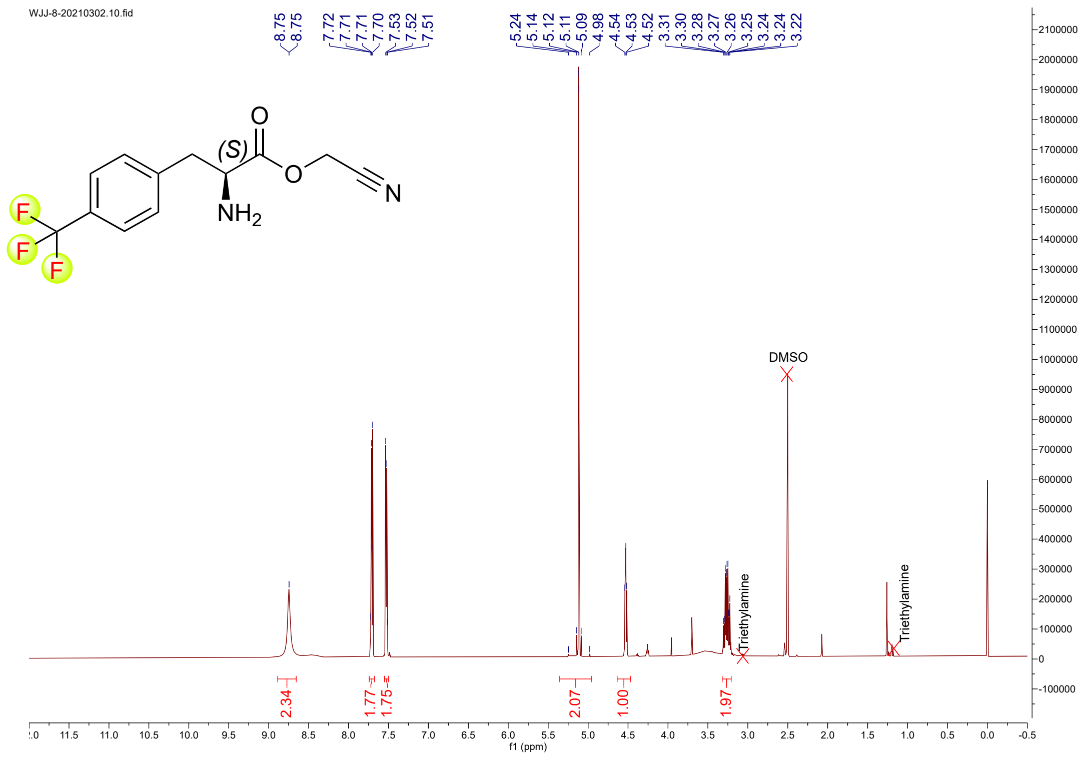

^{13}C NMR of 4-CF_3-^{L}Phe-CME

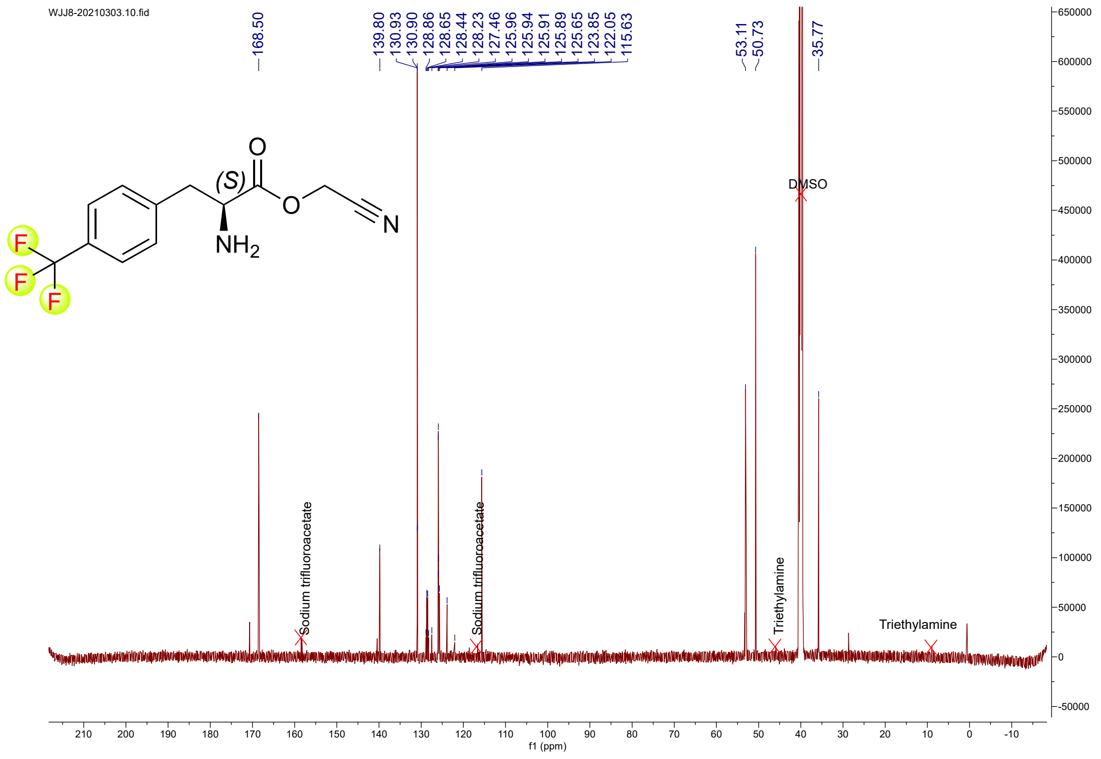

^{19}F NMR of 4-CF_3-^{L}Phe-CME

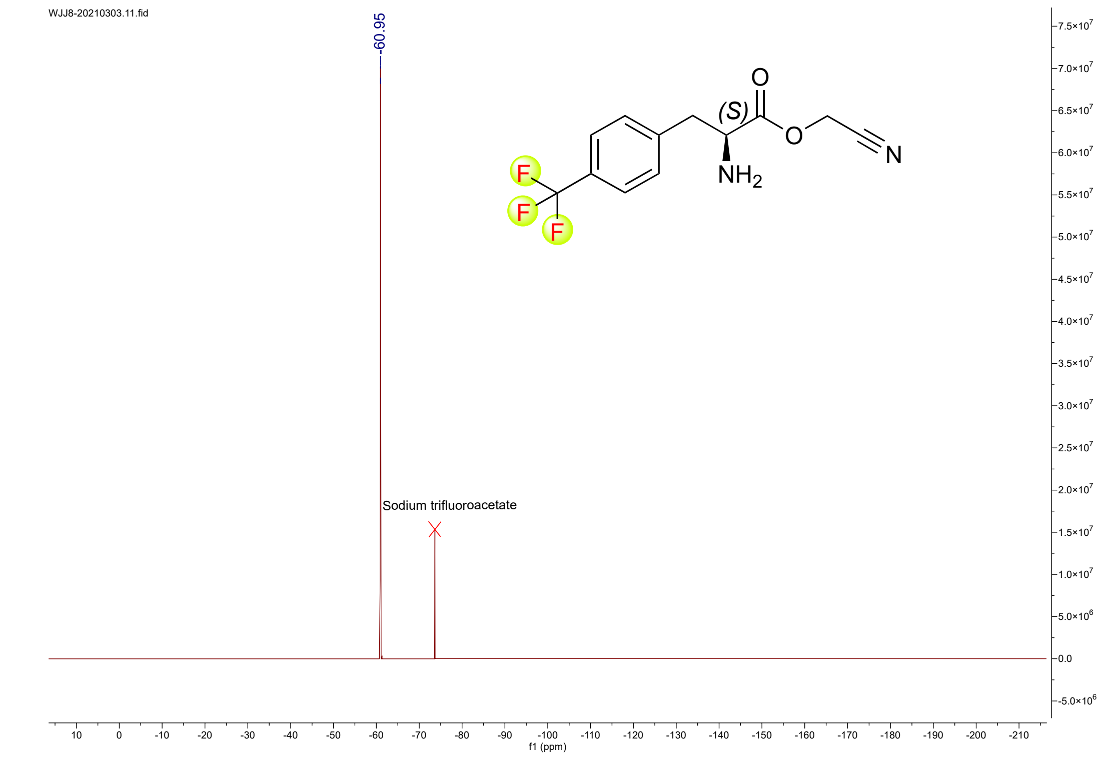

^{1}H NMR of 4-F-β-Phe-CME

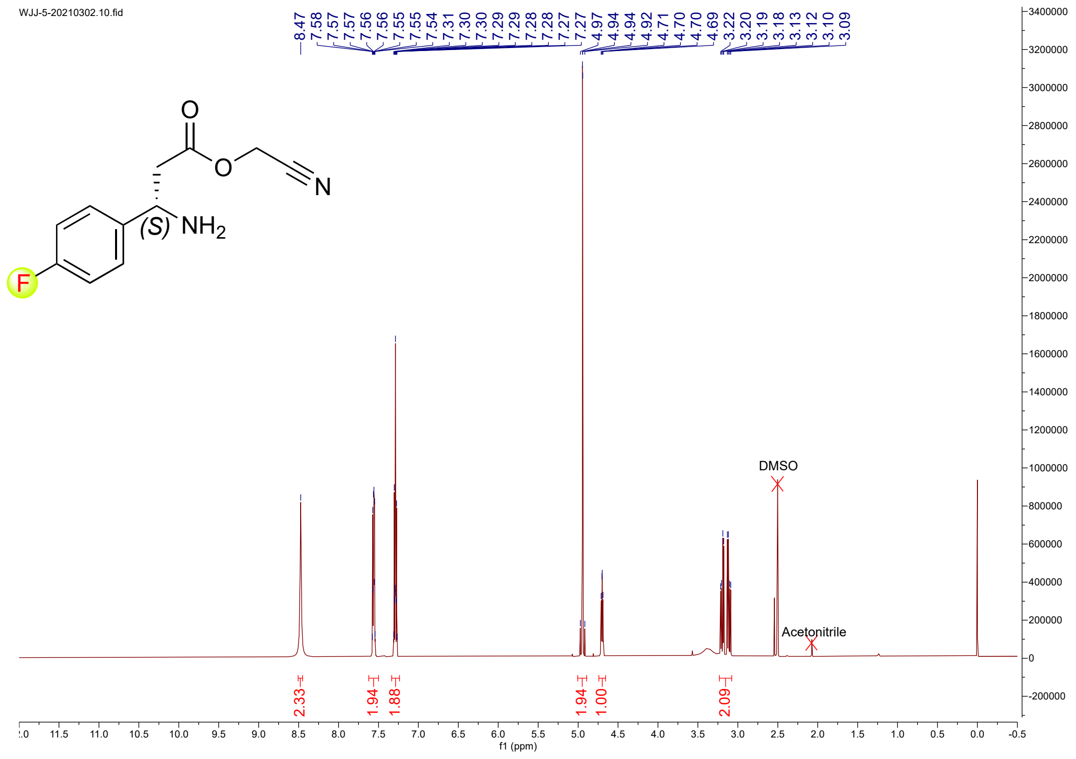

^{13}C NMR of 4-F-β-Phe-CME

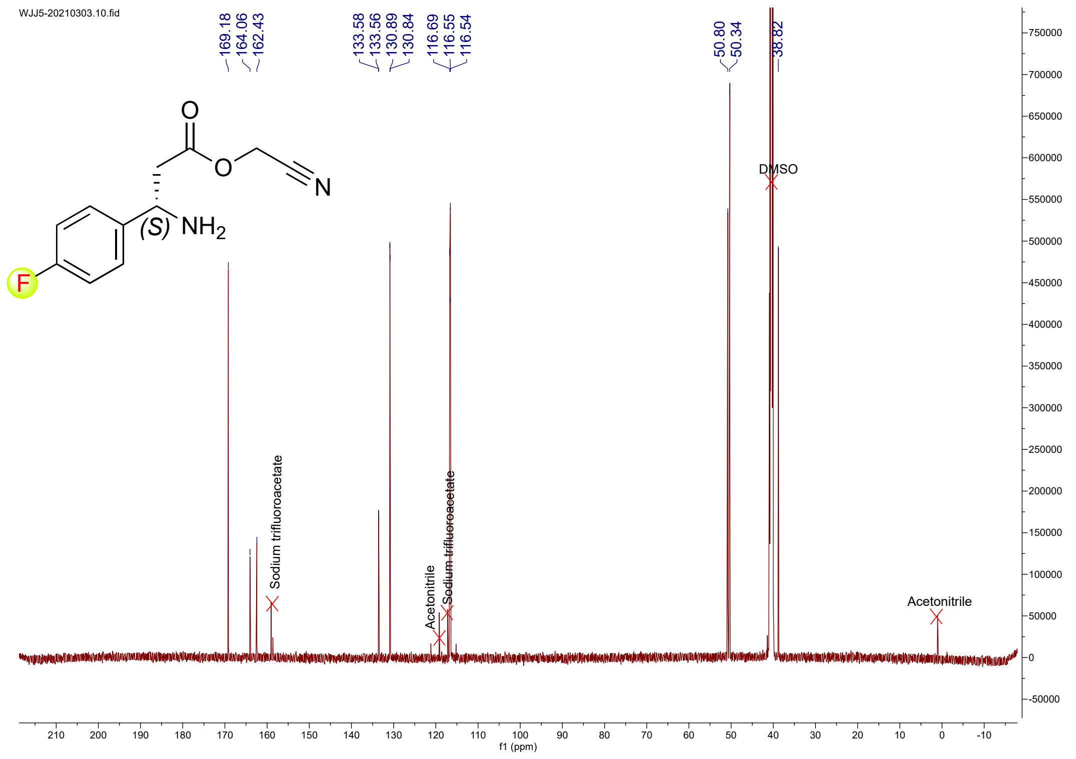

^{19}F NMR of 4-F-β-Phe-CME

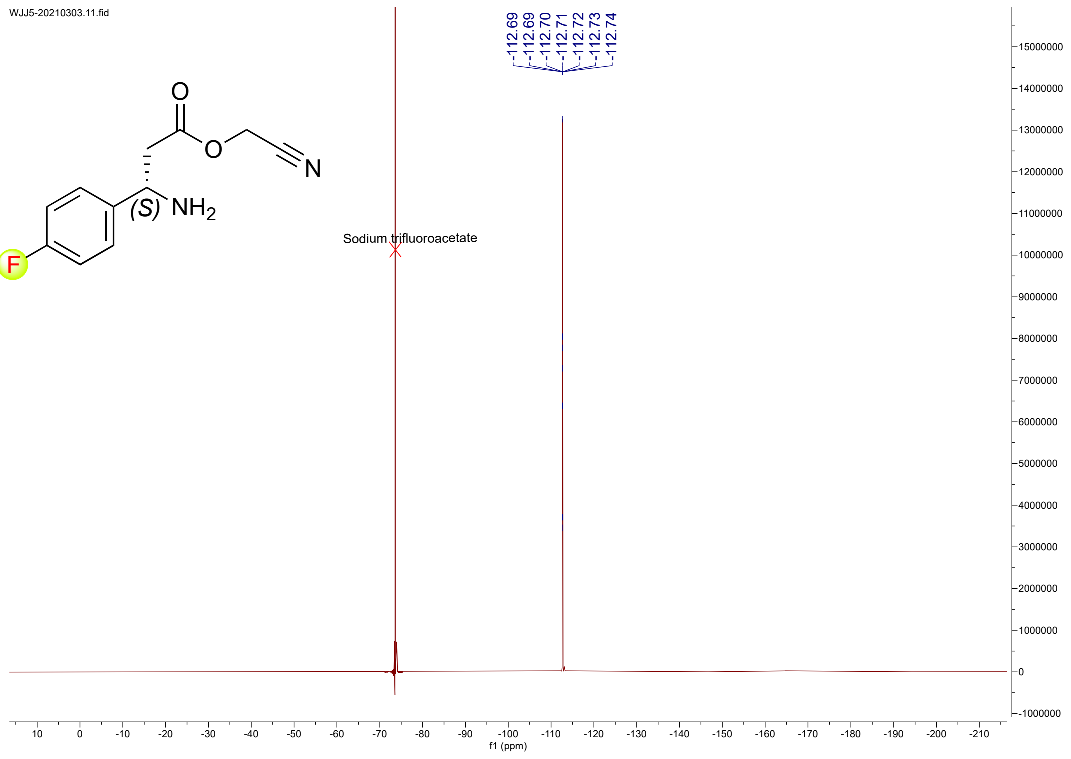

^{1}H NMR of 2F-[L]Pro-DBE

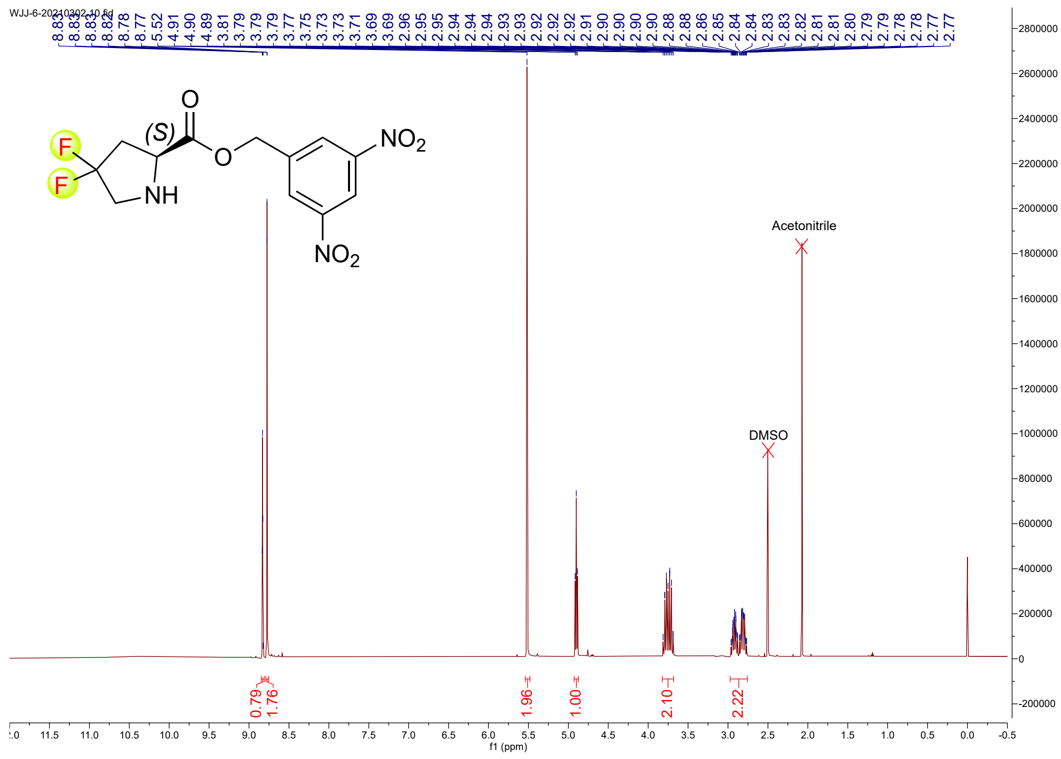

^{13}C NMR of 2F-^{L}Pro-DBE

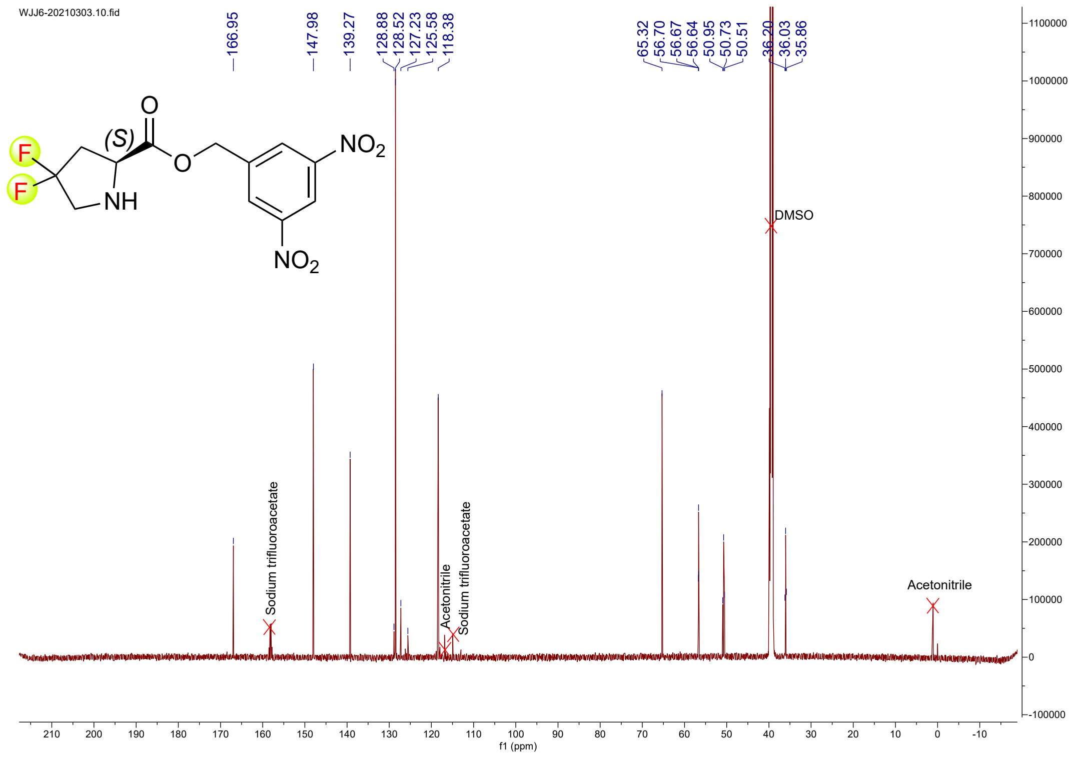

^{19}F NMR of 2F-^{L}Pro-DBE

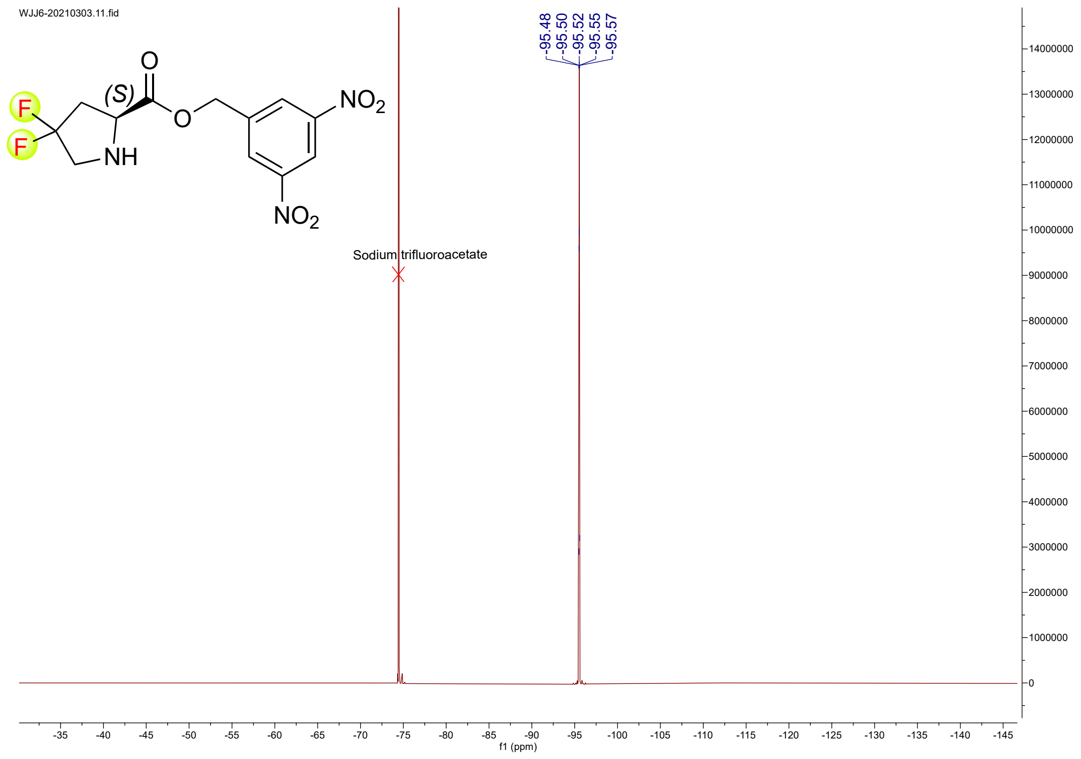

^{1}H NMR of cis-4-F-[L]Pro-DBE

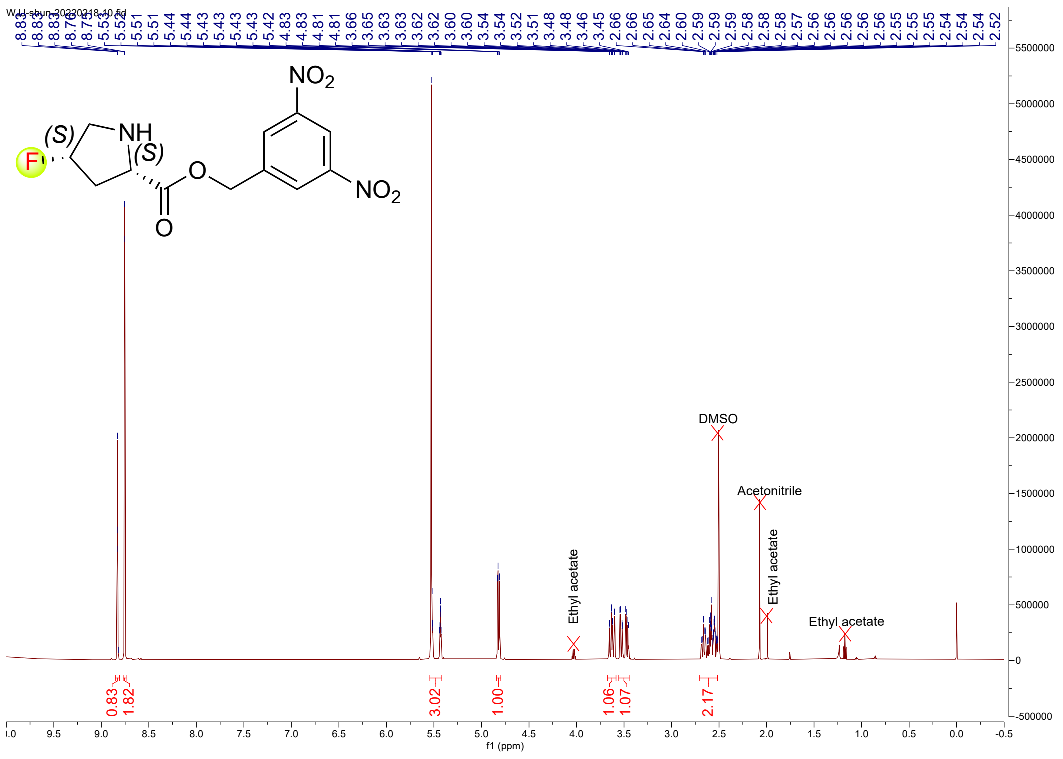

^{13}C NMR of cis-4-F-[L]Pro-DBE

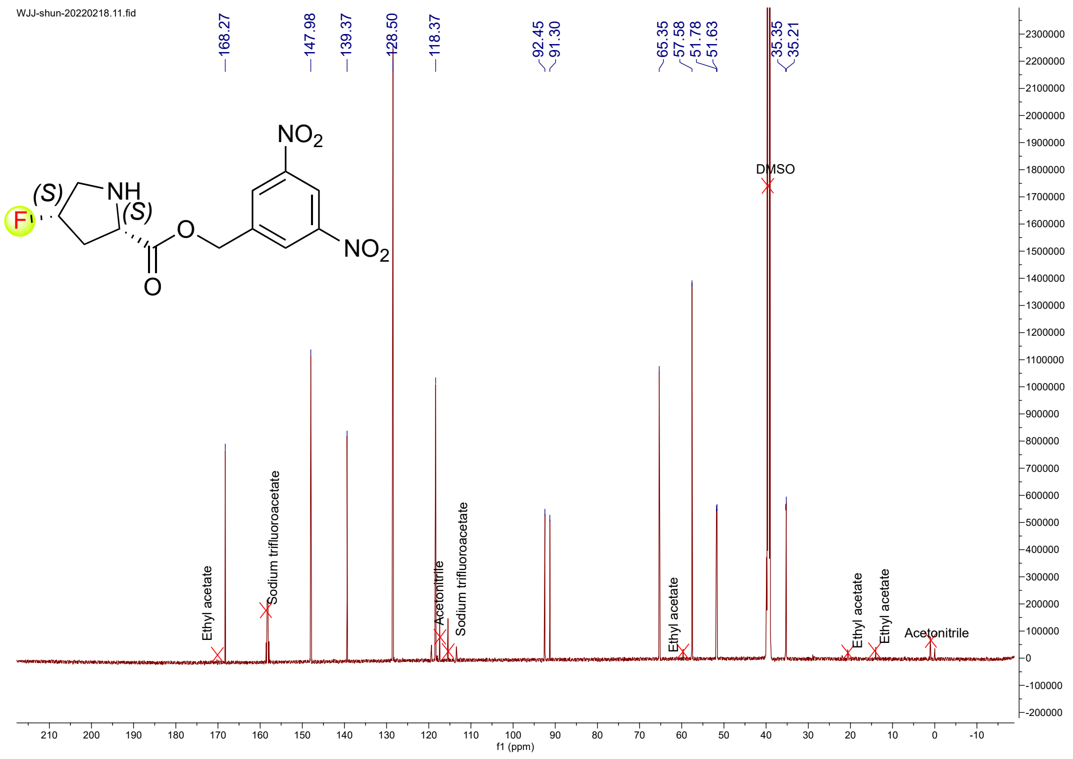

^{19}F NMR of cis-4-F-^{L}Pro-DBE

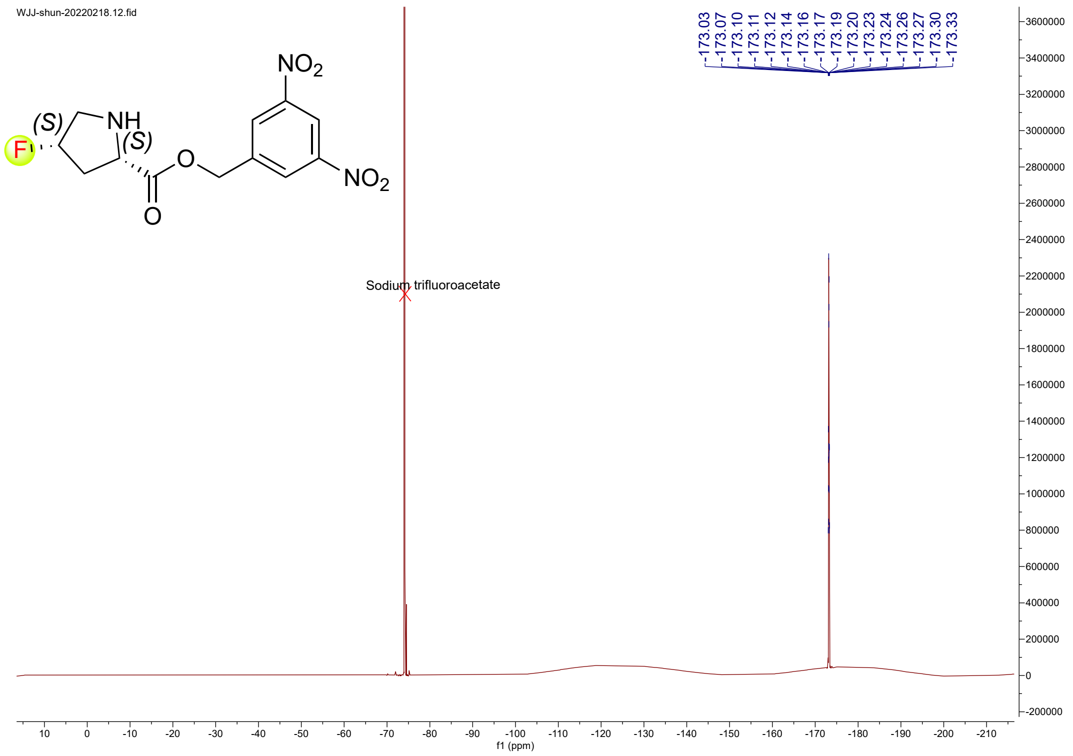

^{1}H NMR of trans-4-F-^{L}Pro-DBE

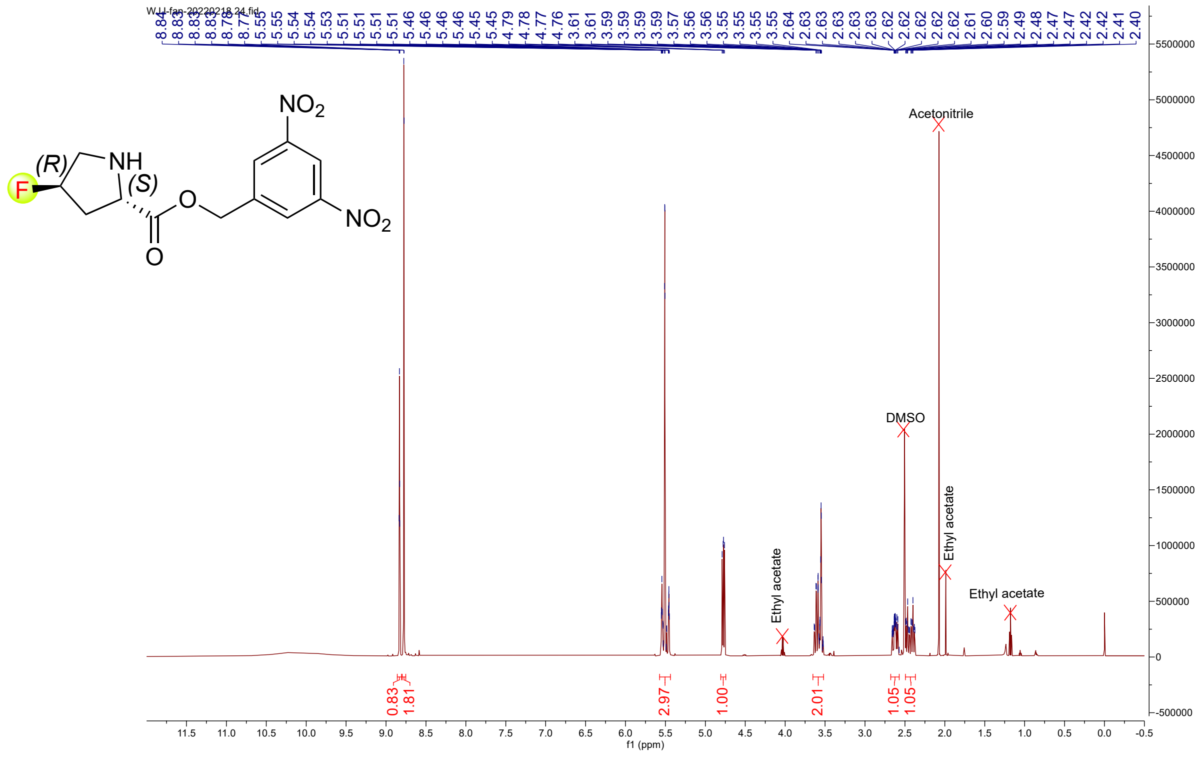

^{13}C NMR of trans-4-F-^{L}Pro-DBE

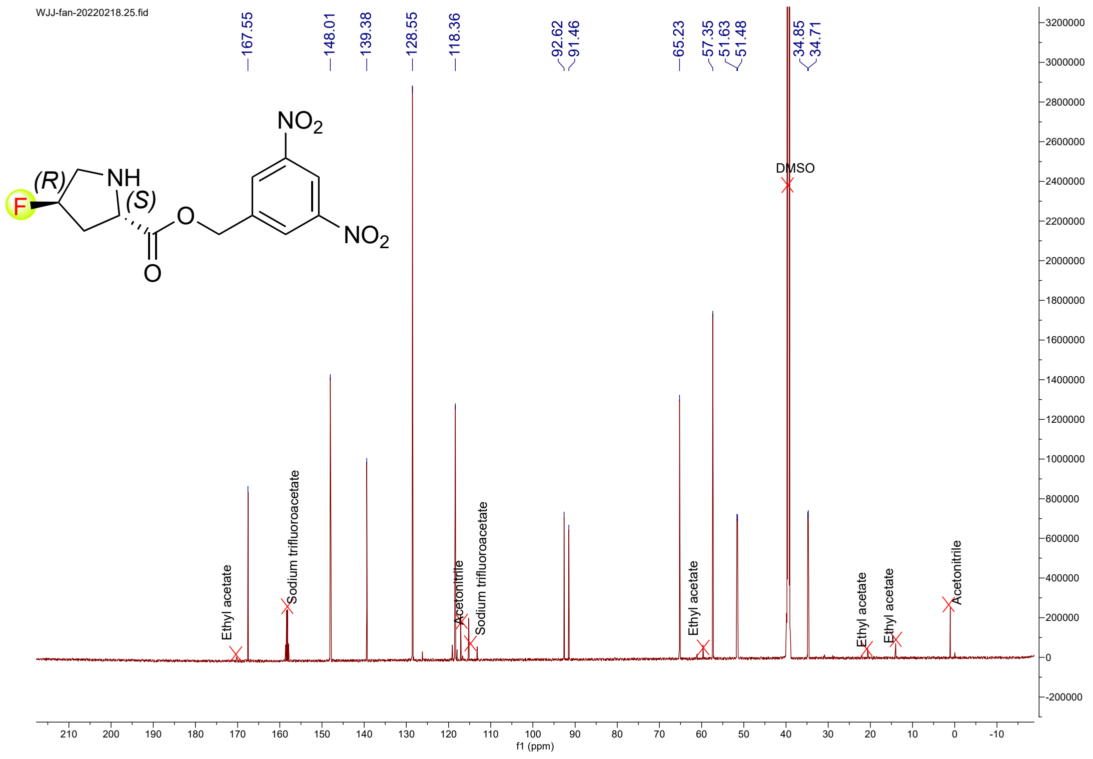

^{19}F NMR of trans-4-F-^{L}Pro-DBE

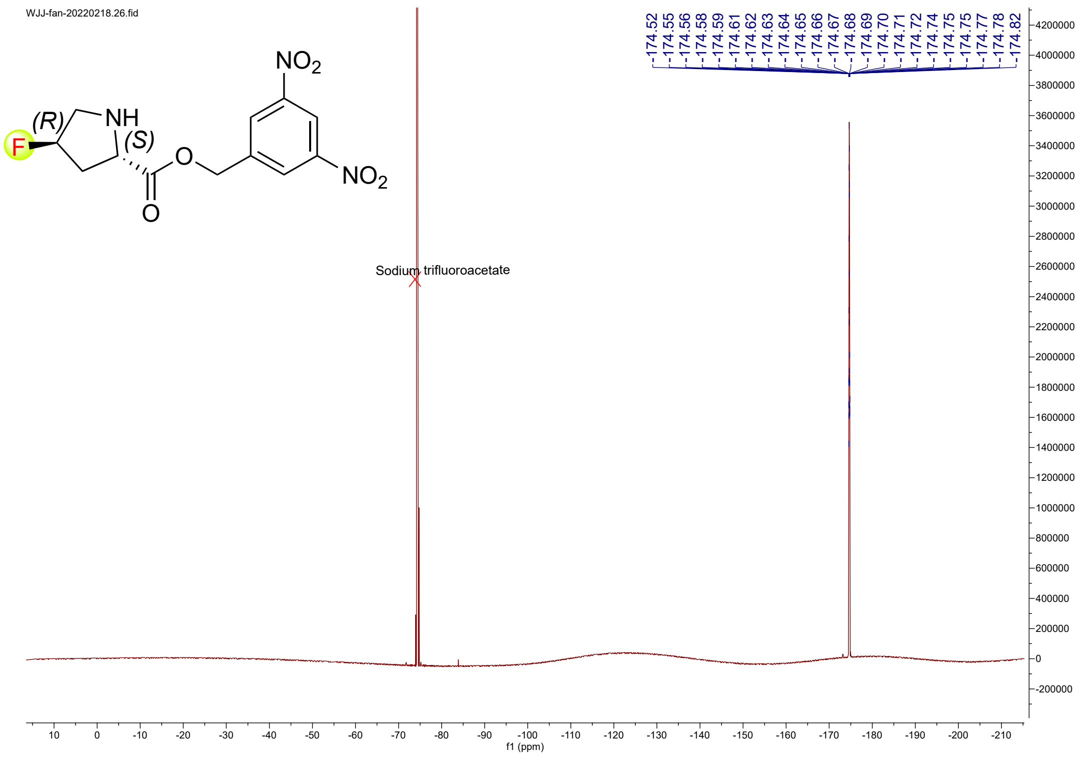

Characterization of synthesized macrocyclic peptides by ESI-MS and analytical HPLC (the calculated mass (Calcd.) and observed mass (Obsd.) for peptides are shown in each spectrum).

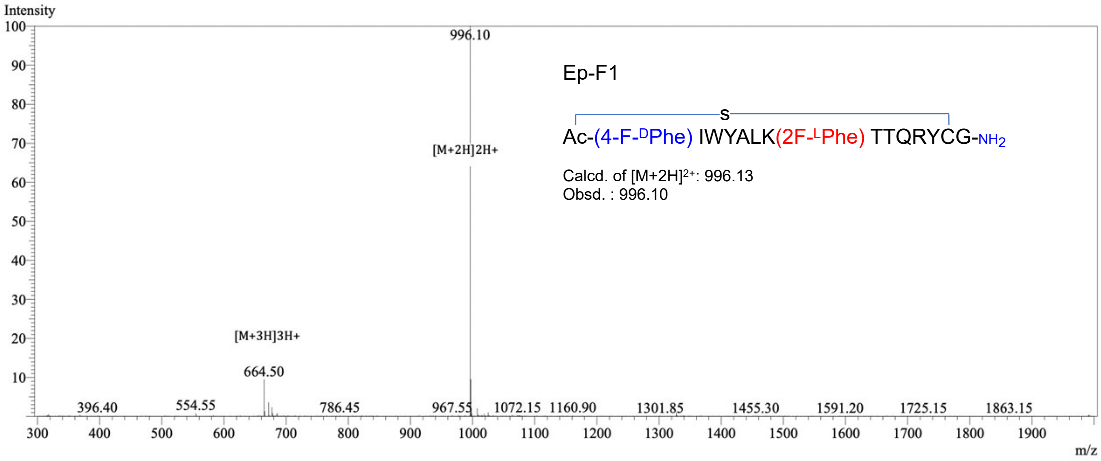

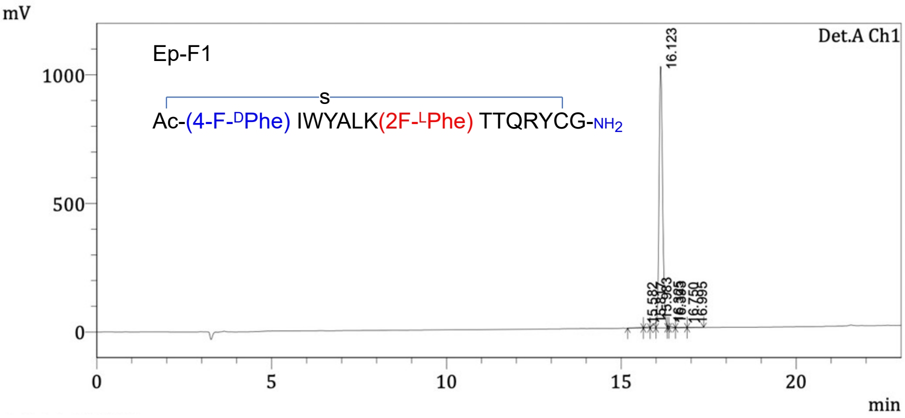

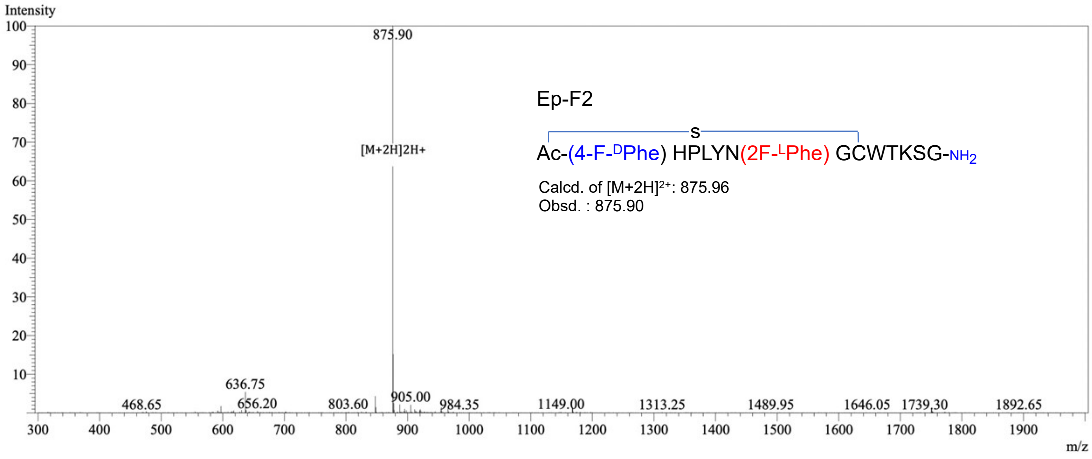

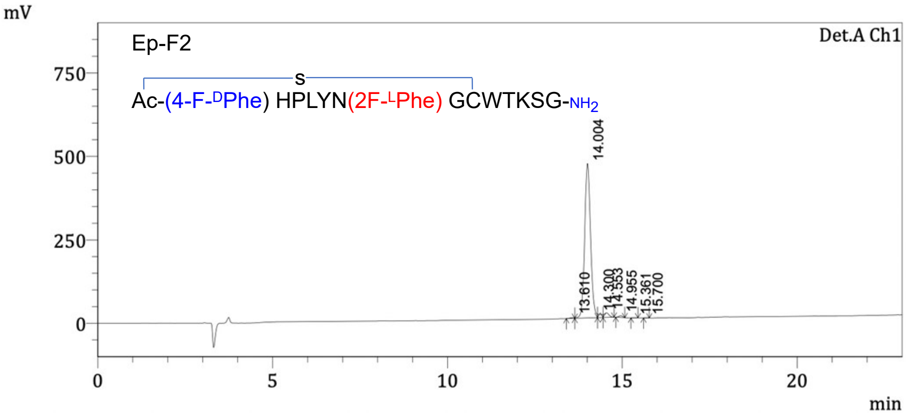
mV
Ep-F2
Det.A Ch1
S
Ac-(4-F-DPhe) HPLYN(2F-LPhe) GCWTKSG-NH2
750
500
250
0
14.004
13.610
14.300
14.553
14.955
15.361
15.700
0
5
10
15
20
min

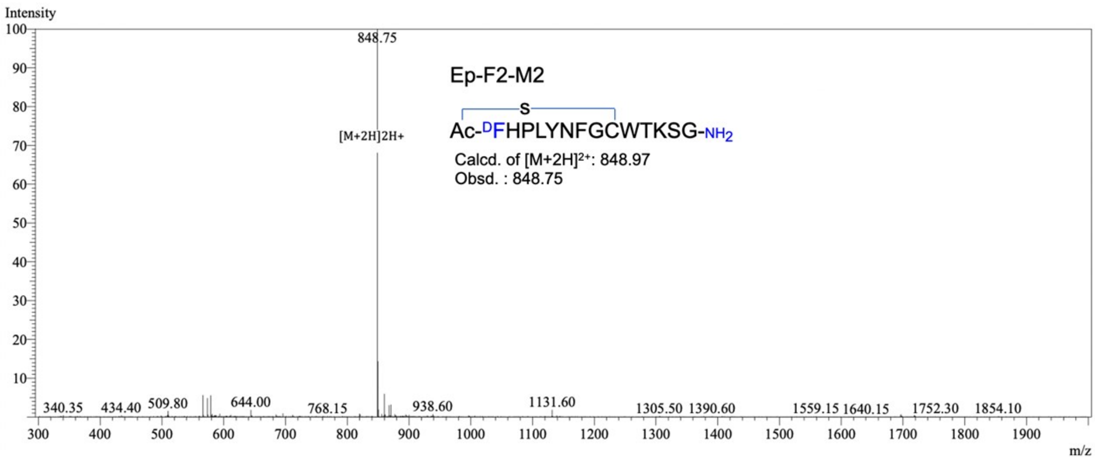
Intensity
848.75
[M+2H]2H+
Ep-F2-M2
S
Ac-DFHPLYNFGCWTKSG-NH2
Calcd. of [M+2H]2+: 848.97
Obsd. : 848.75
340.35
434.40
509.80
644.00
768.15
938.60
1131.60
1305.50
1390.60
1559.15
1640.15
1752.30
1854.10
m/z

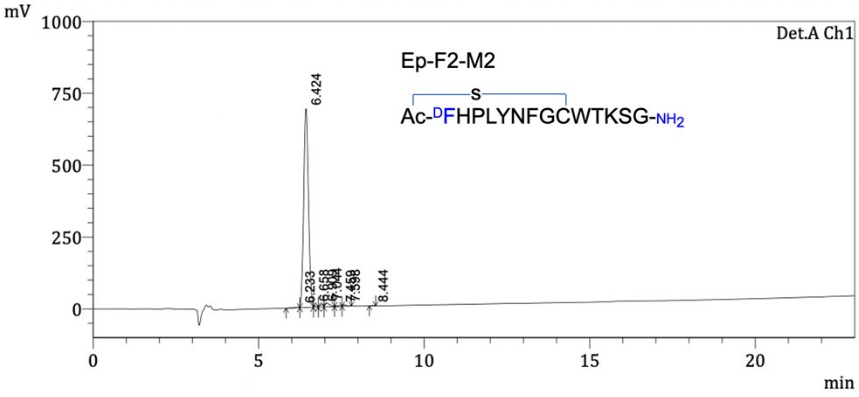
mV
1000
750
500
250
0
Det.A Ch1
Ep-F2-M2
S
Ac-DFHPLYNFGCWTKSG-NH2
6.424
6.233
6.658
7.459
7.598
8.444
0
5
10
15
20
min

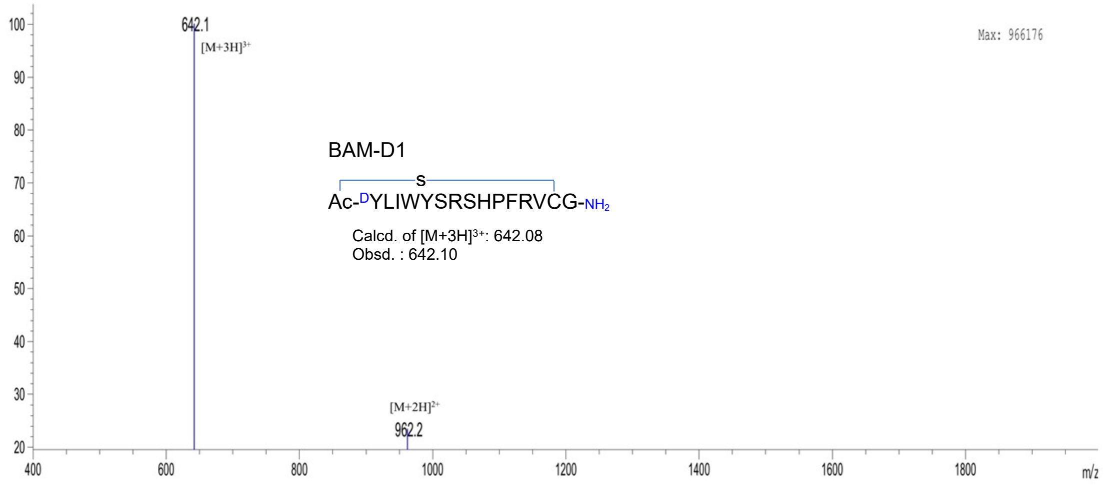

642.1
[M+3H]3+
Max: 966176
BAM-D1
S
Ac-DYLIWYSRSHPFRVCG-NH2
Calcd. of [M+3H]3+: 642.08
Obsd. : 642.10
[M+2H]2+
962.2
m/z

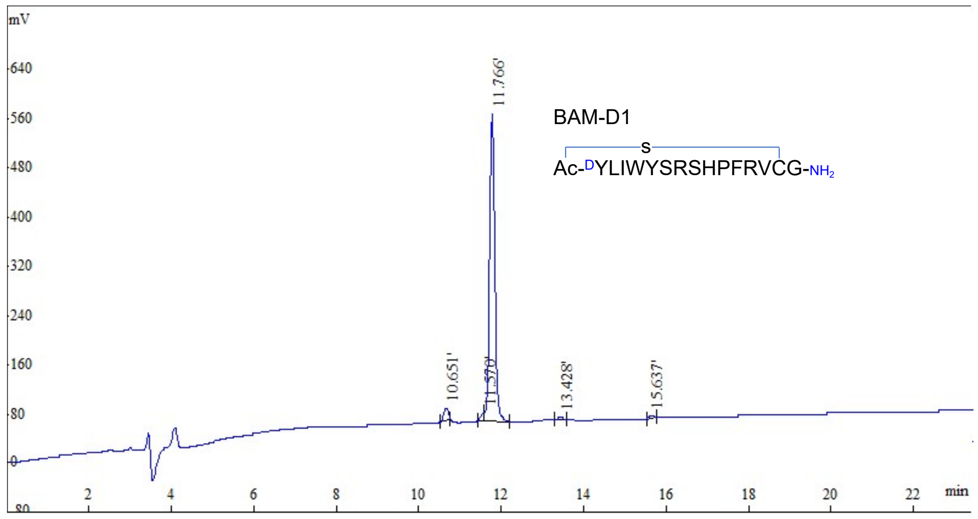

mV
11.766'
BAM-D1
S
Ac-DYLIWYSRSHPFRVCG-NH2
10.651'
13.428'
15.637'
min

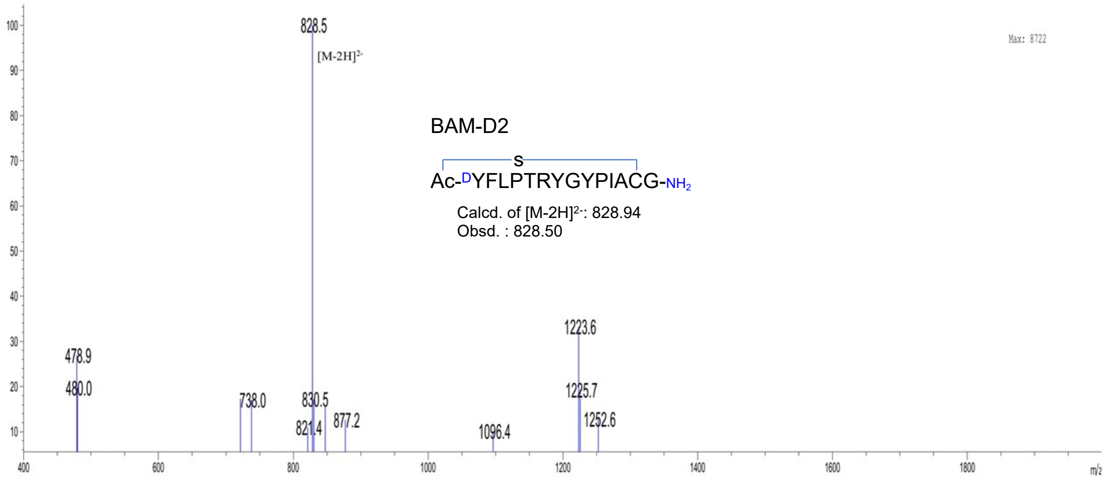

828.5
[M-2H]2-
Max: 8722
BAM-D2
S
Ac-DYFLPTRYGYPIACG-NH2
Calcd. of [M-2H]2-: 828.94
Obsd. : 828.50
478.9
480.0
738.0
830.5
821.4
877.2
1096.4
1223.6
1225.7
1252.6
m/z

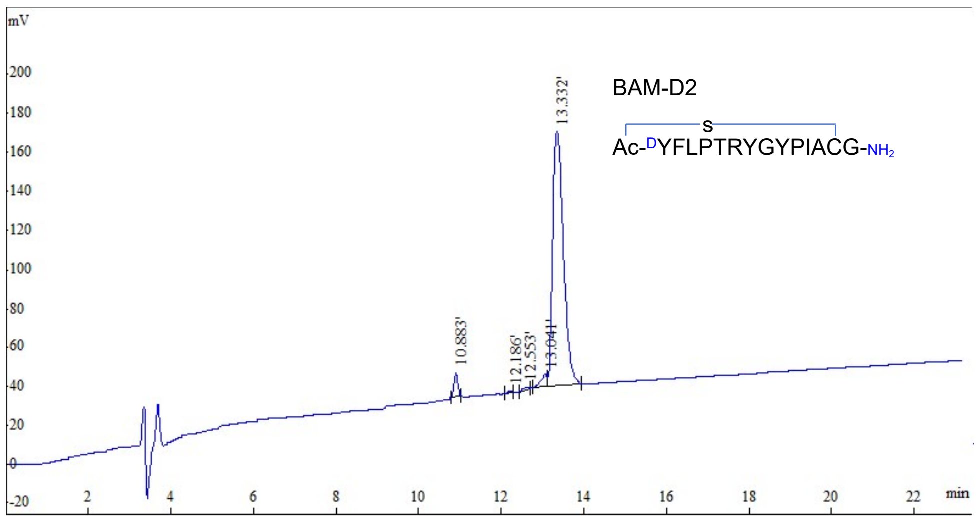
mV
13.332'
10.883'
12.186'
12.553'
13.041'
min
BAM-D2
Ac-DYFLPTRYGYPIACG-NH2

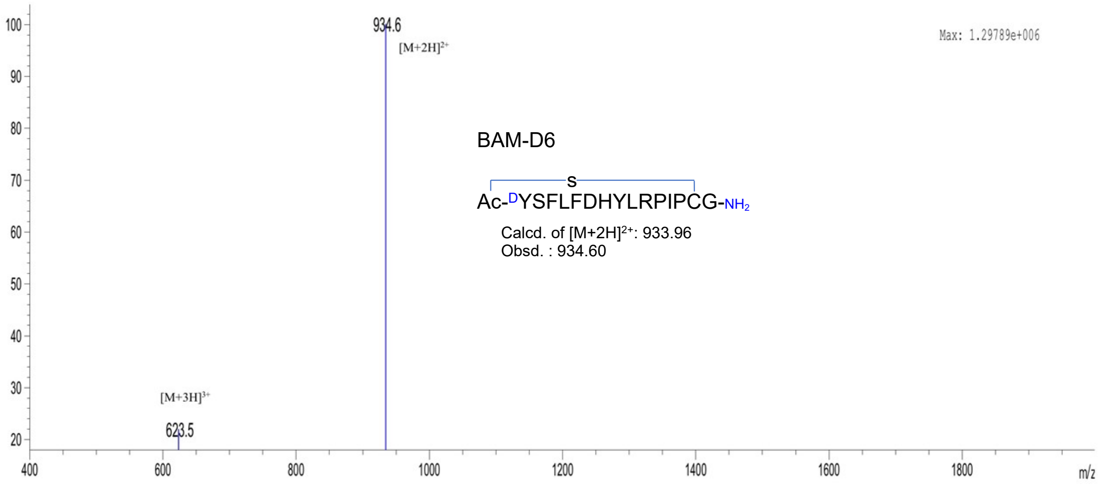
934.6
[M+2H]2+
Max: 1.29789e+006
BAM-D6
Ac-DYSFLFDHYLRPIPCG-NH2
Calcd. of [M+2H]2+: 933.96
Obsd. : 934.60
[M+3H]3+
623.5
m/z

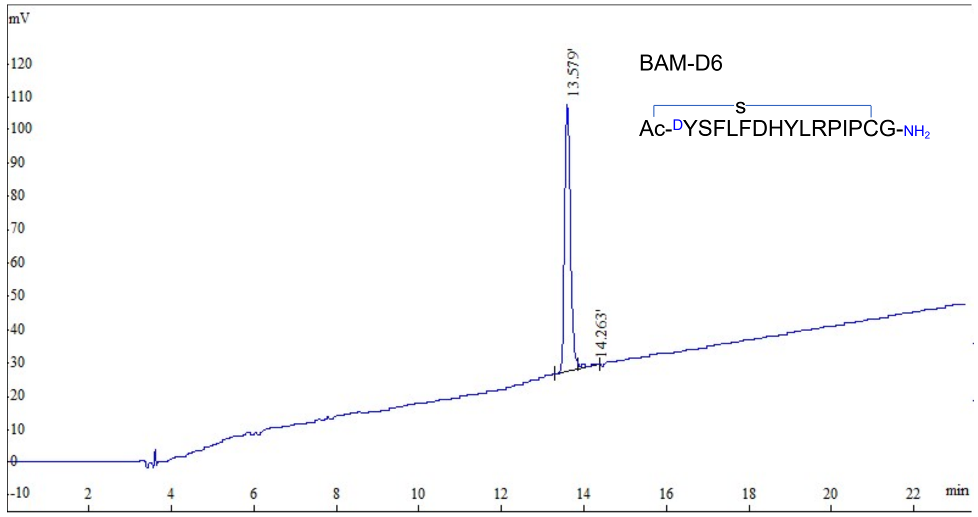
mV
13.579'
14.263'
min
BAM-D6
Ac-DYSFLFDHYLRPIPCG-NH2

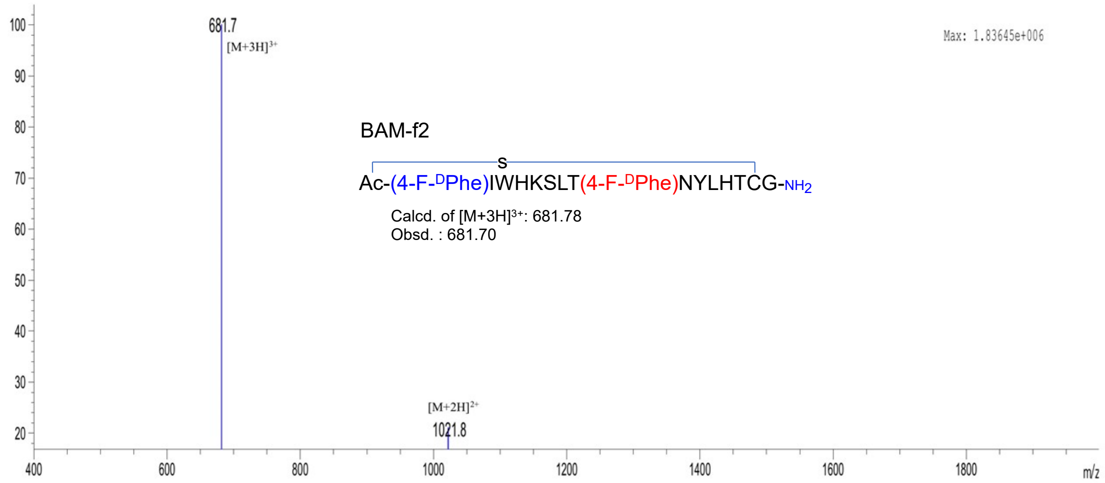
681.7
[M+3H]$^{3+}$
Max: 1.83645e+006
BAM-f2
S
Ac-(4-F-^{D}Phe)IWHKSLT(4-F-^{D}Phe)NYLHTCG-NH_2
Calcd. of [M+3H]$^{3+}$: 681.78
Obsd. : 681.70
[M+2H]$^{2+}$
1021.8
m/z

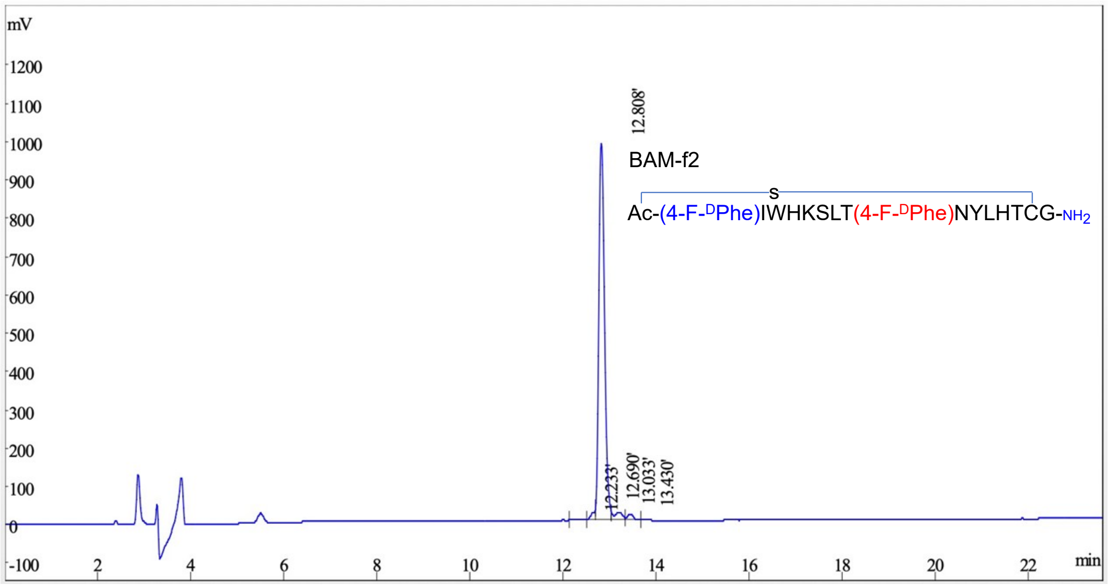
mV
12.808'
BAM-f2
S
Ac-(4-F-^{D}Phe)IWHKSLT(4-F-^{D}Phe)NYLHTCG-NH_2
12.233'
12.690'
13.033'
13.430'
min

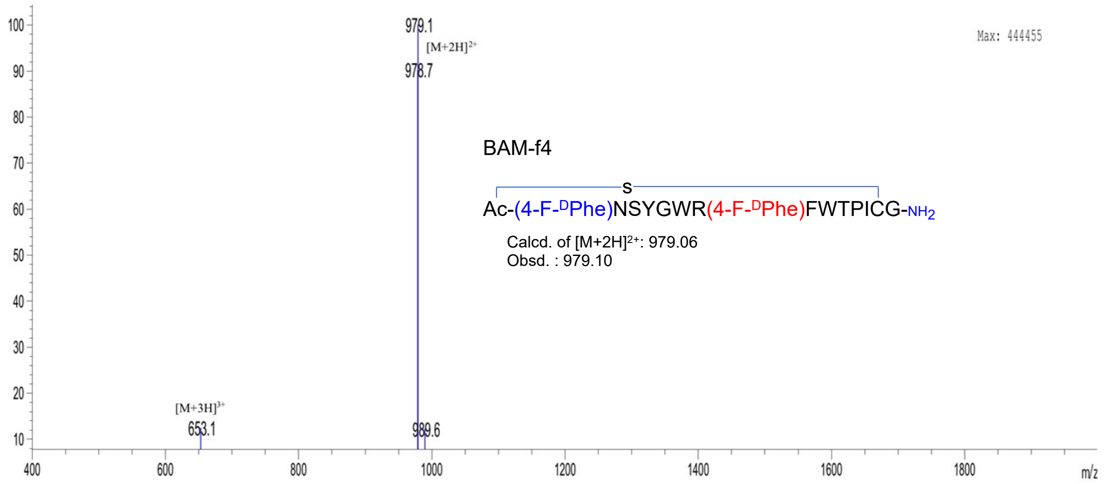
979.1
[M+2H]$^{2+}$
978.7
Max: 444455
BAM-f4
S
Ac-(4-F-^{D}Phe)NSYGWR(4-F-^{D}Phe)FWTPICG-NH_2
Calcd. of [M+2H]$^{2+}$: 979.06
Obsd. : 979.10
[M+3H]$^{3+}$
653.1
989.6
m/z

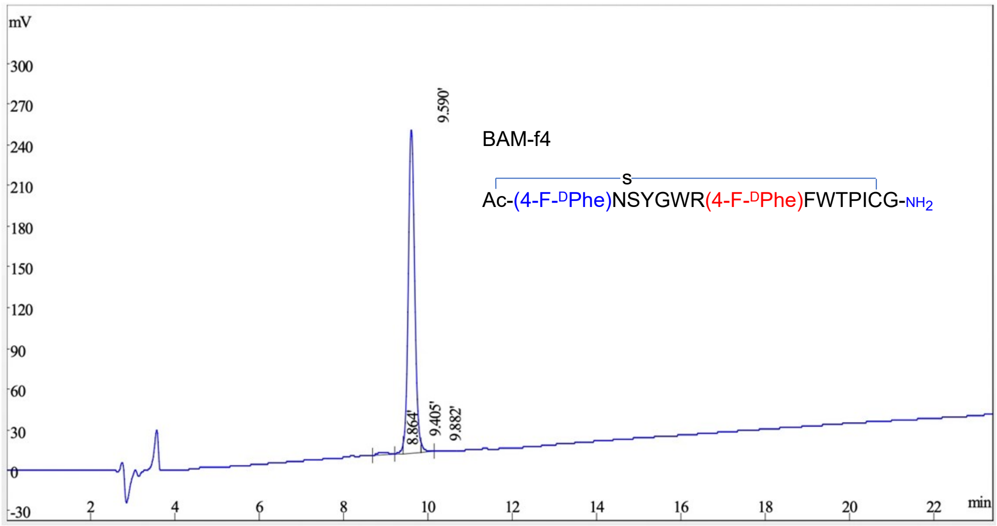
mV
300
270
240
210
180
150
120
90
60
30
0
-30
9.590'
8.864'
9.405'
9.882'
BAM-f4
S
Ac-(4-F-DPhe)NSYGWR(4-F-DPhe)FWTPICG-NH2
2
4
6
8
10
12
14
16
18
20
22
min

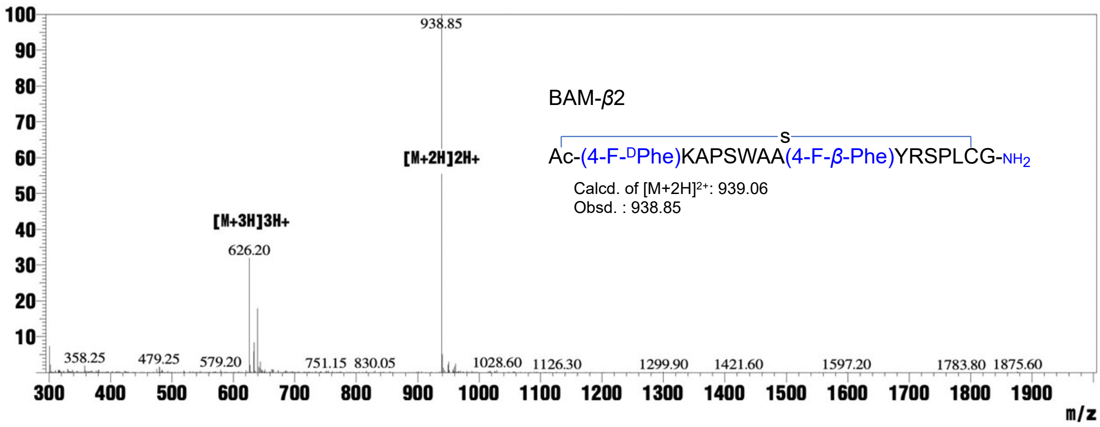
100
90
80
70
60
50
40
30
20
10
938.85
[M+2H]2H+
[M+3H]3H+
626.20
358.25
479.25
579.20
751.15
830.05
1028.60
1126.30
1299.90
1421.60
1597.20
1783.80
1875.60
BAM-β2
S
Ac-(4-F-DPhe)KAPSWAA(4-F-β-Phe)YRSPLCG-NH2
Calcd. of [M+2H]2+: 939.06
Obsd. : 938.85
300
400
500
600
700
800
900
1000
1100
1200
1300
1400
1500
1600
1700
1800
1900
m/z

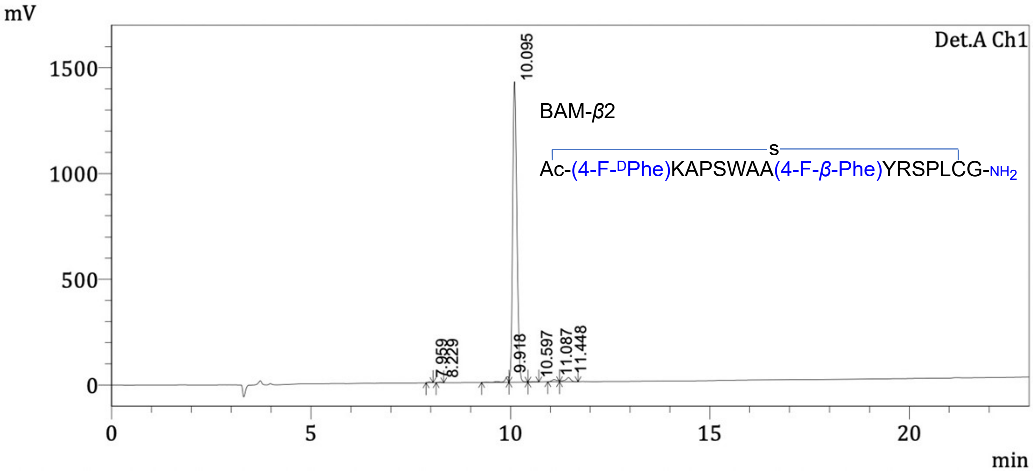
mV
Det.A Ch1
1500
1000
500
0
10.095
BAM-β2
S
Ac-(4-F-DPhe)KAPSWAA(4-F-β-Phe)YRSPLCG-NH2
7.959
8.229
9.918
10.597
11.087
11.448
0
5
10
15
20
min

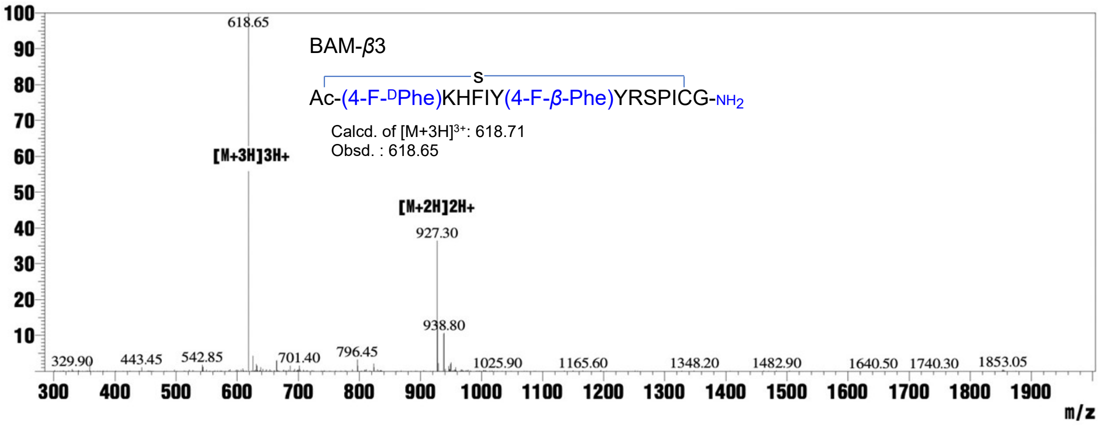
BAM-β3
Ac-(4-F-DPhe)KHFIY(4-F-β-Phe)YRSPICG-NH2
Calcd. of [M+3H]3+: 618.71
Obsd. : 618.65
[M+3H]3H+
618.65
[M+2H]2H+
927.30
938.80
m/z

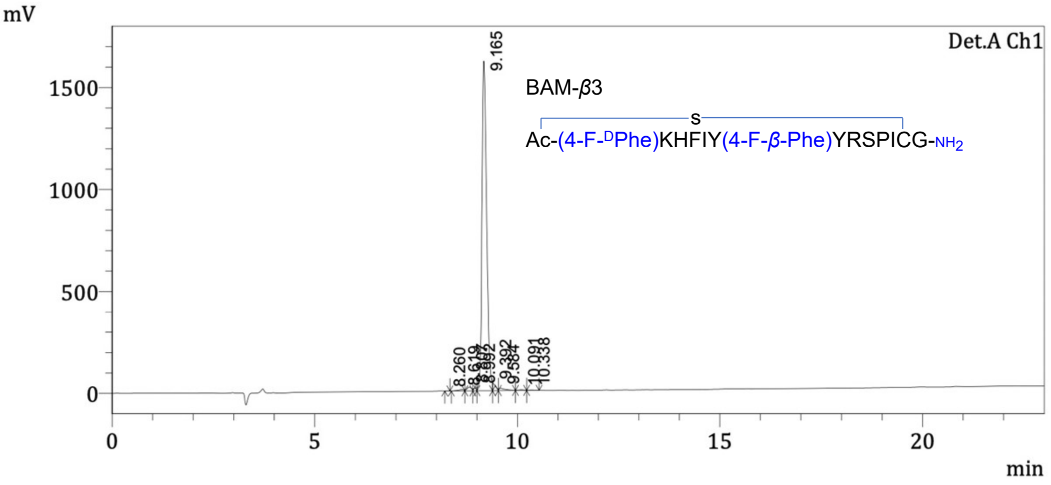
mV
Det.A Ch1
9.165
BAM-β3
Ac-(4-F-DPhe)KHFIY(4-F-β-Phe)YRSPICG-NH2
min

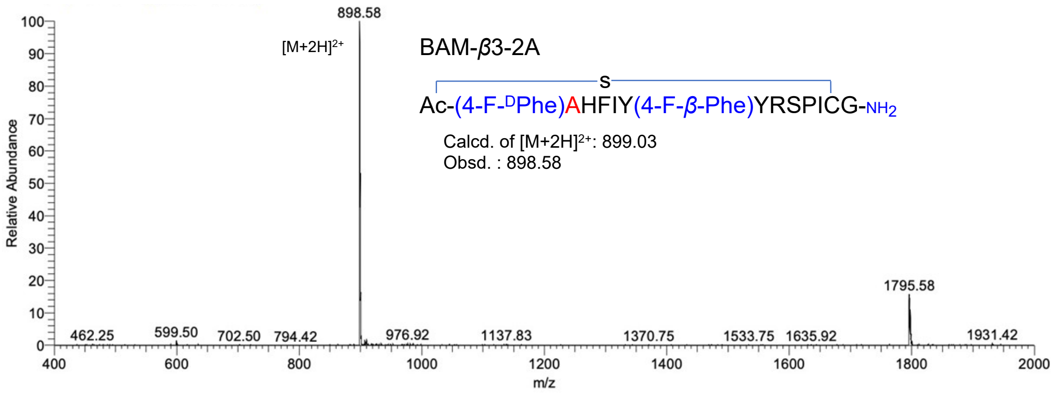
898.58
[M+2H]2+
BAM-β3-2A
Ac-(4-F-DPhe)AHFIY(4-F-β-Phe)YRSPICG-NH2
Calcd. of [M+2H]2+: 899.03
Obsd. : 898.58
1795.58
Relative Abundance
m/z

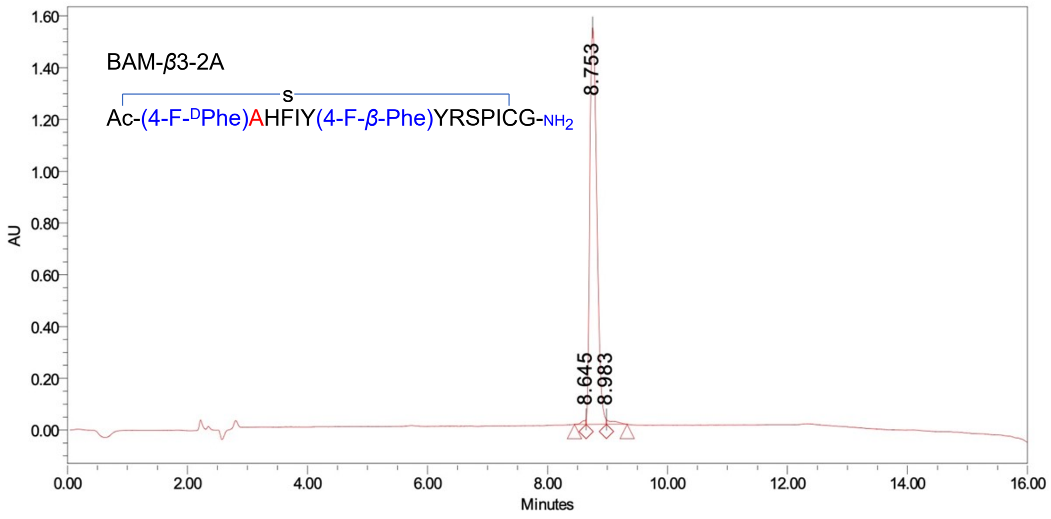
BAM-β3-2A
Ac-(4-F-DPhe)AHFIY(4-F-β-Phe)YRSPICG-NH2
S
8.753
8.645
8.983
AU
Minutes

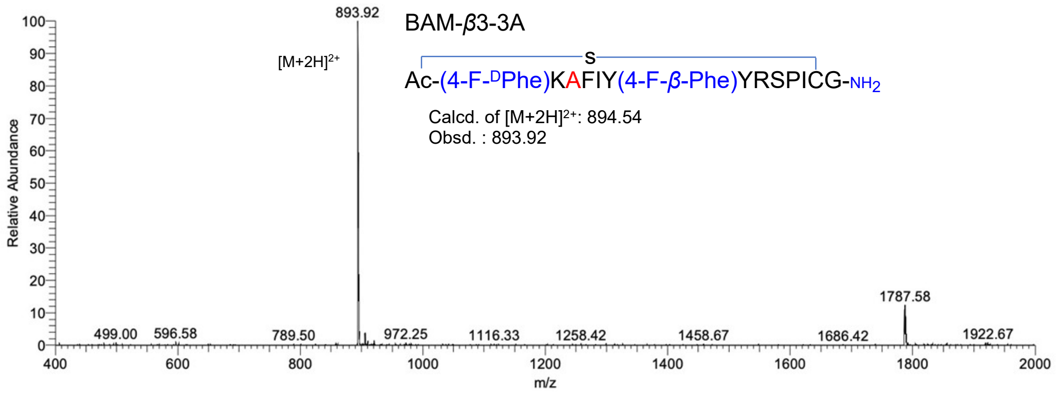
893.92
[M+2H]2+
BAM-β3-3A
Ac-(4-F-DPhe)KAFIY(4-F-β-Phe)YRSPICG-NH2
S
Calcd. of [M+2H]2+: 894.54
Obsd. : 893.92
1787.58
499.00
596.58
789.50
972.25
1116.33
1258.42
1458.67
1686.42
1922.67
Relative Abundance
m/z

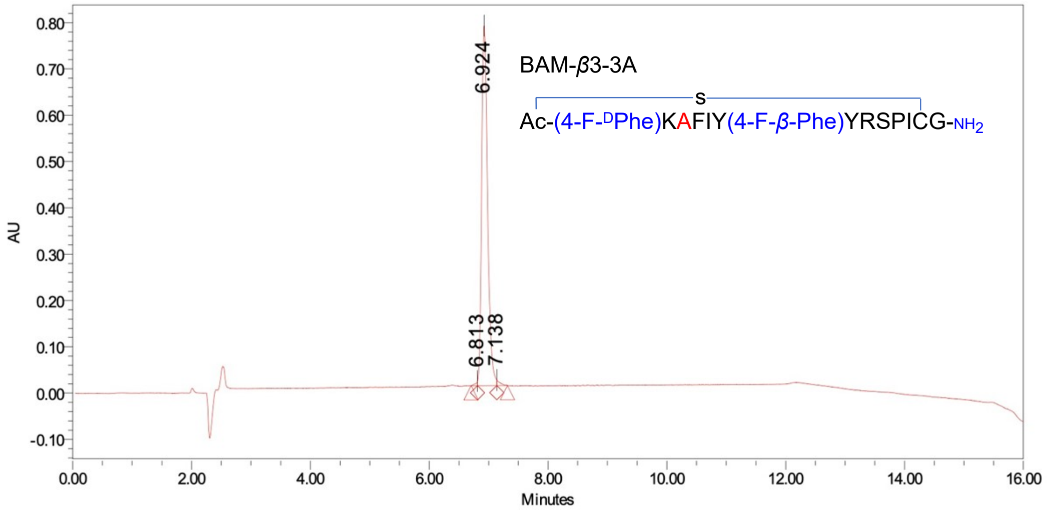
BAM-β3-3A
Ac-(4-F-DPhe)KAFIY(4-F-β-Phe)YRSPICG-NH2
S
6.924
6.813
7.138
AU
Minutes

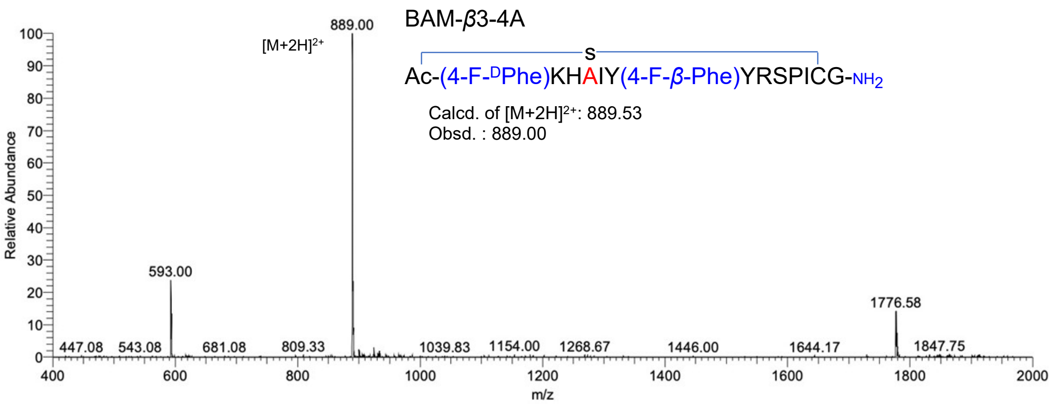

BAM-β3-4A
Ac-(4-F-DPhe)KHAIY(4-F-β-Phe)YRSPICG-NH2
S
Calcd. of [M+2H]2+: 889.53
Obsd. : 889.00
[M+2H]2+
889.00
593.00
1776.58
447.08
543.08
681.08
809.33
1039.83
1154.00
1268.67
1446.00
1644.17
1847.75
Relative Abundance
m/z

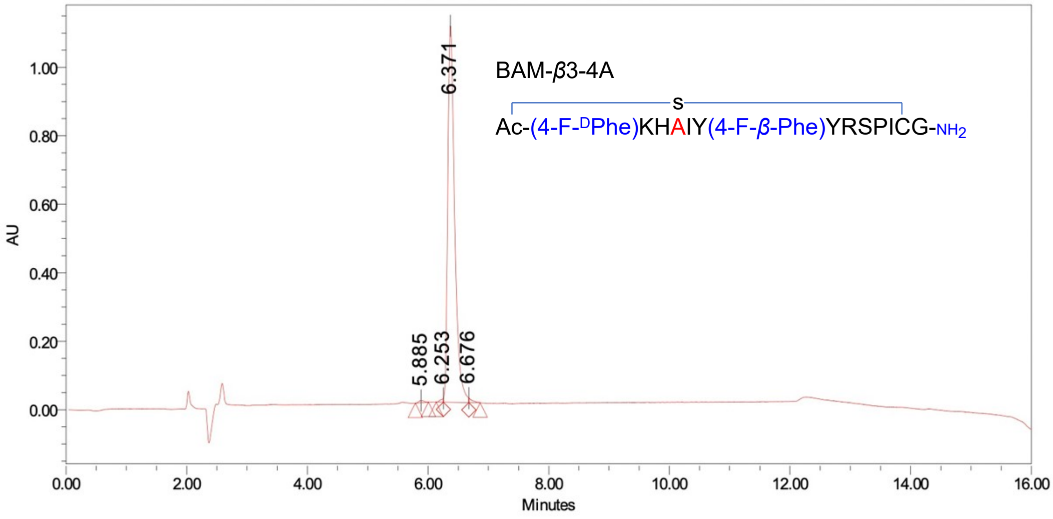

BAM-β3-4A
Ac-(4-F-DPhe)KHAIY(4-F-β-Phe)YRSPICG-NH2
S
6.371
5.885
6.253
6.676
AU
Minutes

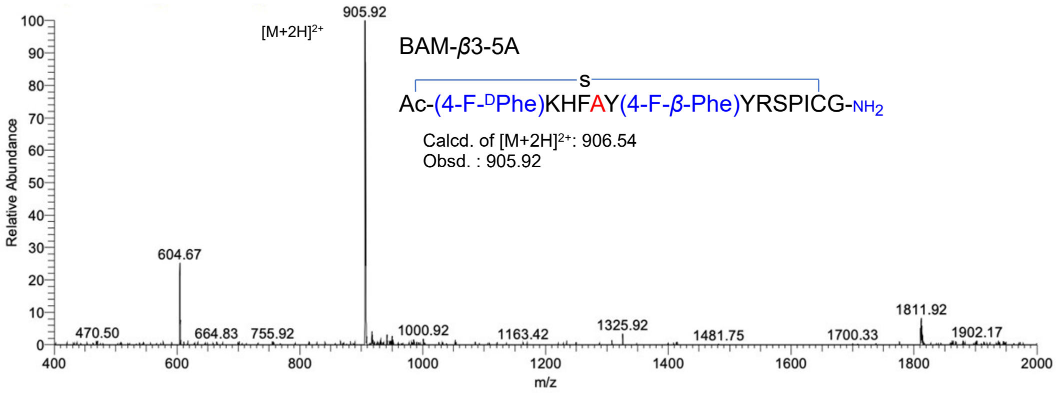

BAM-β3-5A
Ac-(4-F-DPhe)KHFAY(4-F-β-Phe)YRSPICG-NH2
S
Calcd. of [M+2H]2+: 906.54
Obsd. : 905.92
[M+2H]2+
905.92
604.67
1811.92
470.50
664.83
755.92
1000.92
1163.42
1325.92
1481.75
1700.33
1902.17
Relative Abundance
m/z

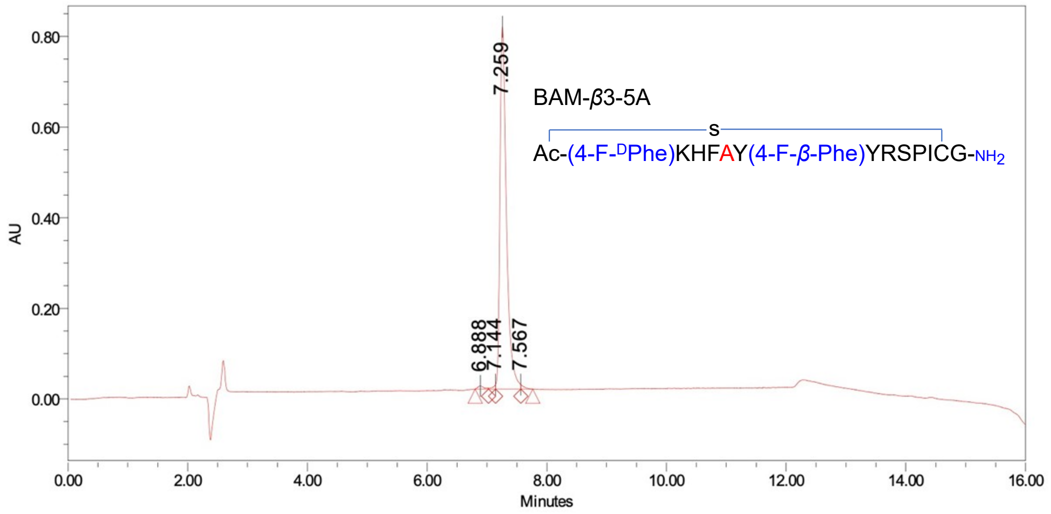
BAM-β3-5A
S
Ac-(4-F-DPhe)KHFAY(4-F-β-Phe)YRSPICG-NH2
7.259
6.888
7.144
7.567
AU
Minutes

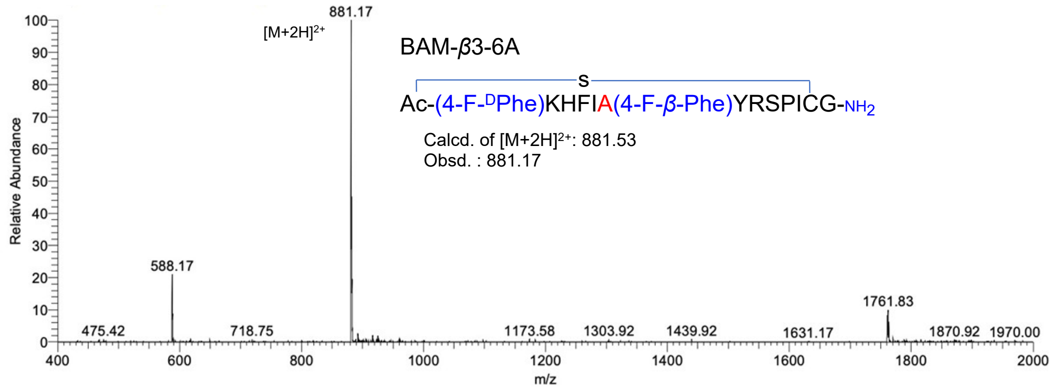
[M+2H]2+
881.17
BAM-β3-6A
S
Ac-(4-F-DPhe)KHFIA(4-F-β-Phe)YRSPICG-NH2
Calcd. of [M+2H]2+: 881.53
Obsd. : 881.17
588.17
475.42
718.75
1173.58
1303.92
1439.92
1631.17
1761.83
1870.92
1970.00
Relative Abundance
m/z

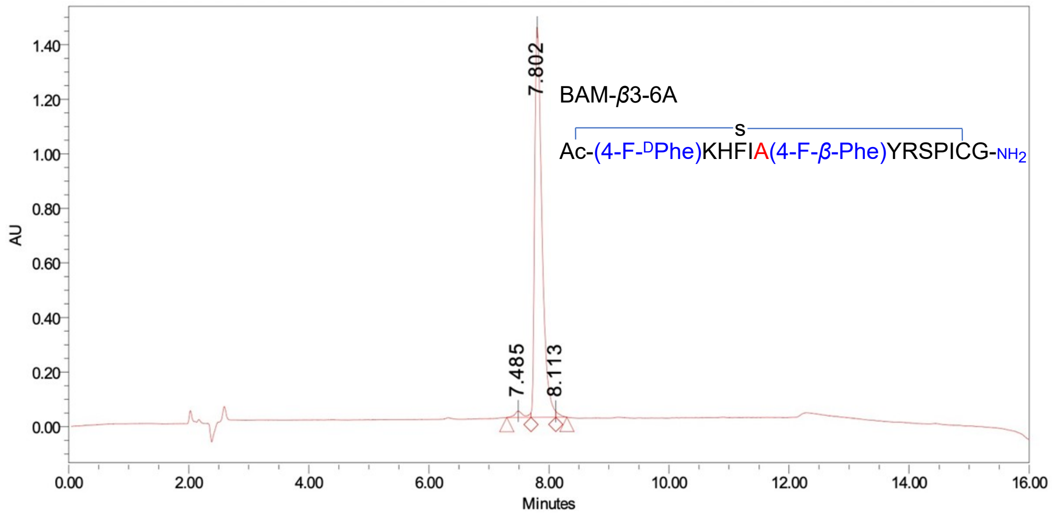
7.802
BAM-β3-6A
S
Ac-(4-F-DPhe)KHFIA(4-F-β-Phe)YRSPICG-NH2
7.485
8.113
AU
Minutes

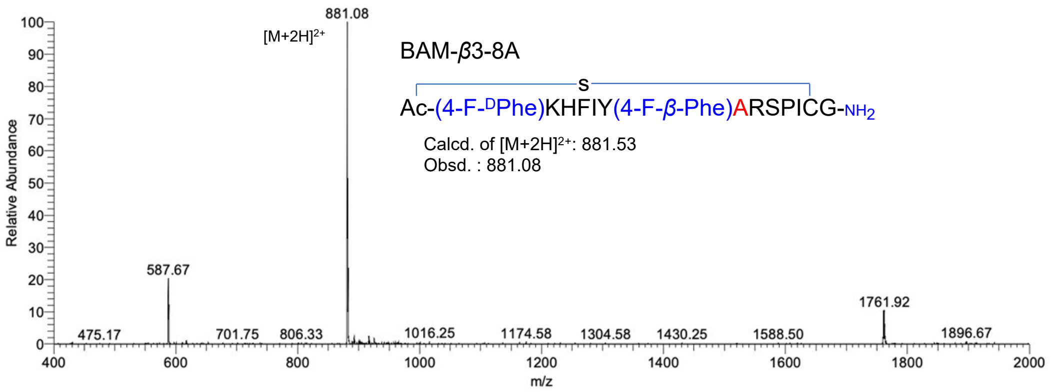
881.08
[M+2H]2+
BAM-β3-8A
Ac-(4-F-DPhe)KHFIY(4-F-β-Phe)ARSPICG-NH2
Calcd. of [M+2H]2+: 881.53
Obsd. : 881.08
587.67
1761.92
Relative Abundance
m/z

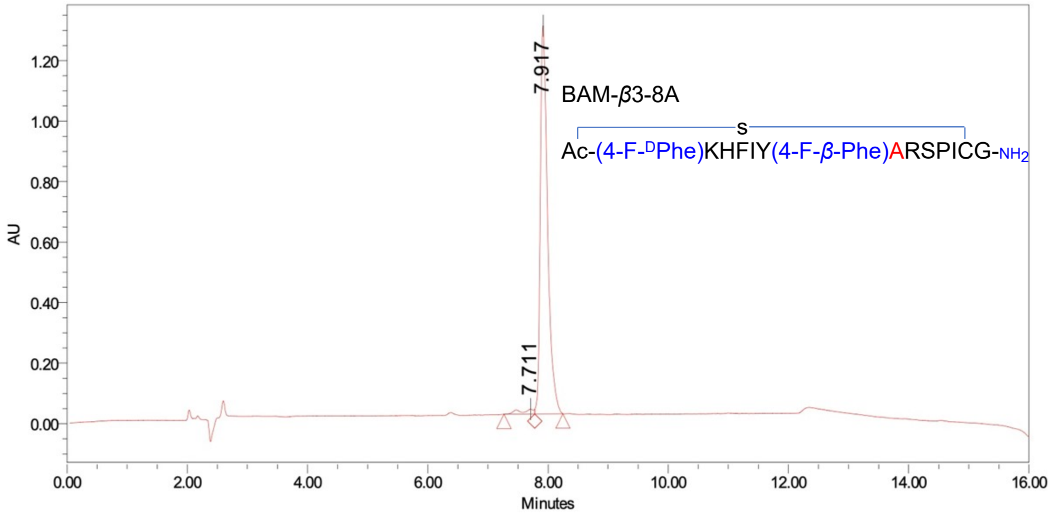
7.917
7.711
BAM-β3-8A
Ac-(4-F-DPhe)KHFIY(4-F-β-Phe)ARSPICG-NH2
AU
Minutes

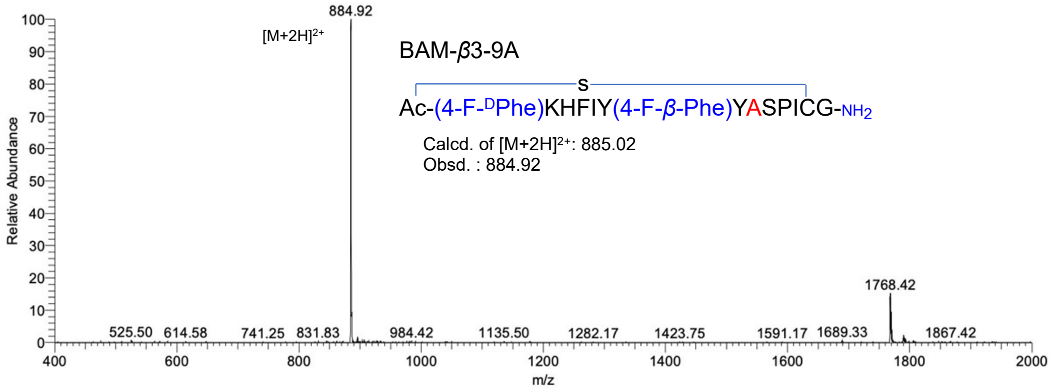
884.92
[M+2H]2+
BAM-β3-9A
Ac-(4-F-DPhe)KHFIY(4-F-β-Phe)YASPICG-NH2
Calcd. of [M+2H]2+: 885.02
Obsd. : 884.92
1768.42
Relative Abundance
m/z

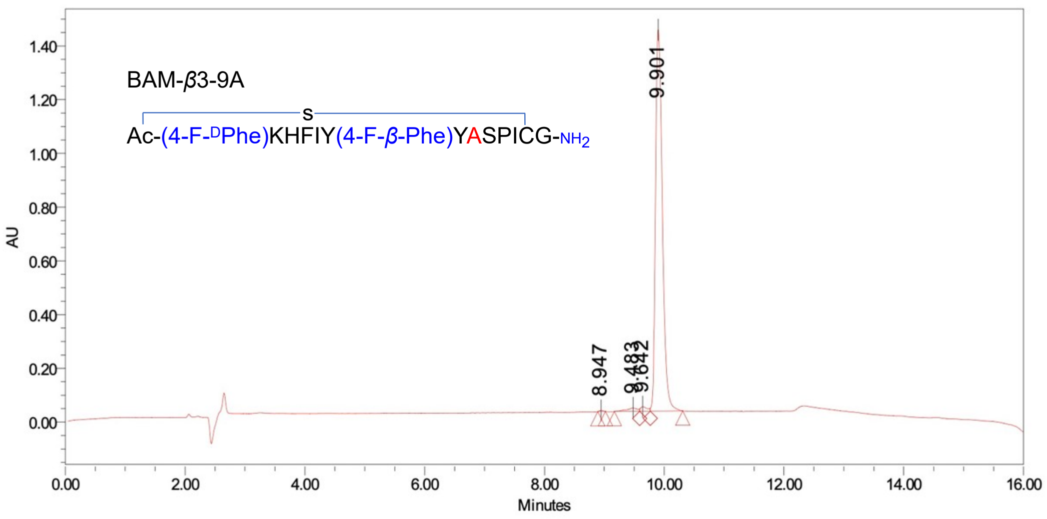
BAM-β3-9A
Ac-(4-F-DPhe)KHFIY(4-F-β-Phe)YASPICG-NH2
S
9.901
8.947
9.483
9.642
AU
Minutes

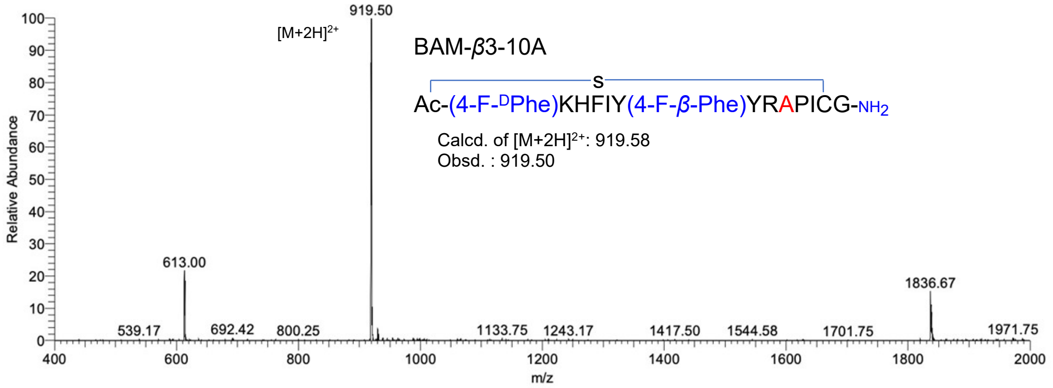
BAM-β3-10A
Ac-(4-F-DPhe)KHFIY(4-F-β-Phe)YRAPICG-NH2
S
Calcd. of [M+2H]2+: 919.58
Obsd. : 919.50
[M+2H]2+
919.50
613.00
1836.67
539.17
692.42
800.25
1133.75
1243.17
1417.50
1544.58
1701.75
1971.75
Relative Abundance
m/z

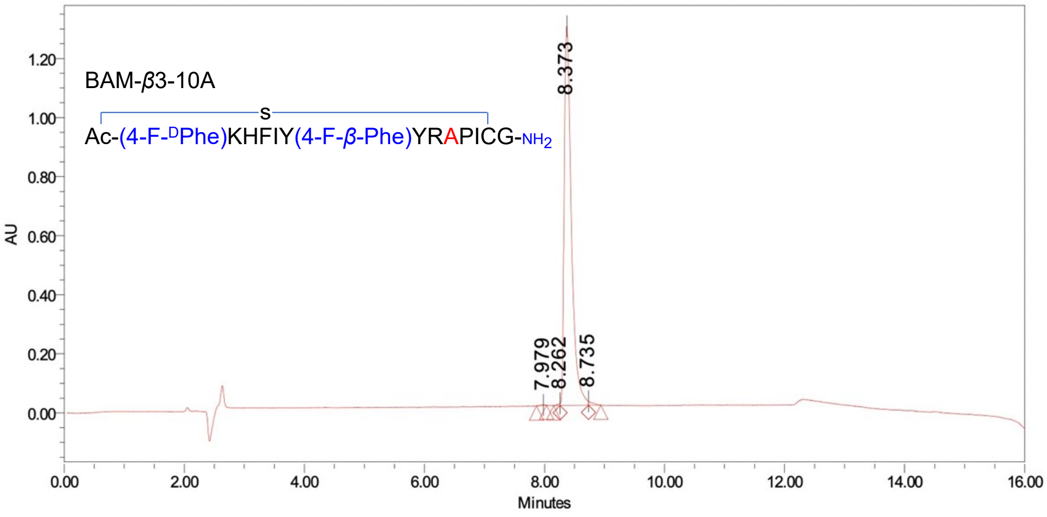
BAM-β3-10A
Ac-(4-F-DPhe)KHFIY(4-F-β-Phe)YRAPICG-NH2
S
8.373
7.979
8.262
8.735
AU
Minutes

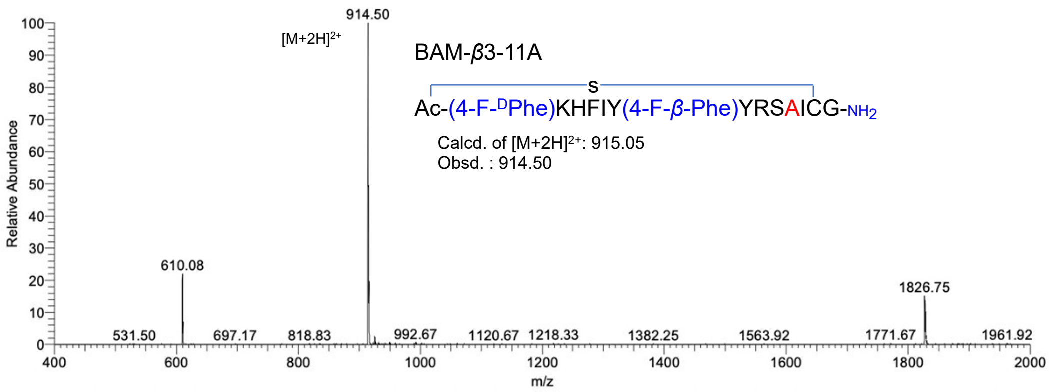

BAM-β3-11A
Ac-(4-F-DPhe)KHFIY(4-F-β-Phe)YRSAICG-NH2
Calcd. of [M+2H]2+: 915.05
Obsd. : 914.50
[M+2H]2+
914.50
610.08
1826.75
531.50
697.17
818.83
992.67
1120.67
1218.33
1382.25
1563.92
1771.67
1961.92
Relative Abundance
m/z

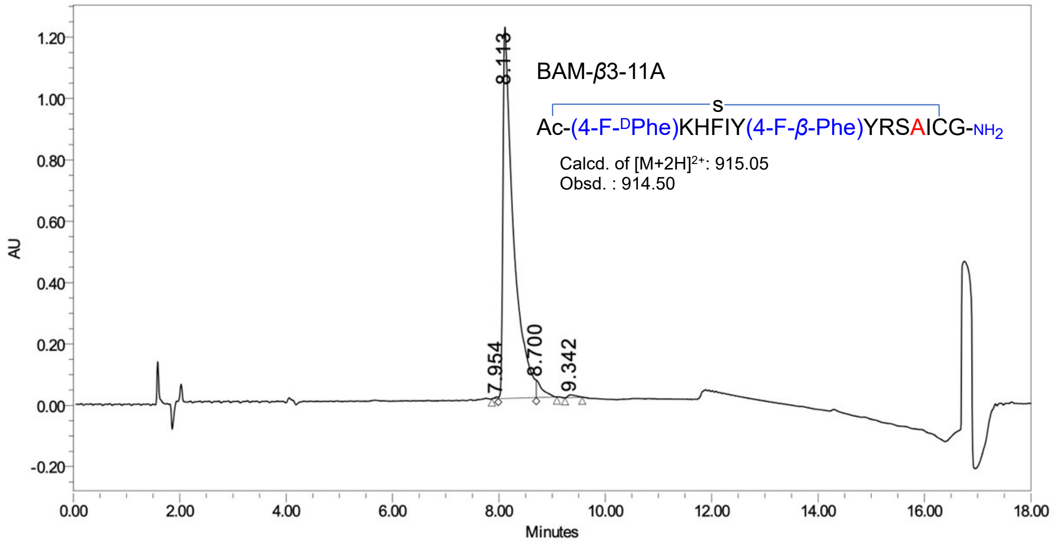

BAM-β3-11A
Ac-(4-F-DPhe)KHFIY(4-F-β-Phe)YRSAICG-NH2
Calcd. of [M+2H]2+: 915.05
Obsd. : 914.50
8.113
7.954
8.700
9.342
AU
Minutes

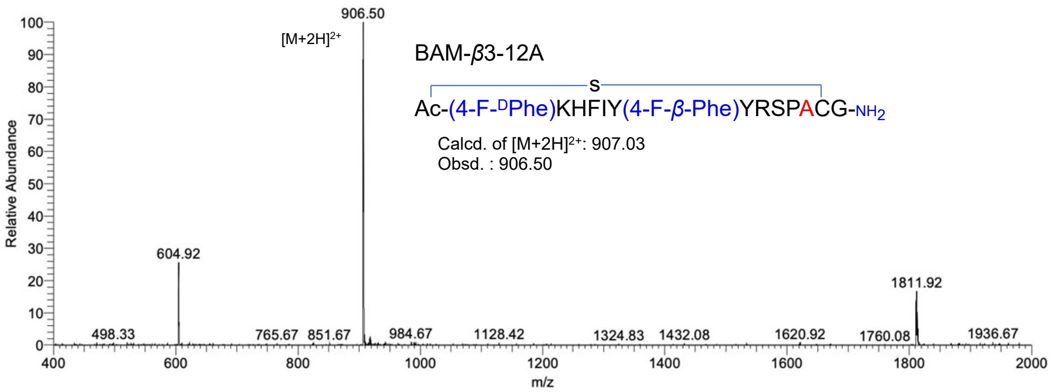

BAM-β3-12A
Ac-(4-F-DPhe)KHFIY(4-F-β-Phe)YRSPACG-NH2
Calcd. of [M+2H]2+: 907.03
Obsd. : 906.50
[M+2H]2+
906.50
604.92
1811.92
498.33
765.67
851.67
984.67
1128.42
1324.83
1432.08
1620.92
1760.08
1936.67
Relative Abundance
m/z

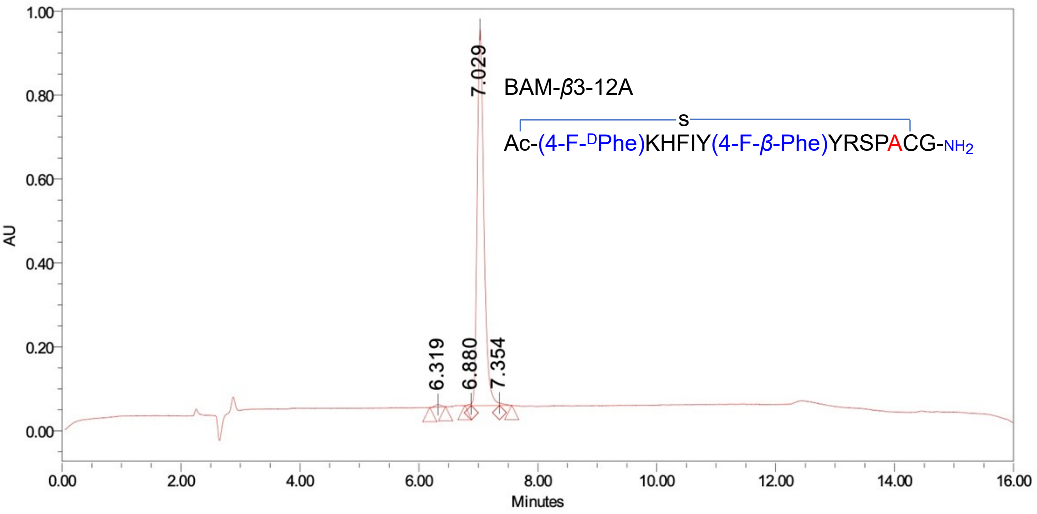
BAM-β3-12A
S
Ac-(4-F-DPhe)KHFIY(4-F-β-Phe)YRSPACG-NH2
7.029
6.319
6.880
7.354
AU
Minutes

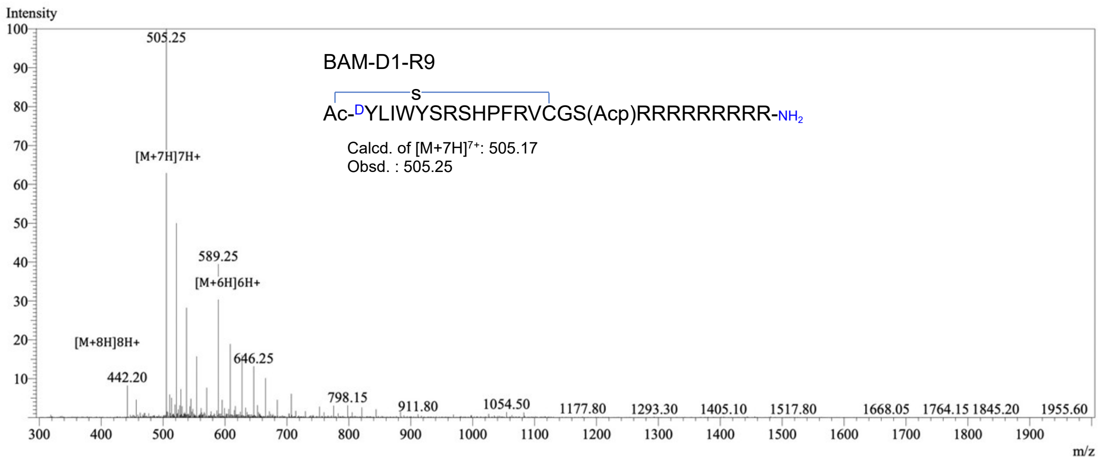
Intensity
BAM-D1-R9
S
Ac-DYLIWYSRSHPFRVCGS(Acp)RRRRRRRRR-NH2
Calcd. of [M+7H]7+: 505.17
Obsd. : 505.25
505.25
[M+7H]7H+
589.25
[M+6H]6H+
[M+8H]8H+
442.20
646.25
798.15
911.80
1054.50
1177.80
1293.30
1405.10
1517.80
1668.05
1764.15
1845.20
1955.60
m/z

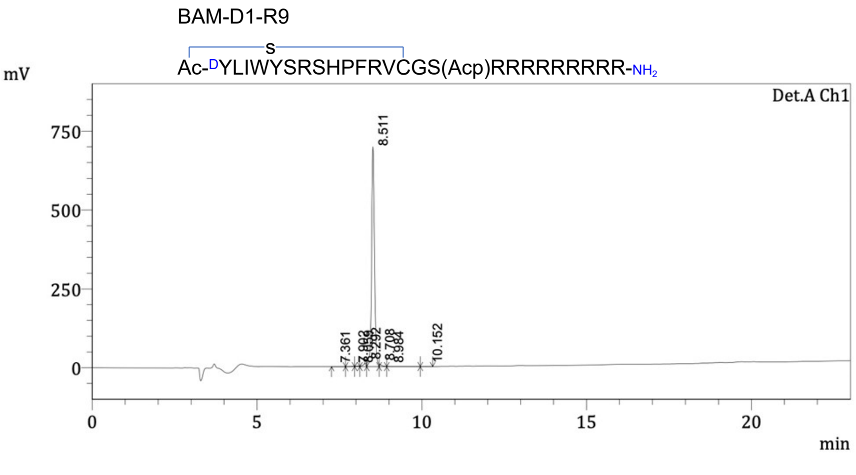
BAM-D1-R9
S
Ac-DYLIWYSRSHPFRVCGS(Acp)RRRRRRRRR-NH2
mV
Det.A Ch1
8.511
7.361
10.152
min

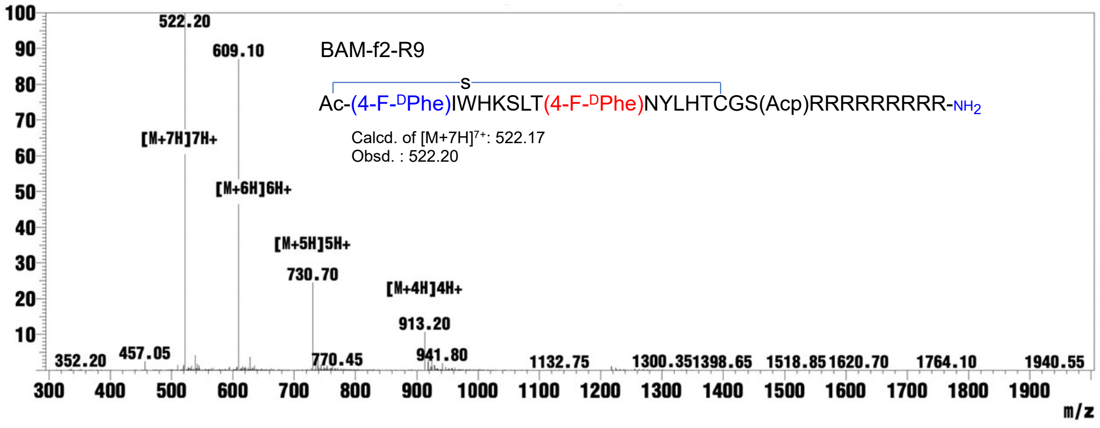
BAM-f2-R9
S
Ac-(4-F-DPhe)IWHKSLT(4-F-DPhe)NYLHTCGS(Acp)RRRRRRRRR-NH2
Calcd. of [M+7H]7+: 522.17
Obsd. : 522.20
522.20
[M+7H]7H+
609.10
[M+6H]6H+
[M+5H]5H+
730.70
[M+4H]4H+
913.20
352.20
457.05
770.45
941.80
1132.75
1300.35
1398.65
1518.85
1620.70
1764.10
1940.55
m/z

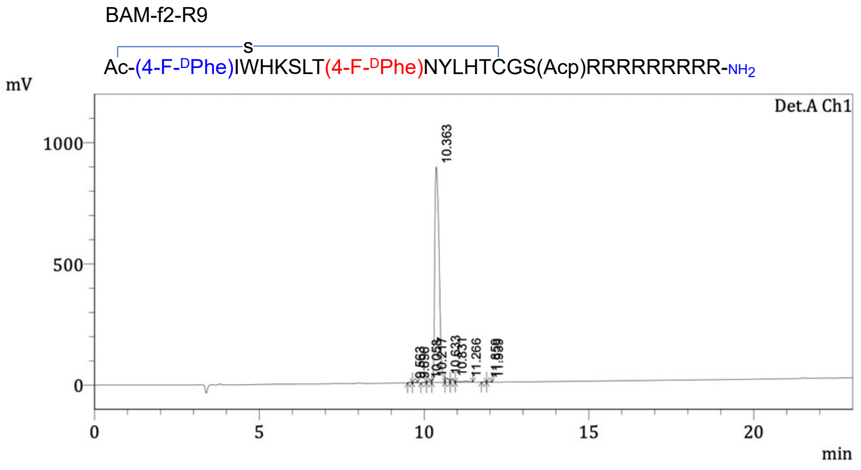
BAM-f2-R9
S
Ac-(4-F-DPhe)IWHKSLT(4-F-DPhe)NYLHTCGS(Acp)RRRRRRRRR-NH2
mV
Det.A Ch1
10.363
11.266
min

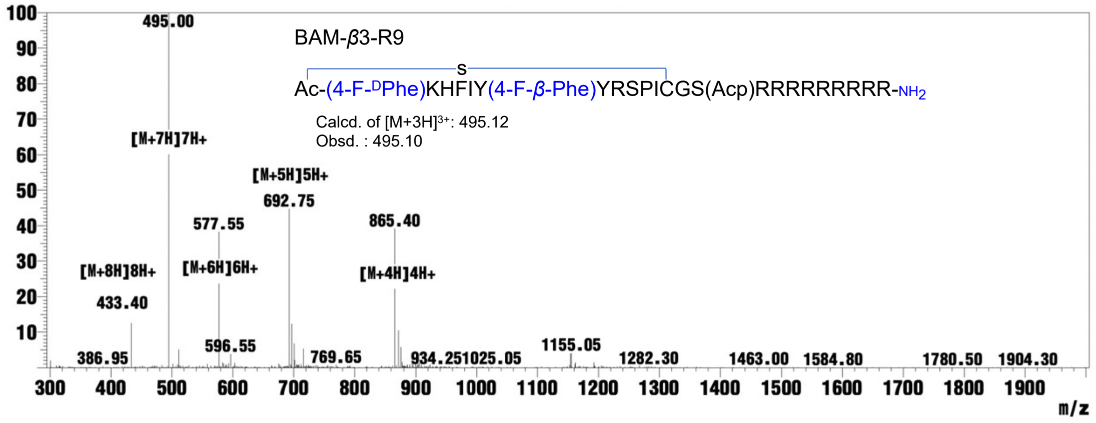
BAM-β3-R9
S
Ac-(4-F-DPhe)KHFIY(4-F-β-Phe)YRSPICGS(Acp)RRRRRRRRR-NH2
Calcd. of [M+3H]3+: 495.12
Obsd. : 495.10
495.00
[M+7H]7H+
[M+5H]5H+
692.75
577.55
865.40
[M+8H]8H+
433.40
[M+6H]6H+
[M+4H]4H+
386.95
596.55
769.65
934.25
1025.05
1155.05
1282.30
1463.00
1584.80
1780.50
1904.30
m/z

BAM-β3-R9

Ac-(4-F-^{D}Phe)KHFIY(4-F-β-Phe)YRSPICGS(Acp)RRRRRRRRR-NH_2

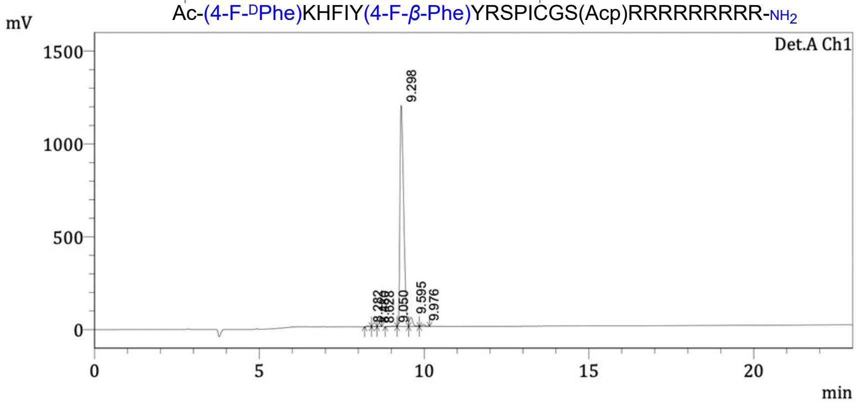

References

(1) H. Murakami, A. Ohta, H. Ashigai, H. Suga, *Nat. Methods* **2006**, *3*, 357-359.

(2) Y. Yamagishi, I. Shoji, S. Miyagawa, T. Kawakami, T. Katoh, Y. Goto, H. Suga, *Chem. Biol.* **2011**, *18*, 1562-1570.

(3) Y. Goto, T. Katoh, H. Suga, *Nat. Protoc.* **2011**, *6*, 779-790.

(4) T. Katoh, Y. Iwane, H. Suga, **2017**, *45*, 12601-12610.

(5) J. Morimoto, Y. Hayashi, H. Suga, *Angew. Chem. Int. Ed.* **2012**, *51*, 3423-3427.
